# Electrolyte‐Induced Interfacial/Bulk Dual Regulation Enables Negligible Capacity Decay in Li‐Rich Cathodes

**DOI:** 10.1002/adma.72969

**Published:** 2026-04-06

**Authors:** Tianqi Yang, Min Jiang, Jiatao Lou, Zhouyu Huang, Xingjun Li, Liuqi Wang, Qingru Zhou, Lun Li, Liuyi Hu, Wei Liu, Yuzhi He, Xingyu Wang, Zhengbo Liu, Wenkui Zhang, Jun Zhang, Xinhui Xia, Yang Ren, Qi Liu

**Affiliations:** ^1^ Department of Physics City University of Hong Kong Hong Kong China; ^2^ Tsinghua Shenzhen International Graduate School Tsinghua University Shenzhen China; ^3^ State Key Laboratory of Advanced Separation Membrane Materials, College of Materials Science and Engineering Zhejiang University of Technology Hangzhou China; ^4^ China‐UK Low Carbon College Shanghai Jiao Tong University Shanghai P.R. China; ^5^ State Key Laboratory of Chemical Engineering Department of Chemistry Zhejiang University Hangzhou China; ^6^ Shenzhen Research Institute City University of Hong Kong Shenzhen China; ^7^ Department of Physics JCSTEM Lab of Energy and Materials Physics City University of Hong Kong Hong Kong China

**Keywords:** capacity decay, interfacial degradation, Jahn‐Teller distortion, Li‐rich cathode, electrolyte‐induced

## Abstract

Lithium‐rich manganese‐based oxides (LRMO) suffer from rapid capacity decay, mainly driven by interfacial instability and bulk structural degradation associated with Jahn‐Teller (J‐T) distortion in Mn^3+^‐rich regions. Such distortion accelerates surface oxygen activity, triggers nonuniform cathode electrolyte interphase (CEI) formation along with promoted parasitic reactions. Herein, we develop an electrolyte‑induced interfacial/bulk dual regulation strategy that enables negligible capacity decay in Li‑rich cathodes via coordinated interfacial/bulk regulation. In situ characterizations combined with interfacial compositional analyses confirm the dynamic formation of a thin, uniform, and robust LiF/LiBO_2_‐rich CEI, which stabilizes surface oxygen species and suppresses interfacial side reactions. Meanwhile, local structural analyses combined with theoretical calculations reveal that fluorinated molecules regulate Mn into a low‐spin configuration, thereby alleviating J‐T distortion and preventing bulk structural degradation. Benefiting from this dual induced interfacial‐bulk stabilization effect, LRMO||Li cells deliver an initial capacity of 219.6 mAh g^−1^ and retain 97.6% of their capacity after 400 cycles. This work provides a new pathway toward electrolyte‐mediated dual stabilization and demonstrates the feasibility of mitigating capacity decay in Li‐rich cathodes via electrolyte‐induced interfacial/bulk regulation.

## Graphical abstract

This work report a n electrolyte‑induced interfacial/bulk dual regulation strategy that enables negligible capacity decay in li‑rich cathodes.

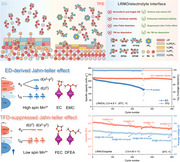

## 1 Introduction

Over the past decade, the extensive combustion of fossil fuels has dramatically increased CO_2_ emissions and intensified global warming [1, 2]. This situation has accelerated the worldwide promotion of electric vehicles (EVs) as a key strategy for reducing carbon emissions [3]. The success of EVs largely depends on the development of high‐energy‐density lithium‐ion batteries (LIBs) [4]. Among the four components of LIBs (cathode, anode, electrolyte and separator), it is the cathode materials that determines the energy density and cost of the LIBs [5, 6, 7]. Nevertheless, commercial LIBs system relying on traditional cathode materials suffer from limited energy density. Conventional cathodes such as olive LiFePO_4_ [8, 9], spinel LiMn_2_O_4_ [10] and ternary LiNi_x_Co_y_Mn_z_O_2_ [11, 12, 13, 14], deliver capacities of only 120–200 mAh g^−1^ based on cationic redox reactions, which have nearly reached their theoretical limits [15, 16]. In this regard, lithium‐rich manganese‐based oxide (LRMO) cathode materials, with the formula written as *x*Li_2_MnO_3_·(1−*x*)LiTMO_2_ (TM = Ni, Co, Mn, etc.) [17], stand out due to their unique anionic redox chemistry that goes beyond the traditional cationic redox reactions. LRMO cathode can deliver exceptionally high specific capacities exceeding 250 mAh g^−1^, significantly surpassing those of conventional cathodes [18, 19, 20]. This high capacity, combined with a high operating voltage, enables LRMO‐based LIBs to achieve gravimetric energy densities projected to reach 400–450 Wh kg^−1^, which is crucial for meeting the demands of high‐energy‐density EVs‐used LIBs [21, 22, 23].

Despite the excellent prospects, LRMO suffer both voltage decay and capacity decay during cycling, which differ from the degradation modes of traditional cathodes and remain critical challenges [24, 25, 26]. Specifically, voltage decay is primarily associated with the local structural instability of the bulk material [27]. This instability originates from the honeycomb local structure in the monoclinic Li_2_MnO_3_ component of the LRMO [28], which undergoes irreversible oxidation and lattice oxygen loss during high‐voltage charging. The resulting oxygen vacancies facilitate the migration of transition metal (TM) ions into lithium layers, inducing phase transitions and causing continuous voltage decay. In contrast, capacity decay is mainly an interface‐related issue [29]. Although electrolyte decomposition under high voltage contributes to formation of inhomogeneous cathode electrolyte interphase (CEI) and harmful byproduct (e.g., ROCO_2_Li, HF), its initiation and acceleration are strongly coupled to Jahn‐Teller (J‐T) distortion [30]. This coupling becomes particularly critical in Mn^3+^‐rich regions during the Mn ion redox reaction. The J‐T distortion generates severe local lattice strain, which induces microcracks on the LRMO surface and exposes it to electrolyte attack, leading to accelerate electrolyte decomposition. Meanwhile, TM ion dissolution, especially Mn^2+^ ion via J‐T distortion induced disproportionation, becomes pronounced [31]. These side reactions collectively lead to the formation of thick and inhomogeneous cathode electrolyte interphases, as well as electrochemically inert rock‐salt phases [32]. Thus, the degraded interface impedes Li^+^ transport and increases impedance at LRMO cathode/electrolyte interface, ultimately leading to continuous capacity decay. In summary, the underlying mechanism of voltage decay and capacity decay are distinct. Voltage decay stems from bulk structural instability and is best mitigated by doping strategies [33, 34], whereas capacity decay is driven by J‐T effect accelerated interfacial degradation and requires interfacial engineering solutions.

To address capacity decay, researchers have explored two major approaches: surface coating and electrolyte engineering. Surface coating strategies using materials such as LiF [35], and Li_3_PO_4_ [36] can not only establish protective layers on the cathode surface but also frequently induce in situ spinel interfacial phases with high lattice compatibility [37, 38], thereby synergistically suppressing detrimental side reactions such as oxygen release, TM ions dissolution, and structural degradation. In details, LiF coatings homogenize Li^+^ fluxes and modulate electric field distribution; Li_3_PO_4_ layers act as ion‐conductive barriers that shield particles from corrosion while reducing interfacial strain and phase distortion; and spinel coatings reinforce surface TM─O bonds, elevate the energy barrier for oxygen vacancy formation, and alleviate MnO_6_ octahedral distortion and J‐T effects, thereby stabilizing both the interface and bulk of LRMO cathode. Nevertheless, conventional coatings suffer from poor uniformity, low ionic conductivity, and mechanical fragility, making them prone to peeling and incapable of self‐healing, which ultimately undermines long‐term cycling stability. In contrast, electrolyte engineering enables in situ CEI formation and evolution [39, 40], effectively overcoming these mechanical limitations of ex‑situ surface coatings and offering a more robust pathway to interface stabilization. Representative additives exemplify how interfacial chemistry can be dynamically tailored: tris (trimethylsilyl) borate (TMSB) generates LiF, Li_3_PO_4_, and borate species to construct an armor‑like gradient CEI [41]; tris(2,2,2‐trifluoroethyl) phosphate (TFP), an electrophilic phosphate, anchors reactive oxygen intermediates and yields a thin, fast‑ion‑conducting CEI with high initial Coulombic efficiency [42]; and 2,4,6‐trivinyl‐2,4,6‐trimethylcyclotrisiloxane (TVTMS) polymerizes into a conformal hetero‑chain CEI that balances rigidity with energy dissipation [20]. Yet despite these advances, their impact on capacity decay remains limited because they fail to address the atomic‐scale origin of interfacial degradation, namely the formation of J‐T distorted interfaces in Mn^3+^‐rich regions. Therefore, more sophisticated strategies guided by a multi‑scale design principle are required to simultaneously mitigate capacity decay at both the mesoscopic interface and the atomic electronic structure level.

**FIGURE 1:**
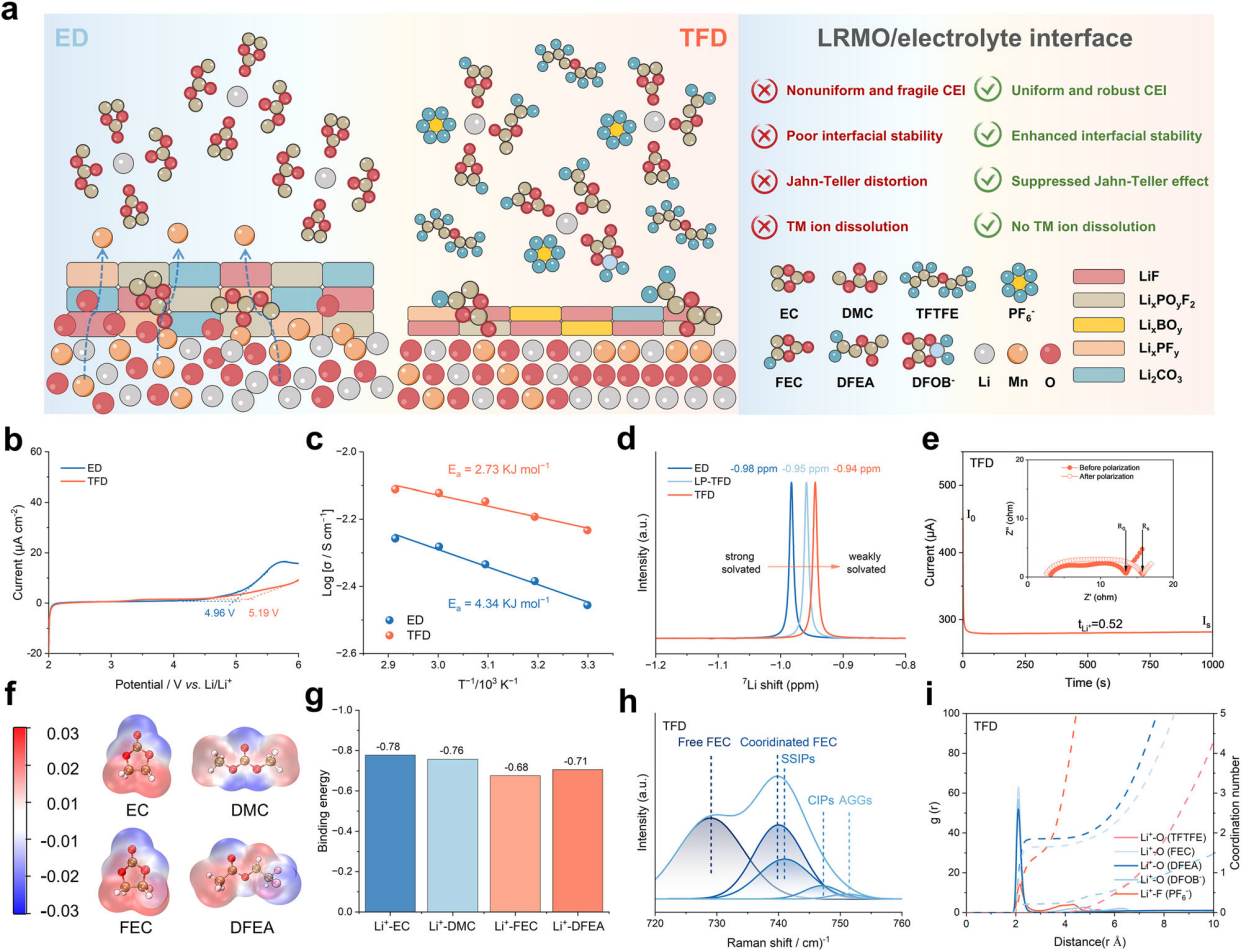
(a) Schematic illustration of the LRMO/electrolyte interface design with ED and TFD electrolytes. (b) Linear sweep voltammograms of ED and TFD electrolytes. (c) Ionic conductivities of ED and TFD electrolytes at various temperatures, the activation energies are calculated according to Arrhenius formula. (d) ^7^Li NMR spectra of ED and TFD electrolytes. (e) Current‐time curves following DC polarization for Li/TFD/Li cell, the inset corresponds to AC impedance spectra before and after DC polarization. (f) Electrostatic potential distribution for typical non‐fluorinated/fluorinated cyclic and linear carbonate molecules (EC, DMC, FEC and DFEA). (g) Binding energies between Li^+^ and typical solvent molecules. (h) Raman spectra fitting result for TFD electrolyte. (i) Radial distribution functions and coordination numbers of Li^+^ from MD simulations of TFD.

Targeting the above challenges, we develop a tailored electrolyte (referred to as TFD) that enables simultaneous interfacial and bulk regulation. As illustrated in Figure 1a, commercial EC‑based carbonate electrolytes (referred to as ED) form a nonuniform and mechanically fragile CEI on LRMO, while their solvent molecules fail to suppress Mn^3+^‐triggered J‐T distortion, leading to accelerated TM ions dissolution and progressive structural degradation. In contrast, the tailored molecular components in TFD induce a thin, uniform and robust LiF/LiBO_2_‑rich CEI through controlled decomposition pathways, ensuring effectively interfacial passivation. More importantly, the coordinated molecular environment in TFD modulates the local electronic structure of LRMO and effectively mitigates Mn^3+^‑associated spin‑state instability, thereby suppressing bulk lattice distortion at its origin. Benefiting from this interfacial/bulk dual regulation, both LRMO||TFD||Li and LRMO||TFD||Graphite pouch cells exhibit markedly improved cycling stability with negligible capacity decay. This work demonstrates a electrolyte‑induced stabilization strategy that overcomes the long‑standing capacity decay of Li‑rich cathodes.

## 2 Results and Discussion

### 2.1 Electrochemical Tests of TFD

It is well established that the Li_2_MnO_3_ phase in LRMO cathodes requires to be activated at voltages above 4.5 V, and the LRMO||Li cell sustained operation under a high cut‐off voltage of 4.8 V to leverage the advantages of high energy density. To satisfy the high‐voltage operating requirements of LRMO cathodes, the electrolyte is required to possess strong anti‐oxidization stability. Thus, the electrochemical stability window (ESW) of electrolyte was initially assessed employing linear sweep voltammetry (LSV) method. As shown in Figure 1b, the ESW of TFD (5.19 V) exceeds that of ED (4.96 V), confirming that TFD provides superior anti‐oxidization stability and thereby enables the reliable operation of LRMO cathodes under high cut‐off voltage up to 4.8 V.

To further characterize the electrolyte properties, differences along ion transport properties and solvation structures were further revealed via basic electrochemical test and coupled analysis. Electrochemical impedance spectra (EIS) measurement conducted over a range of temperatures show that the ionic conductivity of ED is lower than that of TFD, consistent with its higher activation energy (4.34 KJ mol^−1^) derived from the Arrhenius equation compared with that in TFD (2.73 KJ mol^−1^). This indicate that ion migration in ED requires overcoming larger energetic barriers (Figure 1c; Figure S1). Complementary to the EIS results, the ^7^Li nuclear magnetic resonance (NMR) spectra reveal a systematic evolution of the Li^+^ environment. By comparing the ED, 1.2 M LiPF_6_ in TFTFE/FEC/DFEA (v/v/v = 1:1:2) (referred to as LP‐TFD), and TFD electrolytes, a progressive downfield shift of the resonance peak is observed from −0.98 ppm (ED) to −0.95 ppm (LP‐TFD) and further to −0.94 ppm (TFD) (Figure 1d). This trend signals a systematic reduction in local electronic shielding (deshielding effect), reflecting a transition from a strongly solvated Li^+^ configuration in the conventional ED to a weakly coordinated chemical environment in the fluorinated systems. This finding suggests a transition from a strongly solvated Li^+^ configuration in ED to a weakly solvated structure in TFD. This unique solvated structure is also expected to promote Li^+^ migration, as evidenced by the significantly higher lithium‐ion transfer number in TFD (0.52, Figure 1e) compared to ED (0.39, Figure S2), suggesting that TFD enables more efficient Li^+^ transport. Overall, these results collectively demonstrate that TFD provides superior anti‐oxidization stability and more favorable Li^+^ transport properties compared to ED.

Furthermore, density functional theory (DFT) calculations and molecular dynamics (MD) simulations were conducted to elucidate the underlying mechanism of these differences. First, electrostatic potential (ESP) mapping of solvent molecules first revealed their electron cloud density distributions and potential Li^+^ binding sites (Figure 1f; Figure S3a). The carbonyl oxygen atom exhibited the most negative ESP, indicating its preferential coordination with Li^+^. Consistently, binding energy calculations showed that fluorinated solvents effectively weaken Li^+^ coordination and reduce solvent‐anion interactions (Figure 1g; Figures S3b and S4; Note S1), in agreement with the NMR results. These findings collectively demonstrate that fluorinated solvents in the TFD weaken Li^+^ coordination relative to conventional carbonate molecules in ED, thereby forming a weakly solvated structure. The Li^+^‐solvent coordination distance further supports this conclusion, with the Li^+^‐TFTFE distance extending to 2.075 Å (Figure S5). Such a long distance indicates negligible coordination between TFTFE with Li^+^, suggesting that TFTFE functions as an anti‐solvent that lowers electrolyte viscosity and promotes the formation of locally high‐concentration electrolyte domains, which in turn facilitates the generation of contact ion pairs (CIPs) and aggregates (AGGs) [43].

Raman spectra further validated this structural evolution (Figure 1h; Figure S6 and Note S2), with fitting results revealing the presence of additional CIPs and AGGs in the TFD electrolyte, whereas only solvent‐separated ion pairs (SSIPs) were observed in the ED electrolyte (Figure S7a). Specifically, comparative Raman spectra (Figure S7c) and MD simulations (Figure S7d) confirm that the fluorinated solvents in LP‐TFD drives anions into the primary solvation shell to displace solvent molecules, achieving a higher content of contact ion pairs (CIPs) and aggregates (AGGs). MD simulations corroborated the Raman results, showing weakened Li^+^‐solvent coordination (Figure 1i; Figure S7b) and enhanced Li^+^‐PF_6_^−^ interactions (Figure S8) in TFD. Interestingly, despite LP‐TFD possess the highest Li^+^‐PF_6_^−^ coordination number (Figure S8), the TFD electrolyte exhibits the most pronounced downfield shift in ^7^Li NMR spectra, suggesting a more stable weakly solvated environment. Last but not least, the desolvation energies of representative SSIPs and CIPs, extracted from the MD results of ED and TFD, were calculated to be −2.437 eV and −2.269 eV (Figure S9), respectively, indicating that Li^+^ is more readily desolvated in the weakly solvated TFD environment.

Collectively, these experimental and theoretical results demonstrate that the rationally designed TFD electrolyte possesses enhanced anti‐oxidization stability and a unique weakly solvated structure enriched by CIPs and AGGs, thereby offering a performance advantage for LRMO cathode based high‐energy‐density LIBs.

### 2.2 Compatibility With Metallic Li and SEI Analysis

**FIGURE 2:**
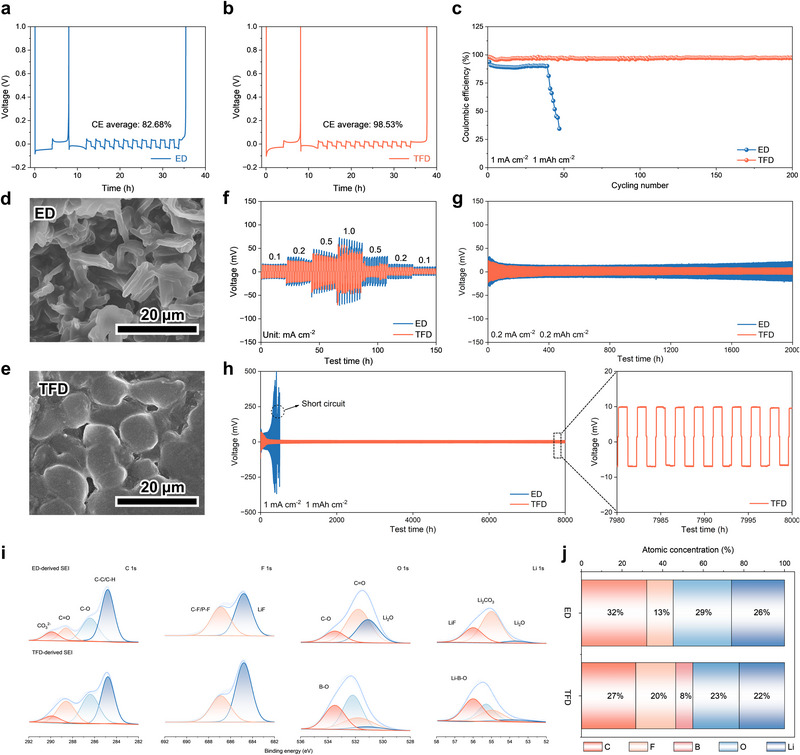
The Aurbach measurement of Li plating/stripping Coulombic efficiency in Li||Cu cells assembled with (a) ED and (b) TFD. (c) Li plating/stripping Coulombic efficiency in Li||Cu cells assembled with ED and TFD at a current density and areal capacity of 1 mA cm^−2^, 1 mAh cm^−2^. FE‐SEM images of Li deposition obtained by plating 4 mAh cm^−2^ of Li onto a Cu substrate using (d) ED and (e) TFD in Li||Cu cells at a current density of 0.5 mA cm^−2^. (f) Rate performance of Li||Li symmetric cells with ED and TFD at various current densities. Li plating/stripping profiles of symmetric Li/Li cells with FSNE and GSNE at a current density and areal capacity of (g) 0.2 mA cm^−2^, 0.2 mAh cm^−2^, and (e) 1 mA cm^−2^, 1 mAh cm^−2^. (i) Detailed fitting results of XPS spectra for C, F, O, and Li elements. (j) The atomic concentration of SEI on cycled Li with ED and TFD as electrolytes.

Given that the initial reduction reactions governed by the solvation structure also influence metallic Li, we further examined its deposition/stripping behavior and SEI formation. Li||Cu cells were assembled with different electrolytes and evaluated using Aurbach Coulombic Efficiency (CE) tests at a current density of 1 mA cm^−2^. The initial plating capacity was fixed at 4 mAh cm^−2^, followed by cycling with a capacity of 1 mAh cm^−2^ (Figure 2a,b). Using ED and TFD electrolytes, the Li||Cu cells achieved CE values of 82.68% and 98.53%, respectively. Moreover, Li||TFD||Cu cell exhibit lower nucleation and growth overpotentials, confirming that TFD effectively promotes uniform Li plating/stripping behaviors (Figure S10). Further long‐term cycling tests were conducted at a cutoff capacity of 1 mAh cm^−2^ and a current density of 1 mA cm^−2^. The results revealed that TFD maintained an average CE of 97.01% over 20 cycles (Figure 2c), whereas ED experienced rapid decay within fewer than 50 cycles. SEM images elucidated the origin of interfacial stability differences: with ED, Li deposition on the Cu surface was highly uneven and accompanied by pronounced dendrite formation, while TFD enabled more uniform and compact Li deposition with larger, smoother grains, thereby reducing the effective contact area between the electrolyte and metallic Li (Figure 2d,e).

To further evaluate dendrite suppression and interfacial stability, Li||Li symmetric cells were assembled. Rate performance tests were performed under different current densities with a fixed plating/stripping time of 1 h (Figure 2f). The results demonstrated that Li||Li cells with TFD exhibited superior interfacial stability, showing an overpotential of only 50 mV at 1 mA cm^−2^ and consistently lower overpotentials across all current densities. Long‐term cycling at 0.2 mA cm^−2^ with a cutoff capacity of 0.2 mAh cm^−2^ confirmed that TFD‐based Li/Li cells could stably operate for 2000 h without short‐circuiting or significant voltage fluctuations (Figure 2g). In contrast, ED‐based cells showed continuous overpotential increase after the initial activation stage, indicating progressive interfacial degradation despite the absence of short‐circuiting. As expected, under harsher conditions (1 mA cm^−2^, 1 mAh cm^−2^ cutoff capacity), Li||ED||Li cells exhibited sharp overpotential rise and short‐circuit failure within several hundred hours, whereas TFD maintained stable cycling for over 8000 h with a steady overpotential of ∼10 mV (Figure 2h). XPS analysis of cycled Li surfaces further clarified the differences in solid electrolyte interphase (SEI) composition. The full spectra revealed significantly higher F element content and lower C, O element content on Li cycled in TFD compared to ED (Figure 2j; Figure S11), indicating fewer organic byproducts. High‐resolution spectra confirmed the formation of LiF/LiBO_2_ rich SEI in TFD. Crucially, the B 1s spectra of the TFD‐cycled Li anode exhibited prominent B─F and B─O signals, providing direct evidence for the formation of a boron‐containing SEI. This can be attributed to the preferential reductive decomposition of LiDFOB, which facilitates the formation of a thin and uniform SEI. Such a high‐quality SEI effectively mitigates the persistent consumption of electrolyte components and inhibits deleterious interfacial side reactions, with reduced amounts of Li_2_O, Li_2_CO_3_, and organic species (Figure 2i; Figure S12). Together, the XPS and SEM results corroborate the superior compatibility of TFD with metallic Li, mitigating interfacial degradation at the metallic Li anode.

### 2.3 Electrochemical Performance Under Various Conditions

**FIGURE 3:**
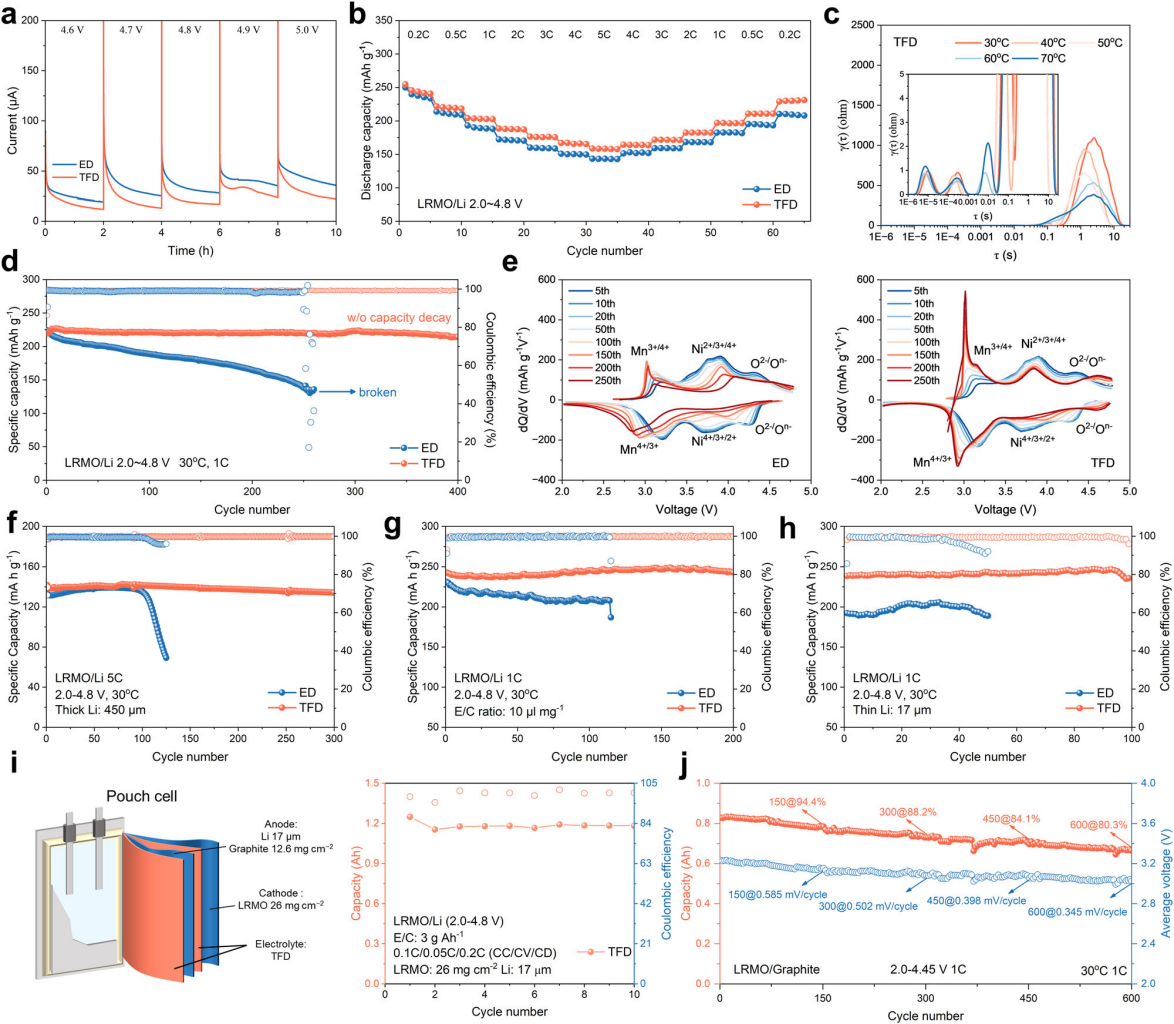
(a) Electrochemical floating test of ED and TFD electrolytes using the LRMO cathode. (b) Rate performance of LRMO||ED||Li and LRMO||TFD||Li cells. (c) DRT results for LRMO||TFD||LRMO symmetrical cell at various temperatures. (d) Cycling performance of LRMO||ED||Li and LRMO||TFD||Li cells at 1 C (1C = 200 mA g^−1^). (e) Corresponding dQ/dV profiles extracted from LRMO||Li cells cycled in ED and TFD LRMO||Li cells cycled at 1 C under extreme conditions (f) high rate (5 C), (g) Low E/C ratio (10 µL mg^−1^), and (h) Thin Li (17 µm). (i) Schematic illustration and performance for the LRMO||Li pouch cell. (j) Cycling performance for the LRMO||TFD||Graphite pouch cell at 1 C and 2.0–4.45 V (equal to 4.50 V vs. Li^+^/Li).

With the interfacial compatibility on metallic Li clarified, attention was then shifted to understanding how the distinct solvation structures and transport properties of the electrolytes govern the electrochemical behavior of LRMO||Li cells. To this end, electrochemical floating tests were first conducted to measure leakage currents under various constant voltages, thereby probing the oxidative decomposition tendencies of the electrolytes when paired with LRMO cathodes under realistic operating conditions (Figure 3a). The results show that the TFD electrolyte exhibits markedly lower leakage currents and enhanced oxidative stability, whereas the ED electrolyte displays pronounced decomposition currents, indicating weaker resistance to high‑voltage degradation. This trend is consistent with the LSV results (Figure 1b). Subsequently, rate performance tests (Figure 3b) were conducted to further assess the impact of electrolyte chemistry on Li^+^ transport kinetics. According to the charge–discharge profiles (Figure S13a), LRMO||TFD||Li cell delivers larger initial Coulombic efficiency (ICE) of 71.9% compared with that of 68.7% in LRMO||ED||Li cells. Besides, LRMO||TFD||Li batteries cell showed a specific discharge capacity of 245.6, 221.6, 204.1, 188.5, 176.3, 167.1 and 158.7 mAh g^−1^ at 0.2, 0.5, 1, 2, 3, 4 and 5C, respectively (Figure S13c). Compared with this, LRMO||ED||Li batteries cell showed a specific discharge capacity of 239.8, 213.8, 193.1, 172.4, 160.1, 150.8 and 143.4 mAh g^−1^ at at the corresponding rates (Figure S13b), confirming the superior rate performance enabled by TFD.

To elucidate the origin of this kinetic enhancement, LRMO||LRMO symmetric cells were assembled and subjected to variable‐temperature EIS test. Distribution of relaxation time (DRT) analysis of EIS results reveals that the impedance of CEI (R_CEI_), charge transfer impedance (R_ct_), and Warburg diffusion impedance (Z_w_) are significantly reduced in LRMO||TFD||LRMO cell (Figure 3c; Figure S14 and Note S3), indicating lower interfacial resistance and faster Li^+^ transport. Complementary galvanostatic intermittent titration technique (GITT) measurements further corroborated these findings, showing higher Li^+^ diffusion coefficients in LRMO||TFD||Li cells (Figure S15). Collectively, these results demonstrate that the weakened solvation structure induced by TFD enables LRMO||Li cells to achieve superior rate performance.

In terms of capacity decay, LRMO||TFD||Li cells deliver an initial capacity of 219.6 mAh g^−1^ with a capacity retention rate of 97.6% after 400 cycles at 1C, whereas LRMO||ED||Li cells undergo rapid capacity decay and fail after approximately 250 cycles (Figure 3d). Differential capacity (dQ/dV) curves (Figure 3e; Figure S16) further highlight the fundamental distinction capacity decay mechanism of LRMO cathodes in ED and TFD electrolytes. The reduction peak at around 3.7 V corresponds to reduction of Ni ions, while the peak at around 3.3 V originates from Li^+^ insertion/extraction in the Li_2_MnO_3_ component. Notably, in LRMO||ED||Li cells the reduction peak exhibits pronounced hysteresis, a signature of irreversible oxygen release that impedes partial Li^+^ reinsertion into the bulk structure and consequently lowers the ICE. In addition, the Mn and Ni redox couples show severe and persistent fluctuations in ED, in sharp contrast to the highly reversible behavior observed in TFD. Such redox instability, particularly the excessive reduction of Mn, is closely associated with the formation of high‑spin Mn^3+^ species, which are prone to J‐T distortions. These distortions introduce local structural instabilities that further amplify the redox irreversibility captured in the dQ/dV curves. To further differentiate the capacity stabilization mechanisms, a comprehensive decoupling analysis of cycling performance was conducted using the comparative electrolytes LP‐TFD and 1.0 M LiPF_6_ + 0.2 M LiDFOB in FEC/DFEA (v/v = 1:2) (referred to as FD) (Figure S17a). The results demonstrate that the synergy between solvation‐structure regulation and orbital‐level modulation in the LP‐TFD contributes an approximate 10% improvement in capacity retention rate with 84.9% for ED and 94.2% for LP‐TFD while cycled at 1C, respectively, acting as the basis for capacity stabilization. Subsequently, the FD electrolyte was evaluated to verify the universality of these mechanisms. Despite the absence of a localized high‐concentration electrolyte (LHCE) structure due to the lack of TFTFE molecule, LRMO||FD||Li cells achieves an enhanced capacity retention rate of 97.3%. This confirms that the effective integration of electronic‐level modulation (mediated by FEC/DFEA) and the physical protection provided by the LiDFOB‐derived CEI can substantially stabilize the electrode structure across different solvent environments, serving as a critical reinforcement to the overall stability. The aforementioned performance decoupling analysis underscores that solvation‐structure regulation, orbital‐level electronic modulation, and the interfacial decomposition via LiDFOB work in parallel to establish a synergistically stable electrochemical environment. In this framework, the molecular‐induced spin‐state regulation provides the necessary internal foundation for structural durability, while the LiF‐rich CEI offers indispensable external physical reinforcement. This interfacial and bulk dual regulation ensures the structural stability of the LRMO cathode, ultimately enabling stable cycling with negligible capacity decay.

The cycling stability of LRMO||Li cells was evaluated under demanding operating conditions, including ultrahigh rate (5C, Figure 3f), low electrolyte/cathode ratio (10 µL mg^−1^, Figure 3g), thin Li anodes (17 µm, Figure 3h) and low temperature (−15°C, Figure S17b). In all cases, LRMO||TFD||Li cells exhibited markedly improved performance and reduced capacity decay. Finally, pouch cell tests further confirming the effectiveness of TFD in mitigating capacity decay under practical operating conditions. The 1.2 Ah LRMO||TFD||Li pouch cell showed no capacity decay within 10 cycles (Figure 3i). To eliminate the influence of anode‐side dead lithium effect, we evaluated LRMO||Graphite pouch cells at an aggressive cut‐off voltage of 4.75 V (equivalent to 4.8 V *vs*. Li^+^/Li). The LRMO||TFD||Graphite pouch cell still maintain significantly better stability than the LRMO||ED||Graphite pouch cell, accompanying with a significantly higher initial Coulombic Efficiency and a smaller voltage polarization (Figure S18a,b). Synchronized post‐mortem SEM analysis (Figure S18c) identifies cathode structural integrity as the primary degradation source. In the LRMO||ED||Graphite pouch cell, the cycled LRMO cathode exhibit severe surface cracking and structural disintegration, due to the unmitigated J‐T distortions. This failure facilitates TM ions leaching, which subsequently migrates to and poisons the graphite surface with thick, rugged decomposition debris. Conversely, the TFD electrolyte intercepts this degradation at the source. The cycled LRMO cathode in LRMO||TFD||Graphite remains well‐defined, with visible primary grain boundaries and no micro‐cracks. In summary, these comparative results confirm the degradation source in the baseline electrolyte is due to the cathode side rather than the anode side. Based on this cathode stabilization mechanism, the 0.8 Ah LRMO||TFD||Graphite pouch cell maintained 91.8% capacity retention after 100 cycles at 0.33C and 80.3% retention after 600 cycles at 1C under more conventional operating conditions at the cut‐off voltage of 4.45 V (equivalent to 4.5 V *vs*. Li^+^/Li), respectively (Figure 3j; Figure S18d). Collectively, these findings provide compelling evidence that the capacity decay mechanisms of LRMO differ fundamentally in that of ED and TFD electrolytes, and laying the groundwork for the following mechanistic investigations of capacity decay.

Beyond electrochemical performance, the TFD electrolyte significantly enhances the thermal safety of the high‐voltage LRMO. As evidenced by differential scanning calorimetry (DSC) results for the charged cathode (4.8 V) without electrolyte (Figure S18e), the TFD‐treated sample exhibits a suppressed and moderated heat flow compared to the ED baseline, indicating that TFD helps maintain intrinsic lattice stability and retards abrupt structural collapse at high temperatures. When in direct contact with the electrolyte (Figure S18f), the TFD transforms the violent, single‐peak exothermic reaction observed in the ED into a managed, step‐wise decomposition process. The intensity of the main exothermic peak is drastically reduced, and the early minor peak in the TFD suggests a beneficial pre‐passivation that reinforces the interface before bulk thermal runaway. Additionally, flammability tests (Figure S18g) confirm the non‐flammability of the TFD, contrasting sharply with the highly combustible ED. Collectively, the reduction in peak heat flow and the flame‐retardant nature of the electrolyte significantly mitigate the risk of thermal runaway, validating the practical viability of TFD‐stabilized LRMO cathodes.

### 2.4 CEI Dynamic Evolution and Components

**FIGURE 4:**
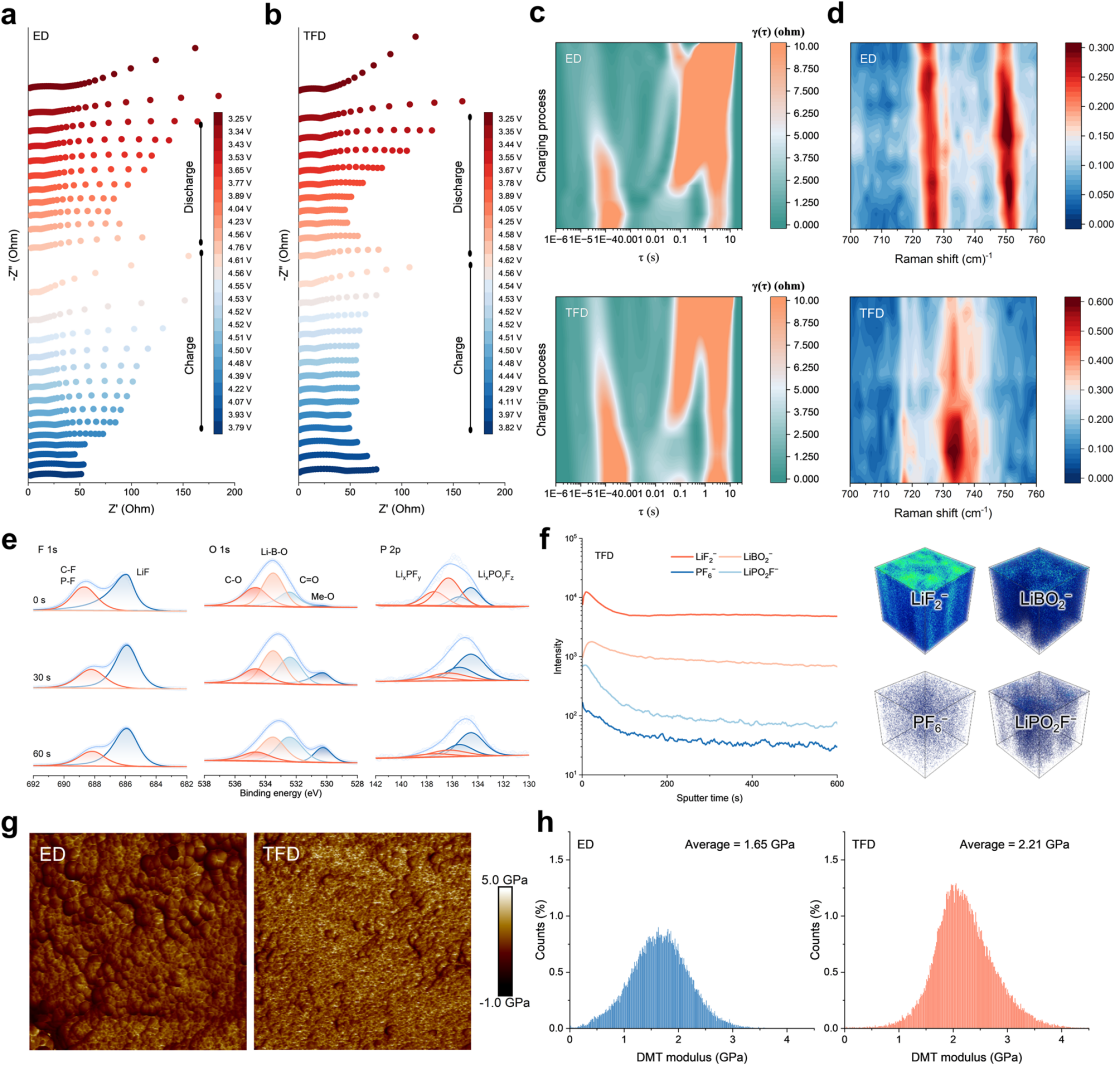
In situ GEIS of LRMO||Li cells to reveal the dynamic evolution of CEI during the first charge/discharge process cycled in (a) ED, and (b) TFD electrolytes. (c) Corresponding DRT results for In‐situ GEIS of the first charging process. (d) In situ Raman spectra of LRMO||Li cells to reveal the dynamic evolution of solvated structure in ED and TFD electrolytes. (e) XPS depth profiles of the LRMO cathode after 30 cycles in TFD. (f) Sputter depth profiles of various secondary ions obtained by TOF‐SIMS and corresponding 3D reconstruction images taken from cycled LRMO cathode in TFD. (g) The DMT modulus images of LRMO cathodes cycled in ED and TFD electrolyte. (h) Corresponding DMT modulus distribution of (g).

Since all LRMO||Li cells employ the same LRMO cathode, their intrinsic bulk redox chemistry should remain essentially identical. The electrolyte‑dependent variations in Mn/Ni redox reversibility therefore most likely originate from differences in interfacial stability and the resulting interface‑induced degradations to the bulk structure. This consideration highlights the CEI as a central regulator of capacity decay and motivates a detailed investigation into its formation kinetics, chemical evolution, and mechanistic function. Although LRMO||TFD||Li cells exhibit excellent electrochemical performance with markedly reduced capacity decay, the mechanistic origins of their superior interfacial stability remain insufficiently resolved. To track CEI evolution during the initial activation process, in situ galvanostatic EIS (IS‐GEIS) was performed at equal capacity intervals of 20 mAh g^−1^ (Figure 4a,b). TFD effectively suppresses structural changes associated with Li_2_MnO_3_ activation and mitigates side reactions such as electrolyte oxidation caused by lattice oxygen release. These effects collectively promote the formation of a uniform, stable CEI that passivates the LRMO surface. DRT analysis further support this conclusion (Note 4, Supporting Information), which reveals consistently lower R_ct_ and Z_w_ values (Figure 4c; Figures S19 and S20). IS‐GEIS measurements conducted during the 30th cycle further confirm that the CEI induced by TFD remains stable (Figures S21–S23 and Note S5), showing R_CEI_, R_ct_, and Z_w_ values throughout extended cycling.

Given that CEI formation is closely governed by the local solvation environment, the Li^+^ coordination dynamics during CEI formation were further probed by in situ Raman spectra (Figure 4d). Consistent with static Raman fitting, real‐time evolution of CIPs and AGGs was observed in TFD, accompanied by weak coordination/dissociation behavior of FEC and DFEA (Figure S24 and Note S6). In contrast, ED consistently exhibited strong Li^+^‐EC coordination and a stable SSIP‐dominated solvation structure. This dynamic evolution of CIPs and AGGs in TFD is critical, as it promotes a unique solvation structure that exposes anions to the electrode surface, thereby facilitating their preferential decomposition. This accelerated decomposition pathway directly contributes to the rapid and robust formation of a CEI enriched in inorganic components, such as LiF and Li─B─O species, which were subsequently identified by XPS. Consequently, TFD's ability to modulate Li^+^ coordination dynamics and induce the formation of a robust, inorganic‐rich CEI is a key factor in enhancing interfacial stability and Mn/Ni redox reversibility.

To verify these mechanistic insights at the structural level, the morphology and composition of the CEI were then analyzed. Transmission Electron Microscopy (TEM) provided direct visualization of the CEI's physical morphology and thickness. TEM analysis unequivocally demonstrated a remarkably thin and uniform CEI, measuring only 3.7 nm, on the surface of LRMO cycled in TFD. In stark contrast, LRMO cycled in ED developed a significantly thicker and heterogeneous CEI of 17.2 nm (Figure S25). This pronounced difference in CEI architecture underscores the superior passivation achieved with TFD. X‐ray photoelectron spectroscopy (XPS) depth profiling confirmed that cycling in TFD produces an inorganic hybrid CEI enriched in LiF and Li─B─O species (Figure 4e; Note S7). Such a composition provides both ionic conductivity and interfacial stability, effectively blocking direct contact between electrolyte and the highly reactive LRMO's surface, thereby suppressing the continuous decomposition of LiPF_6_ (Figures S26–S28). Time‐of‐flight secondary ion mass spectrometry (TOF‐SIMS) corroborated these findings, revealing higher concentrations of LiF and LiBO_2_, alongside significantly fewer LiPF_6_ decomposition products on LRMO surfaces cycled in TFD compared to ED (Figure 4f; Figure S29). This observation is crucial because the decomposition of LiPF_6_ inevitably generates highly corrosive HF. Therefore, the reduced presence of LiPF_6_ decomposition products in TFD is critical for preventing corrosion of the LRMO cathode surface, mitigating TM ion dissolution, and maintaining structural integrity.

Thereafter, the mechanical stability of the CEI was assessed using atomic force microscopy (AFM). The CEI formed in TFD exhibited a significantly higher Derjaguin–Muller–Toporov (DMT) modulus of 2.21 GPa, compared with 1.65 GPa in ED (Figure 4g,h). Energy dissipation values were also markedly lower in TFD (1.67 keV) than in ED (6.09 keV), confirming enhanced mechanical robustness (Figure S30). Specifically, the higher DMT modulus indicates that the TFD‐derived CEI possesses greater rigidity and resilience, enabling it to withstand the significant volumetric changes and mechanical stresses experienced by the cathode during repeated charge–discharge cycles without cracking or delamination. Concurrently, the lower energy dissipation values signify a more elastic and less dissipative interface, further ensuring the structural integrity and sustained passivation effect of the CEI over extended cycling. Taken together, these results demonstrate that TFD induces the rapid formation of a uniform, mechanically stable, and LiF/LiBO_2_ rich inorganic hybrid CEI. Such a CEI not only overcomes the mechanical fragility associated with ED but also effectively suppresses HF formation, thereby creating favorable conditions for inhibiting TM dissolution and ultimately mitigating interfacial degradation and capacity decay of LRMO cathodes.

### 2.5 Structural Evolution Against Jahn‐Teller Effect

**FIGURE 5:**
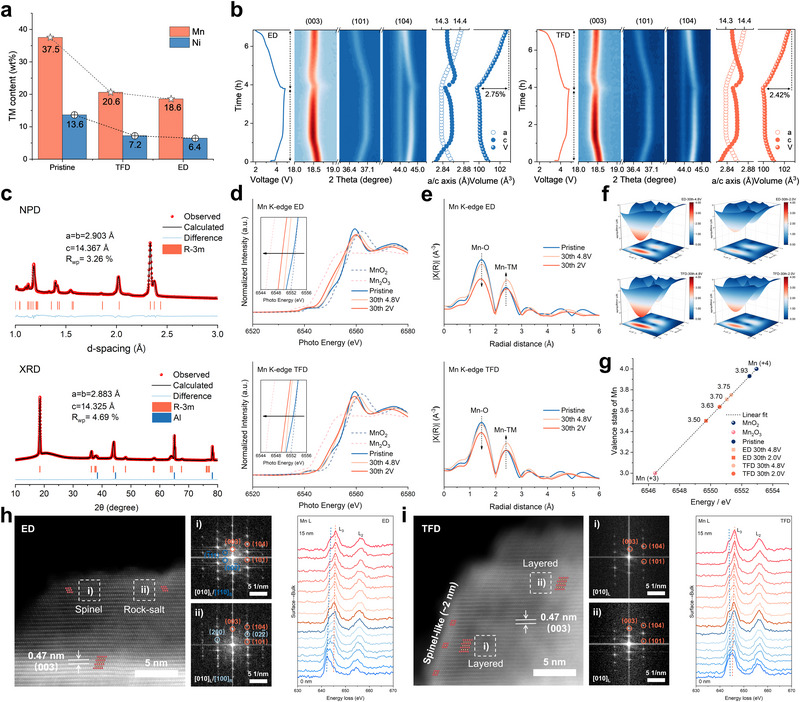
(a) ICP‐OES analysis of TM content from the cycled LRMO cathode. (b) Contour plots of in situ XRD results and the evolution of lattice parameters of LRMO cathode cycled in ED and TFD. (c) NPD and XRD Rietveld refinement of LRMO cathode cycled with TFD. (d) Mn K‐edge XANES of LRMO cathode cycled with ED and TFD under various operating conditions. (e) FT‐EXAFS spectra of Mn K‐edge XANES. (f) WT fitting results of Mn K‐edge EXAFS spectra at different conditions of LRMO cathode cycled with ED and TFD. (g) Calculated valence states of Mn from Mn K‐edge XANES data. HAADF‐STEM images and EELS spectra of Mn L‐edge from the surface to the inner bulk for LRMO cathode cycled in (h) ED, and (i) TFD.

The structural stability of LRMO cathodes, a critical determinant of cycling performance, was evaluated after cycling in ED and TFD electrolytes. Scanning electron microscopy (SEM) revealed significant structural degradation in cathodes cycled with ED. Specifically, ED‐cycled electrodes showed pronounced volumetric expansion, with thickness increasing to 25.2 µm, considerably larger than the 15.3 µm observed in TFD (Figure S31). Plan‐view images further revealed severe particle fracture in ED (Figure S32), a phenomenon consistent with substantial internal stress and continuous phase transitions reported for LRMO cathodes during cycling, leading to pulverization and capacity decay [44]. Consistent with this, inductively coupled plasma optical emission spectroscopy (ICP‐OES) results demonstrates continuous Mn and Ni dissolution of LRMO cathode during long‐term cycling in ED (Figure 5a), whereas TFD effectively suppresses TM ions dissolution in LRMO cathodes. Furthermore, TOF‐SIMS analyses confirmed this point (Figure S33), showing that LRMO cathodes cycled in TFD retained higher TM content, indicative of reduced dissolution. These observations highlight the critical role of CEI stability in preventing structural degradation. Further insights into structural stability were gained from in situ X‐ray diffraction (XRD) (Figure 5b). The results show that when TFD is employed as the electrolyte, variations in lattice parameters (a, c) and unit‐cell volume are significantly reduced, with a maximum volume change of 2.42%, smaller than that of 2.75% while ED is used. This smaller volumetric fluctuation in TFD is indicative of suppressed crystallographic distortion, specifically linked to the mitigation of J‐T effects caused by Mn^3+^ generation. Ex situ Rietveld refinement results of neutron powder diffraction (NPD) and XRD corroborated well with the in situ XRD results, confirming that LRMO cathodes cycled in TFD undergo smaller volume changes after long‐term cycling (Figure 5c; Figures S34–S36 and Note S8). This preservation of structural integrity and suppressed volumetric changes are fundamentally attributed to the mitigation of J‐T distortions.

As evidenced by the dQ/dV curves (Figure 3e), the fundamental divergence in the capacity decay mechanisms of the LRMO cathode in ED and TFD electrolytes is attribute to J‐T distortions arising from high‑spin Mn^3+^ species. To further elucidate the local structural evolution of LRMO cathodes cycled in ED and TFD electrolytes under different operating conditions, X‐ray absorption near‐edge structure (XANES) and extended X‐ray absorption fine structure (EXAFS) measurements were conducted for Mn and Ni (Figure 5d,e; Figure S37). For LRMO cathodes cycled in ED, the Mn XANES spectra exhibited a pronounced shift toward lower energies, indicating a substantial reduction in Mn oxidation states. In contrast, the Mn XANES spectra of LRMO cathodes cycled in TFD remained closer to that of the pristine LRMO, suggesting more reversible Mn redox processes. Furthermore, the Fourier‐transformed (FT) EXAFS peaks of Mn‐O coordination shells remained relatively stable in TFD, consistent with the wavelet transform (WT) fitting results (Figure 5f; Figure S38), which collectively reveal enhanced Mn redox reversibility in TFD. More importantly, valence states extracted from XANES data demonstrate that Mn in LRMO cycled with TFD consistently maintains higher oxidation states compared with ED (Figure 5g). This Mn instability further propagates to Ni, undermining the layered structure (Note 9, Supporting Information). The improved Mn redox reversibility observed in TFD, supported by both XANES and dQ/dV analyses, highlights the critical role of suppressing J‐T distortions associated with Mn^3+^.

In order to validate above results, aberration‐corrected scanning transmission electron microscopy (AC‐STEM) and electron energy‐loss spectroscopy (EELS) analyses were conducted to probe the local structural evolution and Mn valence states in LRMO cathodes cycled with ED and TFD. High‐angle annular dark‐field (HAADF) STEM and fast Fourier transform (FFT) images reveal that in ED, extensive surface degradation occurs, forming spinel and inert rock‐salt phases (Figure 5h), whereas in TFD only a thin (∼2 nm) spinel layer is observed (Figure 5i). Importantly, depth‐resolved EELS spectra of the Mn L‐edge provide a continuous, quantitative profile of the Mn valence and spin‐state characteristics from the surface to inner bulk (0–15 nm). In the ED‐cycled LRMO cathode, the Mn L_3_/L_2_ ratio at the extreme surface is as high as 1.92, and it remains above 1.60 even at a depth of 5 nm. This pronounced low‐energy shift and large L_3_/L_2_ ratio indicate a high concentration of unstable high‐spin Mn^3+^ species and substantial Mn valence reduction. In sharp contrast, the TFD‐cycled LRMO cathode exhibits a much lower surface L_3_/L_2_ ratio of 1.71, which rapidly stabilizes to a lower value of approx. 1.52 beyond 4 nm. This consistently lower L_3_/L_2_ ratio and the higher energy position of the L_3_ peak confirm that Mn ions retain a stable, high‐valence, low‐spin‐like Mn^4+^ state throughout the bulk continuum. These results demonstrate that the reduction of Mn^4+^ to high‐spin Mn^3+^ is fundamentally suppressed not just at the surface but within the inner lattice, thereby effectively mitigating J‐T distortions. Additionally, analysis of the O K‐edge spectra reveals that in ED, the pre‐edge peak at 530 eV progressively decreases from the surface to the bulk, nearly disappearing within the top 2 nm, indicating significant oxygen loss (Figure S39). Conversely, in LRMO cycled with TFD, the O K‐edge pre‐peak remains pronounced from the surface through the 15 nm bulk region. In summary, the high‐resolution STEM‐EELS data demonstrate that the TFD electrolyte effectively stabilizes the Mn electronic structure at the immediate interface and, more critically, intercepts the detrimental chain reaction of valence/spin‐state degradation from the surface to the bulk. By suppressing this cross‐region propagation of electronic instability, the TFD fundamentally mitigates Mn‐induced Jahn‐Teller distortions across the surface‐to‐bulk continuum, ensuring the extreme structural integrity and negligible capacity decay of the LRMO cathode.

As mentioned above, the stable CEI formed in TFD demonstrably enhances the structural integrity and cycling performance of LRMO cathodes. By effectively preserving Mn's high valence state and maintaining oxygen lattice stability at the atomic scale, TFD successfully suppresses J‐T distortions and mitigates detrimental surface phase transitions. This comprehensive stabilization strategy from atomic‐to‐macroscopic scale ultimately preserves the crystal structure of LRMO cathode and particle integrity, leading to superior long‐term cycling stability.

### 2.6 Theoretical Analysis of Capacity Decay Mechanisms

**FIGURE 6:**
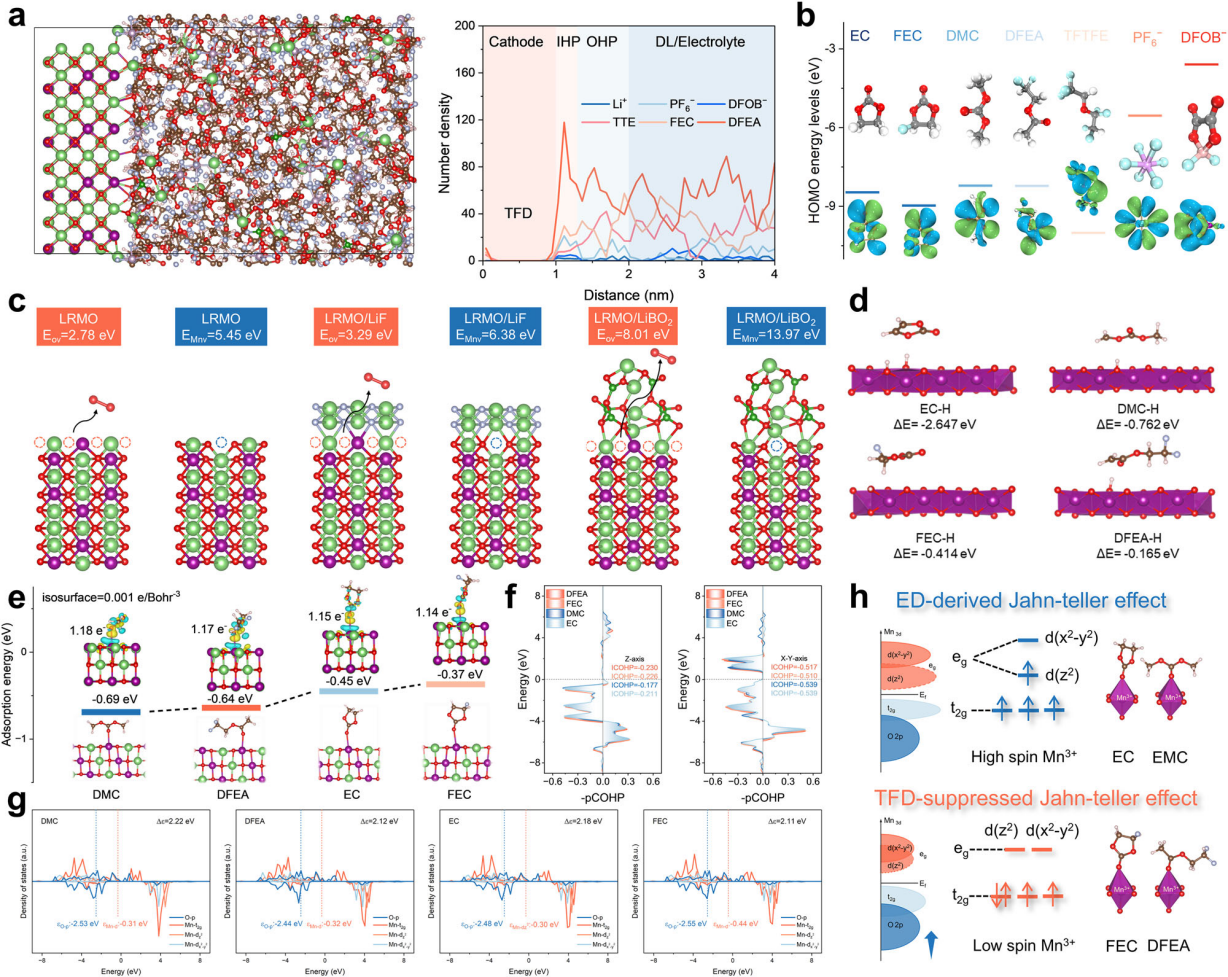
(a) Snapshot and corresponding EDL distribution at the Li_2_MnO_3_/TFD interface extracted from MD simulation. (b) Calculated HOMO energy levels of electrolyte components. (c) The formation energy of oxygen vacancy and Mn vacancy in LRMO, LRMO/LiF, and LRMO/LiBO_2_ interface. (d) Computational results of H‐transfer energies of DMC, DFEA, EC and FEC on the surface of highly delithiated LRMO surface. (e) Adsorption energies of different solvent molecules on the surface of LiMnO_2_. (f) Crystal orbital Hamilton population (COHP) analysis for Mn─O bond along Z‐axis and X‐Y‐axis. (g) The PDOS plots of Mn d and O p orbitals. (h) Schematic of orbital energy diagram mediated by fluorinated molecules to suppress Jahn‐Teller effect.

The above results collectively demonstrate that mesoscopic regulation of the CEI and suppression of J‐T distortions stabilize the LRMO cathode and mitigate capacity decay. To gain deeper insight into the regulation and formation mechanism of the CEI, interfacial MD simulations were performed to reveal the distribution of species within the electrical double layer. The simulations showed that both PF_6_^−^ and DFOB^−^ anions can extensively penetrate into the inner Helmholtz plane (IHP) of LRMO/TFD interface (Figure 6a), whereas in the LRMO/ED interface (Figure S40), only EC and DMC molecules were observed within the IHP. To further determine the sequence of controlled decomposition leading to CEI formation, the highest occupied molecular orbital (HOMO) energy levels of electrolyte components were calculated. The results indicate that in the TFD electrolyte, DFOB^−^ preferentially decomposes to form a CEI enriched in LiF and LiBO_2_, thereby protecting TFD from excessive decomposition. In contrast, in the ED electrolyte, PF_6_^−^ undergoes significant decomposition and generates subsequent harmful byproducts. (Figure 6b). These findings suggest that TFD induces the formation of a LiF/LiBO_2_‐rich inorganic hybrid CEI within the IHP, consistent with XPS and TOF‐SIMS results.

To elucidate the stabilizing effect toward LRMO structure of the formed CEI, Figure 6c presents the calculated formation energies of oxygen and manganese vacancies at the LRMO, LRMO/LiF, and LRMO/LiBO_2_ interfaces. The formation energies of Mn and O vacancies in LiBO_2_ are the highest (13.97 and 8.01 eV, respectively). At the LRMO/LiF interface, these values (6.38 eV and 3.29 eV) are also higher than those in pristine LRMO (5.45 and 2.78 eV). Furthermore, the dehydrogenation transfer energies of solvents on the fully delithiated LRMO surface revealed that EC (−2.65 eV) and DMC (−0.76 eV) exhibit much higher values compared with the three solvents in TFD, indicating that ED is more prone to decomposition on the LRMO surface, producing harmful organic by‐products (Figure 6d; Figure S41). These combined theoretical insights, from MD simulations and DFT calculations, confirm that TFD induces the formation of an inorganic CEI enriched in LiF and LiBO_2_, which effectively stabilizes the LRMO structure and minimizes the generation of undesirable interfacial organic by‐products. This regulation of species distribution within the IHP thus enables controlled and sequential decomposition, leading to the construction of a uniform and robust mesoscopic CEI.

As for the electrolyte‐mediated suppression of the J‐T effect in ED and TFD electrolytes along LRMO cathode interface, DFT calculations were performed using LiMnO_2_ as a model system for solvent adsorption (Figure 6e). This choice is based on the fact that Mn^4+^ has no electrons in the e_g_ orbital and thus does not undergo J‐T distortion, whereas the effect is primarily associated with the orbital degeneracy of high‐spin Mn^3+^, leading to axial Mn‐O bond elongation and equatorial Mn‐O bond compression. Adsorption energy calculations revealed that DFEA and FEC exhibit adsorption energies of −0.64 and −0.37 eV, respectively, which are lower than those of DMC (−0.69 eV) and EC (−0.45 eV). This difference arises from the strong electronegativity of fluorine atoms in fluorinated molecules, which reduces the electron density at carbonyl oxygen coordination sites, thereby weakening the interaction between solvent molecules and the LRMO surface and suppressing coupled decomposition reactions.

Crystal orbital Hamilton population (COHP) analysis showed that the coordination of DFEA and FEC with Mn atoms significantly modulates the J‐T effect: axial Mn─O bonds are strengthened, while equatorial Mn─O bonds are weakened, corresponding to suppression of J‐T distortion (Figure 6f). Projected density of states (PDOS) analysis further revealed an increased band center separation between Mn d_z2_ orbitals and O p orbitals, confirming the orbital regulation effect of TFD (Figure 6g). Based on these findings, we propose a schematic mechanism in which fluorine atoms in solvent molecules reduce the electron density at double‐bonded oxygen sites, resulting in weaker coordination with Mn and driving a spin‐state transition from high‐spin to low‐spin, thereby fundamentally alleviating the J‐T effect (Figure 6h).

This molecular‐level orbital regulation at the interface serves as the foundation for achieving surface‐to‐bulk structural stabilization, which is governed by the source‐quenching effect of J‐T distortion that effectively intercepts the structural degradation chain. Specifically, when LRMO cathode undergoes charging/discharging process in the conventional ED electrolyte, structural failure is primarily triggered by the localized J‐T distortion at the surface. This distortion destabilizes the surface lattice, which further initiates progressive oxygen loss and an irreversible phase transition sequence from layered to spinel and eventually to inert rock‐salt phases. As evidenced by AC‐STEM and EELS results, the localized J‐T distortion and resultant micro‐stress at the surface act as a trigger, inducing micro‐cracks that propagate toward the inner bulk and ultimately lead to a domino‐like structural collapse. In contrast, the orbital regulation of Mn atoms at the interface by fluorinated molecules in the TFD electrolyte maintains a stable low‐spin configuration, effectively inhibiting the occurrence of J‐T distortion and successfully blocking the initial trigger of lattice distortion. This interfacial stabilization prevents the inward propagation of stress and structural damage, thereby ensuring that the layered structure from the surface to the inner bulk is maintained. Consequently, this surface‐initiated stabilization mechanism enabled by interfacial orbital regulation provides a comprehensive protection strategy, stabilizing the inner lattice through precise orbital modulation of Mn at surface.

## 3 Conclusions

In summary, this work establishes a unified mechanistic picture of how molecule‐induced electrolytes can fundamentally mitigate capacity decay in Li‐rich Mn‐based oxides. Through complementary structural and interfacial analyses, we reveal that the exceptional stability arises from the synergistic action of two molecularly driven processes: the in situ construction of a thin, uniform, and highly inorganic LiF/LiBO_2_‐rich CEI, and the fluorinated‐molecule‐induced modulation of Mn spin states that suppresses Jahn‐Teller distortion at its origin. The hybrid CEI effectively passivates surface oxygen species and blocks parasitic reactions, while the spin‐state regulation alleviates bulk lattice instability and prevents long‐term structural degradation. Theoretical calculations further validate that these interfacial and bulk regulations are intrinsically coupled, jointly lowering the energetic pathways associated with degradation. This electrolyte‐induced dual‐regulation strategy provides a generalizable electrolyte‐design principle for stabilizing Li‐rich cathodes and offers a promising route toward high‐energy‐density lithium‐ion batteries with negligible capacity decay.

## Conflicts of Interest

The authors declare no competing interests.

## Data availability

The data are available from the corresponding author upon reasonable request. Source data are provided in this paper.

## Supporting information




**Supporting File**: adma72969‐sup‐0001‐SuppMat.docx.
