# Supplementary material for: Electrolyte‐Induced Interfacial/Bulk Dual Regulation Enables Negligible Capacity Decay in Li‐Rich Cathodes
Source: Adv Mater. 2026 Apr 6;38(26):e72969. doi: 10.1002/adma.72969 (PMC13155261; doi:10.1002/adma.72969)
Supplement: Supplementary file 1 — Supporting File: adma72969‐sup‐0001‐SuppMat.docx. [file ADMA-38-e72969-s001.docx]

**Supporting Information**

**Electrolyte‑Induced Interfacial/Bulk Dual Regulation Enables Negligible Capacity Decay in Li‑Rich Cathodes**

Tianqi Yang, Min Jiang, Jiatao Lou, Zhouyu Huang, Xingjun Li, Liuqi Wang, Qingru Zhou, Lun Li, Liuyi Hu, Wei Liu, Yuzhi He, Xingyu Wang, Zhengbo Liu, Wenkui Zhang, Jun Zhang, Xinhui Xia, Yang Ren, Qi Liu*

Dr. T. Yang, Dr. M. Jiang, X. Li, L. Wang, W. Liu, Y. He, X. Wang, Dr. Z. Liu, Prof. Y. Ren, Prof. Q. Liu

Department of Physics, City University of Hong Kong, Hong Kong 999077, China.

E-mail: qiliu63@cityu.edu.hk

J. Lou

Tsinghua Shenzhen International Graduate School, Tsinghua University, Shenzhen 518057, China.

Z. Huang, Q. Zhou, Prof. W. Zhang, Prof. J. Zhang, Prof. X. Xia

College of Materials Science and Engineering, Zhejiang University of Technology, Hangzhou 310014, China.

L. Li

China-UK Low Carbon College, Shanghai Jiao Tong University, Shanghai 200240, P.R. China.

L. Hu

State Key Laboratory of Chemical Engineering, Department of Chemistry, Zhejiang University, Hangzhou 310027, China

Prof. Y. Ren, Prof. Q. Liu

Shenzhen Research Institute, City University of Hong Kong, Shenzhen 518057, China.

Prof. Y. Ren,

Department of Physics, JCSTEM Lab of Energy and Materials Physics, City University of Hong Kong, Hong Kong 999077, China.

**Keywords:** Capacity decay, interfacial degradation, Jahn-Teller distortion, Li-rich cathode, electrolyte-induced

**I. Supporting Text**

1. **Experimental Section**
   1. *Preparation of electrolytes*

LiPF_6_ (99.9%, battery grade, DoDoChem), LiDFOB (99.9%, battery grade, DoDoChem), TFTFE (99.8%, battery grade, DoDoChem), FEC (99.9%, battery grade, DoDoChem), and DFEA (99.8%, battery grade, DoDoChem) were used without purification. The ED electrolyte was obtained by dissolving 1.2 M LiPF_6_ in EC/DMC (3:7 vol%) (DoDoChem). The TFD electrolyte was obtained by dissolving 1 M LiPF_6_ and 0.2 M LiDFOB in the mixture (TFTFE/FEC/DFEA, 1:1:2 by volume). All procedures were executed in an argon-filled glove box (H_2_O, O_2_ < 0.01 ppm).

- 1. *Electrode Preparation*

The LRMO material used in this work was purchased from Shenzhen Sufang New Energy Technology Co., Ltd.. To assemble a full battery for testing, the cathode material was fabricated by dissolving LRMO, PVDF, and SP into NMP at a mass ratio of 8:1:1, then the slurry was coated onto an aluminum foil and dried at 80 ^o^C in a vacuum oven for overnight. The mass loading of LRMO in the cathode was 2.5-3 mg cm^−2^. CR2032-type coin batteries were fabricated in an argon-filled glove box (H_2_O, O_2_ < 0.01 ppm). Coin cell cases, springs, and spacers were supplied by Canrd Technology Co. Ltd. The charge/discharge tests of the batteries were performed between 2 V and 4.8 V on a battery testing system (NEWARE, CT-4008Tn-5V50mA-HWX, Shenzhen, China). For the pouch cell assembly, the LRMO cathode slurry was prepared by dispersing the active material, composite conductive agents (Super-P and ETP-608), and a hybrid binder system (LB107-44 and PVDF) in N-methyl-2-pyrrolidone (NMP) with a mass ratio of 94:3:0.1:0.4:2.5. Simultaneously, the graphite anode slurry was formulated by mixing Graphite (F50), Super-P, and an aqueous binder system comprising CMC (MAC500LC) and SBR (BM-430B) in deionized water with a mass ratio of 94.3:3.0:1.2:1.5. The respective slurries were coated onto current collectors (Al foil for the cathode and Cu foil for the anode). Specifically, the LRMO cathode featured a double-sided total mass loading of 26 mg/cm^2^, while the graphite anode was prepared with a double-sided loading of 12.6 mg/cm^2^. The corresponding N/P ratio (negative/positive capacity ratio) was maintained within a safety window of 1.1-1.2. The Li-metal foil (17 μm, 99.95%) used in reference cells was a high-uniformity commercial product purchased from China Energy Lithium Co., Ltd..

- 1. *Materials characterization*

^7^Li NMR spectra were obtained using a Bruker Avance III HD 500 NMR spectrometer with acetonitrile-d3 as the deuterated solvent. The Raman spectra were collected using a DXR Raman microscope (Renishaw InVia Raman spectrometer) with He-Ne 532 nm laser excitation, the in situ Raman test were carried out on the same machine with a specially designed battery device. The differential scanning calorimetry (DSC) tests were conducted using Netzsch STA 449 F5 with a scanning speed of 10 ^o^C/min. The 4.8V charged cathodes were washed with dimethyl carbonate to remove residual electrolyte and dried in a glovebox. For DSC tests, the charged cathode powders were scraped off and mixed with the corresponding electrolyte with the ratio set as 1 μL/mg. Specifically, the charged LRMO cathode charged using ED is further mixed with ED, and the charged LRMO cathode charged using TFD is further mixed with TFD.XPS measurements were performed on a Thermo ESCALAB 250 system with a monochromatic Al-Kα (1486.6 eV) X-ray source to investigate the chemical state of elements. TOF-SIMS was applied to accurately reveal the chemical composition of the surface of the LRMO cathode via TOF.SIMS 5-100 instrument (IONTOF GmbH, Germany). A pulsed 60 keV Bi^3+^ ion beam was set, and the selected analysis area was 70 × 70 um. AFM (Bruker Dimension ICON) was conducted on the cycled LRMO cathode to evaluate the mechanical properties of CEI. The morphology of samples was observed through a field emission scanning electron microscope (FESEM, HITACHI Regulus 8100) at an acceleration voltage of 15 kV. The actual atomic ratios in cycled LRMO cathode were determined by an Agilent 720ES ICP-OES according to standard profiles. The XRD patterns of cycled LRMO cathode samples were collected by the Rigaku Smartlab X-ray Diffractometer (Japan, Cu Kα1, λ=1.54186 Å). The in situ XRD experiments were carried out on the same machine with a specially designed battery device. The Rietveld refinement of the XRD patterns and NPD results was carried out using the GSAS-2 program. X-ray absorption fine structure (XAFS) spectroscopy was carried out using the *Rapid* XAFS 2M (Anhui Absorption Spectroscopy Analysis Instrument Co., Ltd.) by transmission mode at 20 kV and 20 mA. The X-ray absorption near-edge structure (XANES) and extended X-ray absorption fine structure (EXAFS) data were processed using Athena and Artemis programs in Demeter software. High-resolution transmission electron microscope (HRTEM) images and atomic-resolution high-angle annular dark-field scanning transmission electron microscope (HAADF-STEM) images were performed on a spherical aberration-corrected STEM instrument (ThermoFisher Spectra 300, USA), equipped with a 5th order aberration corrector. The electron energy loss spectra (EELS) were used to analyze the element valence states of cycled LRMO cathode (ThermoFischer Spectra 300, USA).

- 1. *Electrochemical characterization*

The ionic conductivity of electrolytes from 30 °C to 70 °C was measured using stainless steel (SS) symmetric cells, with impedance recorded over a frequency range of 100 mHz to 1 MHz on a Zahner Zennium electrochemical workstation. The ionic conductivity ($\sigma$) values was determined by the following equation:

$$\sigma=\frac{D}{R\cdot S}$$

where D is the thickness of the CPEs, R is the resistance value of CPEs obtained by the EIS test, and S is the area of the SS. Linear sweep voltammetry (LSV) was conducted from 2 V to 6 V at a scan rate of 1 mV s^−1^ by Ivium-n-Stat.

The lithium-ion transfer number ($t_{Li}^{+}$) was calculated by the following equation:

$$t_{Li}^{+}=\frac{I_{s}\left( \Delta_{v}-I_{0}R_{0} \right)}{I_{0}\left( \Delta v-I_{s}R_{s} \right)}$$

where ΔV is the polarization voltage (10 mV), R_0_ and I_0_ are the resistance and current before polarization, R_s_ and I_s_ are the resistance and current after polarization, respectively.

In situ galvanostatic electrochemical impedance spectra (IS-GEIS) were performed with the LRMO||Li cells in the range from 2 V to 4.8 V at 0.5 C, which recorded EIS with an equidistant capacity of 20 mA h g^−1^. The distribution of relaxation time (DRT) analysis was performed using DRT tools to resolve characteristic relaxation processes of EIS. The 4.75 V LRMO||graphite cell was activated within using a meticulously designed stepwise current formation protocol. This procedure was specifically implemented to decouple the initial formation of the solid electrolyte interphase on the graphite anode from the high-voltage activation of the Li_2_MnO_3_ phase in the LRMO cathode. During the formation stage, a low current density of 0.02 C was applied from the open-circuit voltage (OCV) to 3.3 V, followed by 0.05 C from 3.3 V to 4.4 V. This targeted approach ensures the development of a high-quality and robust SEI layer on the graphite surface before the aggressive Li_2_MnO_3_ phase activation. Subsequently, the cathode activation was completed between 4.4 V and 4.75 V using a CC/CV (0.1 C / 0.02 C) charging method, followed by a discharge rate at 0.1 C.

- 1. *Molecular dynamics simulations*

Molecular dynamics (MD) simulations were performed using the Gromacs^[1]^ program suite with the General Amber Force Field generated by Sobtop. The systems are set up initially by using the packmol^[2]^ program in a periodic cubic box with a length of 50 Å. Van der Waals forces and the electrostatic interactions were treated using the Particle-mesh Ewald method^[3]^. First, NVT simulations were performed at 298.15 K for 1 ns with a Velocity-rescale^[4]^ thermostat after energy minimizing calculation. After that, NPT simulations run for 1 ns under the control of Berendsen's^[5]^ barostat to balance the pressure^46^. Finally, the MD simulation was conducted for a total simulation time of 10 ns.

- 1. *DFT calculations*

DFT calculations of frontline orbital were performed using the Gaussian 16 program package.^[6]^ The geometry of molecules and the vibrational modes were optimized at the B3LYP^[7]^ level with the 6-311++G(d,p) basis set^[8]^. To investigate the role of the electrolyte environment, the SMD implicit solvation model were used with acetone to represent the solvent for calculations. The definition of binding energy (G_bind_) is as follows:

$$G_{bind=}{G_{\mathrm{Total}}{-G}_{\mathrm{Li}^{+}}-G}_{\mathrm{Mole}}$$

where G_Total_, G_Li_^+^, and G_Mole_ are the Gibbs free energies of the whole model, Li atom, and molecules, respectively. The visualizations of HOMO, LUMO and ESP were achieved using Multiwfn^[9]^ and VMD^[10]^. Other DFT calculations were carried out using the Vienna ab initio simulation package (VASP)^[11]^ with plane wave basis sets and projector-augmented wave (PAW)^[12]^ pseudopotentials. Perdew-Burke-Ernzerhof (PBE) generalized-gradient approximation (GGA) was adopted to describe the exchange-correlation functionals.^[13]^ The energy cutoff of 520 eV was used for all calculations, and the convergence criterion for energy and force for structural relaxation were set as 1.0×10^−5^ eV and 0.02 eV Å^−1^, respectively. We employ a Hubbard-U term to treat the strongly correlated of the d-electrons of Mn atoms. and the value was adopted as 5.2 eV for Mn. The definition of adsorption energy (G_bind_) is as follows:

$$E_{ads=}{E_{\mathrm{Total}}{-E}_{\mathrm{slab}}-E}_{\mathrm{Mole}}$$

where E_Total_, E_slab_, and E_Mole_ are the energies of the whole model, slab model, and adsorbed molecules, respectively. Data analysis and visualization for all structures in VASP calculations was achieved with VASPKIT and VESTA, respectively.

1. **Supplementary Notes**
   1. *Supplementary Note 1*

As illustrated in **Figure 1g** and **Figure S3b** (**Supporting Information**), the binding energies of Li^+^ with ethylene carbonate (EC), dimethyl carbonate (DMC), fluoroethylene carbonate (FEC), di(2,2,2-trifluoroethyl) adipate (DFEA), and trifluoroethyl trifluoroethyl ether (TFTFE) are −0.78 eV, −0.76 eV, −0.68 eV, −0.71 eV, and −0.38 eV, respectively. The stronger binding observed for EC and DMC arises from the high electron density of carbonyl oxygen atoms, which provide robust ion-dipole interactions with Li^+^. In contrast, fluorination reduces the electron‑donating ability of coordinating sites due to the strong electronegativity of fluorine, thereby lowering the Lewis basicity of FEC, DFEA, and TFTFE. This electronic withdrawal effect, combined with steric hindrance from bulky fluorinated substituents, accounts for the reduced binding energies. Meanwhile, upon coordination with PF_6_^−^ and DFOB^−^, the increase in dipole moment for Li⁺-fluorinated solvent complexes is significantly smaller, indicating suppressed polarization response and donor capability (**Figure S4**, **Supporting Information**). The molecular models further illustrate the coordination structures of Li^+^ solvates formed with EC, DMC, FEC, DFEA, and TFTFE. Li⁺-DMC (1.798 Å) and Li⁺-EC (1.814 Å) exhibit the shortest bond lengths, reflecting strong coordination with conventional carbonates. Li⁺-DFEA (1.815 Å) and Li⁺-FEC (1.834 Å) show slightly longer distances, suggesting moderately weakened coordination due to fluorination. Li⁺-TFTFE (2.075 Å) presents the longest bond, indicating minimal coordination and functioning primarily as a diluent. In summary, both binding energy and bond length analyses consistently demonstrate that fluorinated and ether‑based solvents coordinate more weakly with Li⁺ than non‑fluorinated carbonates. This weak solvation facilitates the formation of anion‑rich CEIs and supports high‑rate performance in LRMO||Li cells.

- 1. *Supplementary Note. 2*

The Raman spectra and radial distribution function (RDF) plots reveal distinct Li⁺ solvation environments in TFD and ED electrolytes, governed by differences in solvent composition and coordination behavior. In ED (**Figure S7a**, **Supporting Information**), Raman peaks at ~717 cm^−1^, ~730 cm^−1^, and ~742 cm^−1^ correspond to free EC, coordinated EC, and solvent‑separated ion pairs (SSIPs), with the dominant coordinated EC peak indicating strong Li⁺–solvent interactions and a tightly bound solvation shell. In contrast, TFD (**Figure 1h**) exhibits a broader distribution of species, including free FEC (~722 cm^−1^), coordinated FEC (~728 cm^−1^), SSIPs (~735 cm^−1^), contact ion pairs (CIPs, ~745 cm^−1^), and aggregates (AGGs, ~752 cm⁻¹). The presence of CIPs and AGGs reflects weakened solvent coordination and enhanced ion pairing, characteristic of a low‑polarity, weakly solvating environment. RDF analysis corroborates these observations. In ED (**Figure S7b**, **Supporting Information**), Li⁺ shows strong coordination with EC and DMC oxygen atoms, as evidenced by sharp g(r) peaks and high coordination numbers, while interactions with F^−^ (from PF_6_^−^) remain weak. In TFD (**Figure S8**, **Supporting Information**), Li⁺-O interactions with FEC, DFEA, and TFTFE are significantly weaker, producing broader g(r) peaks and lower coordination numbers. Coordination number plots further reveal stronger Li⁺-PF_6_^−^ ion pairing in TFD, confirming that its weakened solvation shell facilitates closer cation–anion interactions and the formation of CIPs and AGGs. To assess kinetic constraints, desolvation energies were computed from MD snapshots using

$$E_{\text{des}}=E_{\text{Total}}-E_{A}-E_{B}$$

Where $E_{\mathrm{des}}$is the desolvation energy, $E_{\mathrm{Total}}$ is energy of solvated structure, $E_{A}$ and $E_{B}$ are energies of species in solvated structure and A, B can be Li^+^, EC, DMC, FEC, DFEA and PF_6_^−^. ED favors SSIP configurations with short Li⁺–O distances (~2.05 Å) and higher desolvation energy (2.437 eV), indicating a stable, tightly bound solvation shell. In contrast, TFD promotes CIP structures with longer Li⁺–O distances (2.18–2.31 Å) and lower desolvation energy (2.269 eV), suggesting that Li⁺ is more readily desolvated (**Figure S9**, **Supporting Information**). In summary, both spectroscopic and computational analyses consistently demonstrate that TFD forms a weakly solvated, anion‑rich environment. This reduced solvation stability enhances cation–anion association, thereby facilitating faster interfacial charge transfer and improved rate performance in LRMO||Li cells.

- 1. *Supplementary Note. 3*

To elucidate the origin of rate performance disparities, LRMO||LRMO symmetric cells were assembled and subjected to temperature-dependent electrochemical impedance spectra (EIS), followed by distribution of relaxation time (DRT) analysis to deconvolute the CEI interfacial resistance (R_CEI_), charge-transfer resistance (R_ct_), and Warburg diffusion impedance (Z_W_). DRT results reveal pronounced temperature-dependent relaxation features for both TFD and ED systems (**Figure 2c; Figure S14, Supporting Information**). Even at different temperatures in the range from 30^o^C to 70^o^C, the LRMO||TFD||LRMO cell exhibits significant smaller values of R_CEI_ and R_ct_, along with a reduced Z_W_ peak intensity, indicating enhanced ion transport and mitigated interfacial polarization. In contrast, the ED-based cell consistently displays broader Z_W_ features and relatively higher R_CEI_ across the entire temperature range, suggesting sluggish charge-transfer kinetics and limited Li^+^ diffusion efficiency. These differences in impedance response directly correlate with the observed rate performance: the TFD system maintains lower polarization and faster kinetics under high-rate conditions, whereas the ED system suffers from increased impedance accumulation, leading to compromised capacity retention at elevated current densities. Thus, the DRT analysis mechanistically attributes the superior rate capability of the TFD electrolyte to its more stable CEI formation and improved interfacial transport properties.

- 1. *Supplementary Note. 4*

In LRMO||Li cells, DRT results for *In-situ* GEIS results of the initial cycle reveal pronounced differences in impedance evolution between the TFD and ED electrolytes during the first charging and discharging processes. Three distinct relaxation mechanisms are identified: SEI interfacial resistance (R_SEI_), R_ct_, and Z_W_. TFD exhibits a higher R_SEI_, likely due to rapid passivation and formation of a LiF‑rich interphase. However, it consistently shows lower R_ct_ and Z_W_ throughout cycling, indicating that the CEI formed in TFD is compact and chemically stable, enabling efficient Li^+^ transport while suppressing surface reconstruction and polarization. In contrast, ED produces a thinner but less protective interphase, resulting in higher R_ct_, and Z_W_ from ongoing parasitic reactions and degraded transport pathways. Bode plot analysis further highlights these differences. During high‑voltage charging, ED induces a pronounced increase in mid‑frequency R_ct_ and low‑frequency Z_W_, accompanied by intensified negative phase shifts, reflecting irreversible resistance buildup and a transition toward film‑ and diffusion‑controlled processes. By comparison, TFD stabilizes the interface, showing only a minor rightward shift of the mid‑frequency peak and reduced polarization in the Z_W_ region. During discharge, ED continues to exhibit rising impedance and deepened phase shifts, consistent with irreversible degradation and accumulation of parasitic byproducts. TFD, however, maintains stable impedance, underscoring the robustness of its CEI in sustaining ion transport. In summary, although TFD incurs a higher initial R_SEI_, its stable CEI ensures lower R_ct_, and Z_W_, mitigates interfacial degradation, and preserves kinetic reversibility. Together, DRT and Bode analyses confirm that TFD delivers superior interfacial stability, reduced polarization, and enhanced rate capability under dynamic high‑voltage conditions.

- 1. *Supplementary Note. 5*

To evaluate the long‑term stability of CEIs formed by ED and TFD electrolytes, in‑situ GEIS measurements were conducted after 30 cycles (**Figure S21**, **Supporting Information**). The Nyquist plots reveal that ED induces a significant increase in interfacial impedance during charging, indicating aggravated degradation and hindered ion transport. In contrast, TFD shows only moderate impedance evolution across the voltage range, suggesting enhanced stability and suppressed polarization. This divergence persists during discharge: ED continues to exhibit rising impedance, implying irreversible degradation, whereas TFD maintains relatively stable impedance characteristics, reflecting the robustness of its CEI in sustaining ion transport. DRT analysis further substantiates these findings (**Figure S22**, **Supporting Information**). At the 30th cycle, the RSEI component in TFD is significantly reduced and remains stable, attributed to the progressive activation of the LiF‑rich SEI. This inorganic‑dominant interphase facilitates efficient Li^+^ transport while suppressing parasitic reactions, thereby minimizing SEI resistance. In contrast, ED shows a marked increase in RSEI during both charge and discharge, indicating aggravated anode degradation and limited cathodic reversibility. Moreover, TFD consistently maintains low‑intensity R_ct_ and Z_W_, reflecting preserved interfacial integrity and sustained ion transport. In summary, both GEIS and DRT analyses confirm that the CEI formed in TFD undergoes favorable maturation during cycling, leading to reduced interfacial resistance, improved Li⁺ transport kinetics, and effective mitigation of capacity decay in LRMO cathodes.

- 1. *Supplementary Note. 6*

During the initial charging/discharging process of LRMO||TFD||Li cell, the *in-situ* Raman contour (570 cm^−1^) directly captures the vibrational response of DFEA (**Figure S24**, **Supporting Information**). A rapid decrease in peak intensity and slight spectral shift during charging indicate a reduced involvement of DFEA in the primary Li^+^ solvation shell. This attenuation suggests weakened Li^+^-DFEA coordination and a local transition toward an anion-enriched interfacial environment. The partial hysteresis of the DFEA peak during discharge implies that the coordination change is not fully reversible, consistent with the onset of interfacial reconstruction. Mechanistically, the fast decline in DFEA peak intensity reflects the dynamics of a weakly solvating structure: reduced solvent coordination facilitates Li^+^-anion pairing, accelerates anion accumulation at the interface, and triggers controlled decomposition pathways. These processes collectively contribute to early stabilization of ion transport and suppression of parasitic reactions.

- 1. *Supplementary Note. 7*

XPS full spectra and etching depth profiles reveal distinct elemental redistribution on LRMO cathodes cycled in ED and TFD electrolytes (**Figure S26**, **Supporting Information**). In TFD, F and B signals remain nearly constant with depth, indicating subsurface enrichment and the formation of a stable, inorganic‑rich interphase. In contrast, ED shows a continuous increase in F intensity and persistent P signals, reflecting extensive LiPF_6_ decomposition and accumulation of salt‑derived residues. High‑resolution XPS fitting further corroborates these results. In TFD (**Figure S27**, **Supporting Information**), surface C-H and CO_3_^2−^ species progressively decline with etching, while LiF and Li-B-O environments remain stable. The Li_x_PF_y_ signal is markedly reduced, consistent with suppressed continuous LiPF_6_ decomposition under high voltage due to protective LiDFOB-derived CEI, rather than a complete absence of LiPF_6_ decomposition. The early emergence of Me-O bonding in the O 1s spectrum (~6 nm depth) indicates a thin CEI, consistent with TEM measurements showing a compact ~3.7 nm layer (**Figure S25a**, **Supporting Information**). In contrast, ED exhibits increasing C-H and CO_3_^2−^ species, buried fluoride beneath a thick organic overlayer, and persistent Li_x_PF_y_ signals. No Me-O bonding is detected even after 12 nm etching, consistent with TEM evidence of a ~17.2 nm CEI (**Figure S25b**, **Supporting Information**). In summary, TFD promotes the formation of a thin, uniform, and robust inorganic hybrid CEI (LiF/LiBO_2_), effectively suppressing LiPF_6_ decomposition and harmful byproducts. ED, by contrast, induces a thick, organic‑rich, chemically unstable CEI that fails to protect the interface, leading to continuous capacity decay of LRMO cathodes.

- 1. *Supplementary Note. 8*

According to **Figure S34**, (**Supporting Information**), the LRMO cathode cycled in ED exhibits pronounced cation disorder, as reflected by a decrease in the I_(003)_/I_(104)_ ratio from 3.40 (Pristine) to 2.20 after cycling. In contrast, a higher ratio of 3.34 is maintained in TFD, indicating that the CEI formed in TFD effectively mitigates macroscopic structural disorder during cycling. NPD refinement (**Figure 5c**; **Figure S35**, **Supporting Information**) further reveals lattice expansion in both systems. The lattice parameter a increases from 2.859 Å (Pristine) to 2.903 Å (ED) and 2.944 Å (TFD), while c expands from 14.252 Å to 14.367 Å (ED) and 14.453 Å (TFD). These results confirm structural collapse and volume swelling during prolonged cycling, but the moderated expansion in TFD highlight its stabilizing effect. XRD refinement (**Figure 5c**; **Figure S36**, **Supporting Information**) shows consistent trends: increases from 2.857 Å (Pristine) to 2.883 Å (ED) and 2.890 Å (TFD), while c expands from 14.251 Å to 14.325 Å (ED) and 14.338 Å (TFD). These findings align with NPD results, reinforcing that TFD suppresses excessive lattice disorder and volume swelling. In summary, both NPD and XRD analyses confirm that the CEI formed in TFD stabilizes the macroscopic crystal structure of LRMO, alleviating cation disorder and mitigating cycling‑induced volume expansion. The mechanistic origins of this stabilization will be elaborated in the subsequent discussion.

- 1. *Supplementary Note. 9*

Ni K-edge XANES and FT-EXAFS analyses reveal structural trends highly consistent with those observed at the Mn K-edge data (**Figure S37**, **Supporting Information**), underscoring the coupled evolution of transition-metal coordination environments in LRMO cathodes. In the TFD electrolyte, the Ni oxidation state exhibits minimal variation across different cycling states, indicating superior Ni redox reversibility. Correspondingly, FT-EXAFS spectra in the discharged state after 30 cycles show that the positions and intensities of Ni-O and Ni-TM peaks remain nearly identical to those in the pristine state, suggesting well-preserved local coordination and negligible lattice distortion. In contrast, LRMO cathodes cycled in ED electrolyte display pronounced spectral divergence in XANES, with poor overlap between charged and discharged states, indicative of irreversible changes in Ni valence. FT-EXAFS further reveals significant shifts and intensity variations in Ni-O and Ni-TM shells, reflecting bond-length fluctuations and increased structural disorder. These irreversible changes imply that the CEI formed in ED electrolyte fails to effectively suppress transition-metal migration and local lattice distortion. The consistency between Ni and Mn K-edge results suggests that in TFD electrolyte, both Mn-O and Mn-TM features remain stable (**Figure 5d,e**), whereas ED cycling induces parallel distortions in Mn and Ni coordination shells. The lattice instability observed in ED cycling originates from interfacial degradation, while the LiF/LiBO_2_-rich CEI formed in TFD electrolyte effectively stabilizes the interface. Overall, TFD enables reversible Ni coordination and mitigates lattice collapse, thereby preserving the long-term structural integrity of LRMO cathodes and alleviating capacity decay.

**II. Supporting Figures**


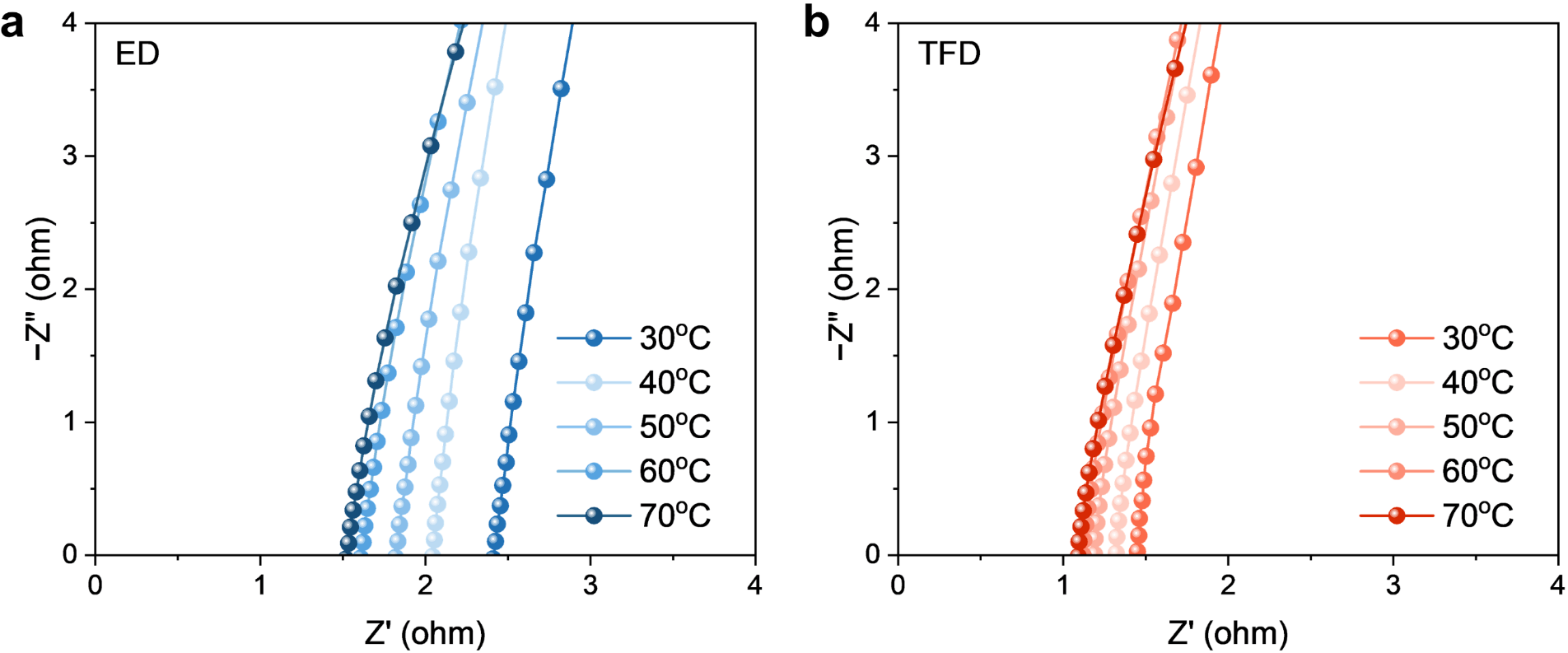


**Figure S1.** Nyquist plots of stainless steel (SS) symmetric cells collected under various temperatures in **a** SS||ED||SS cell, and **b** SS||TFD||SS cell.


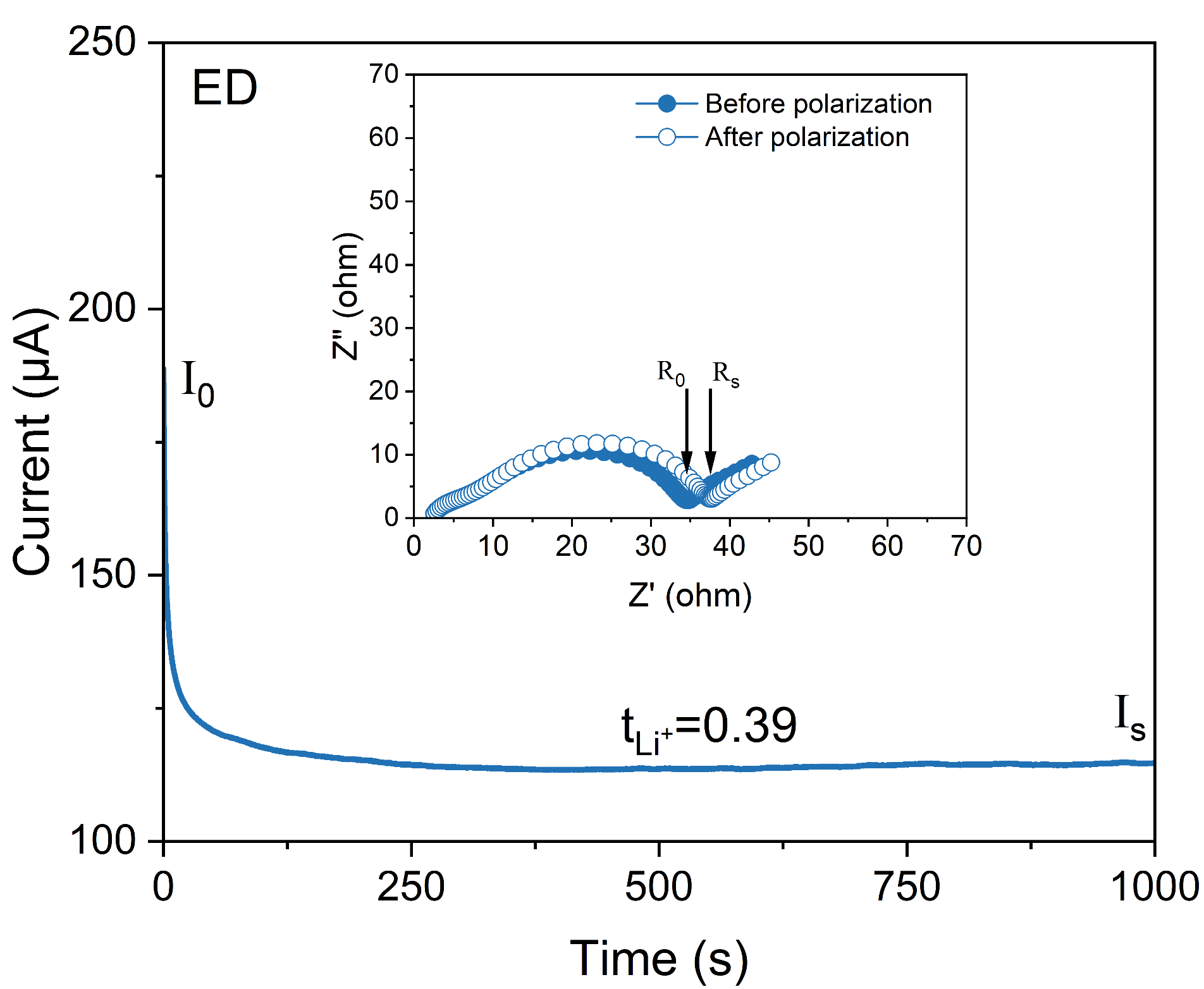


**Figure S2.** Current-time curves following DC polarization for Li/ED/Li cell, the inset corresponds to AC impedance spectra before and after DC polarization.

**Figure S3.** **a** Electrostatic potential distribution for TFTFE. **b** Binding energies between Li^+^ and TFTFE.


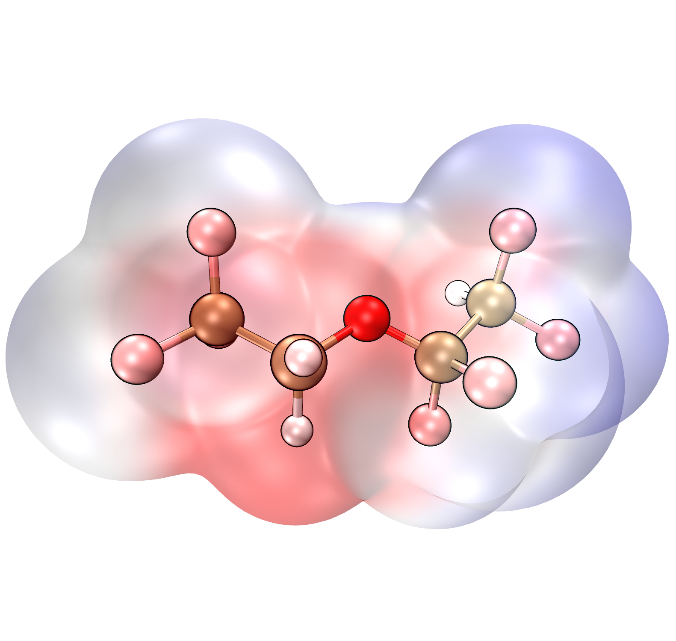

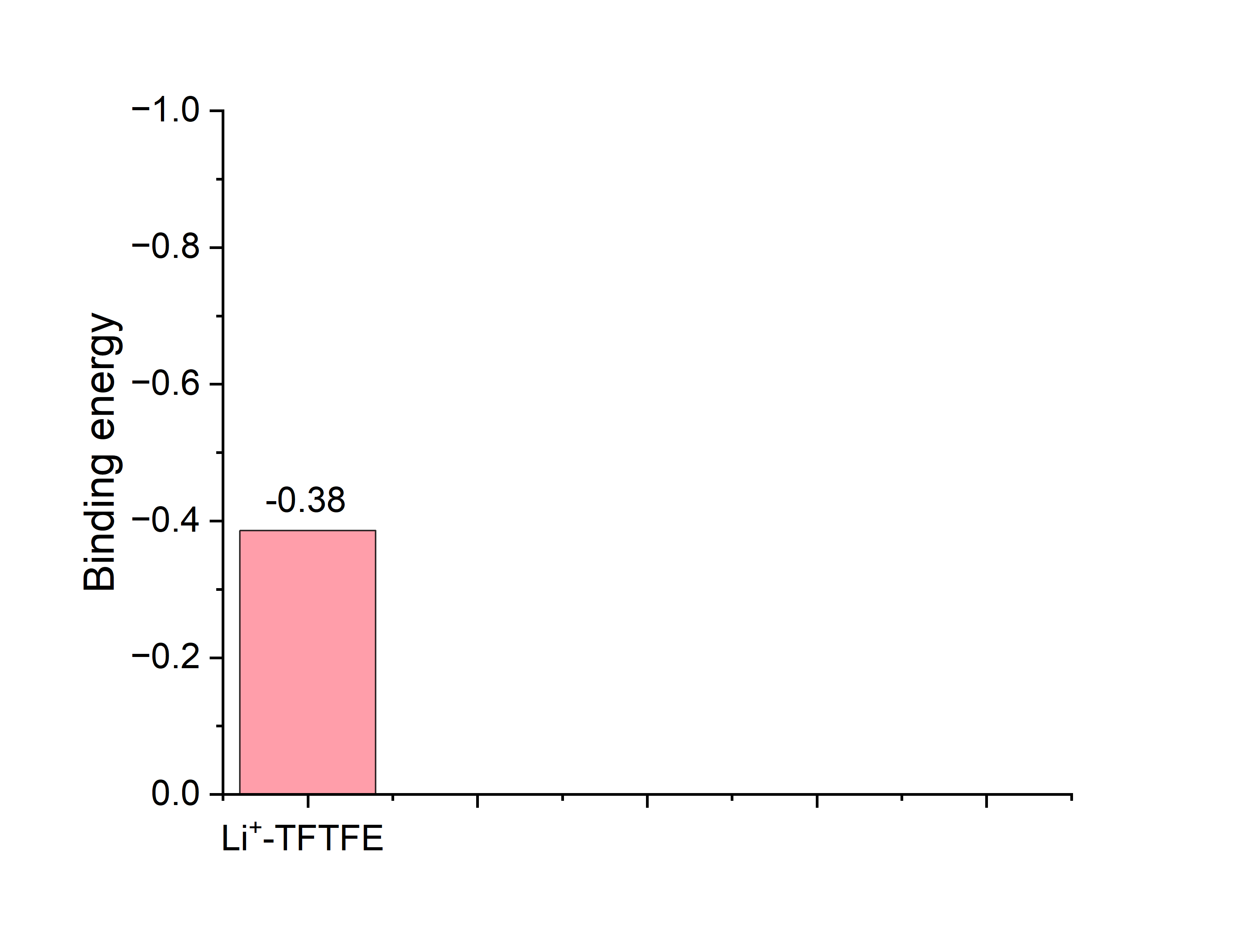


**b**

**a**


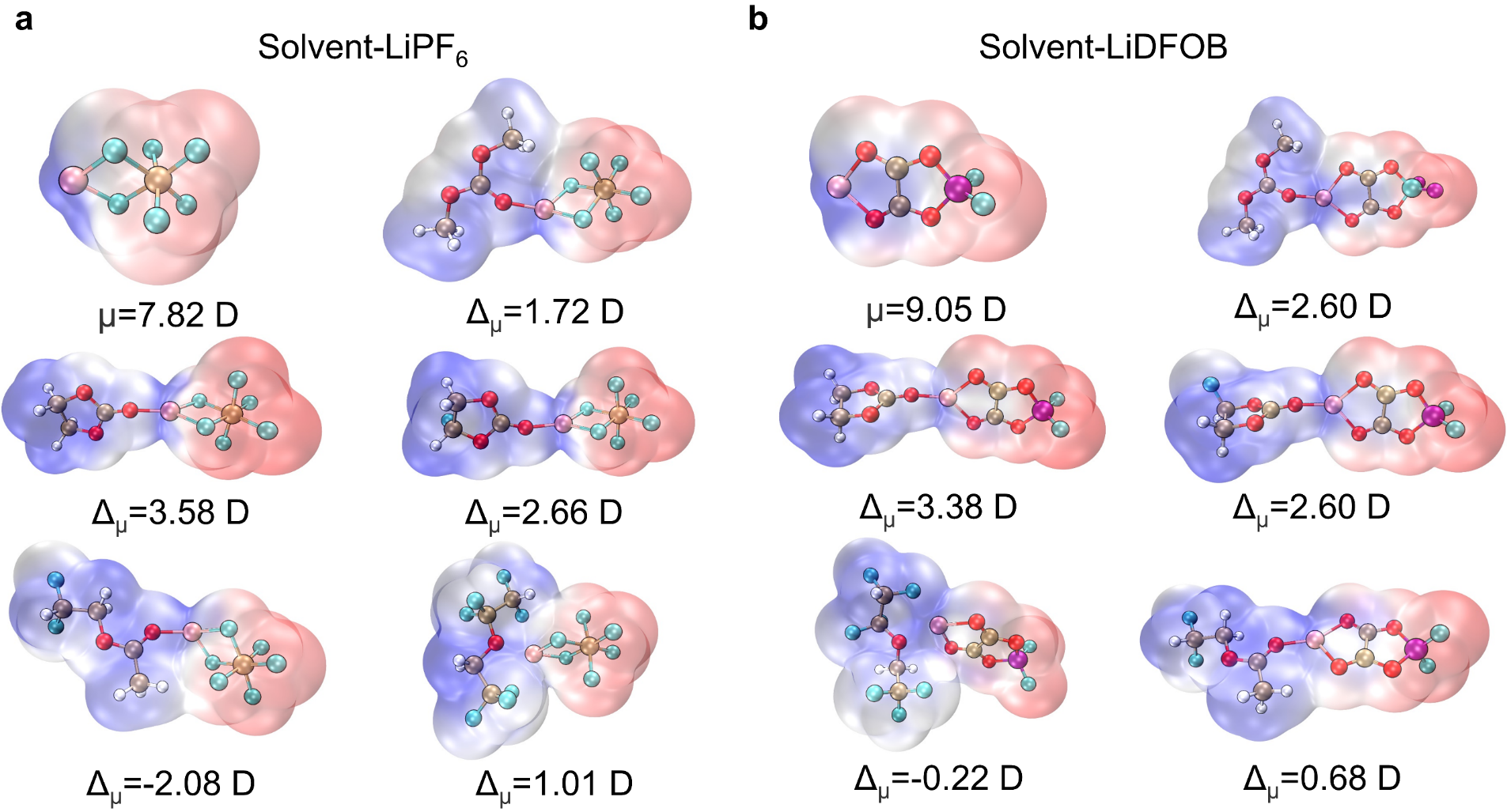


**Figure S4.** Optimized structure of Li^+^ with electrolyte components and **a** PF_6_^−^, **b** DFOB^−^.

**
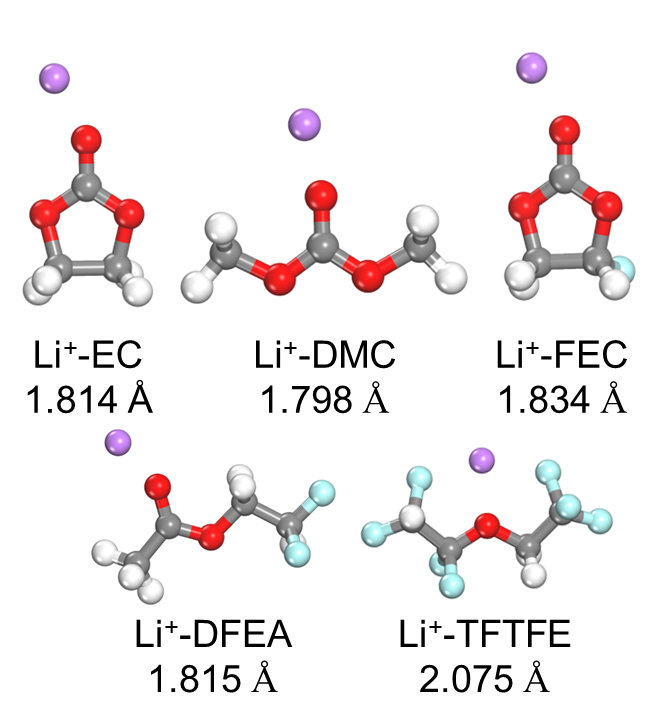
**

**Figure S5.** Optimized structure of Li^+^ with electrolyte components.


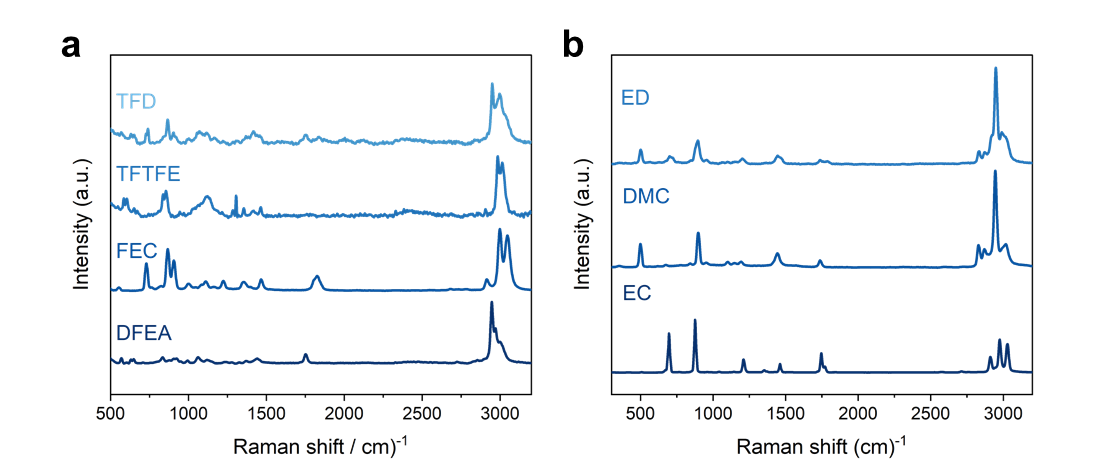


**Figure S6.** Raman spectra of the corresponding solvents and electrolytes in **a** TFD, **b** ED.


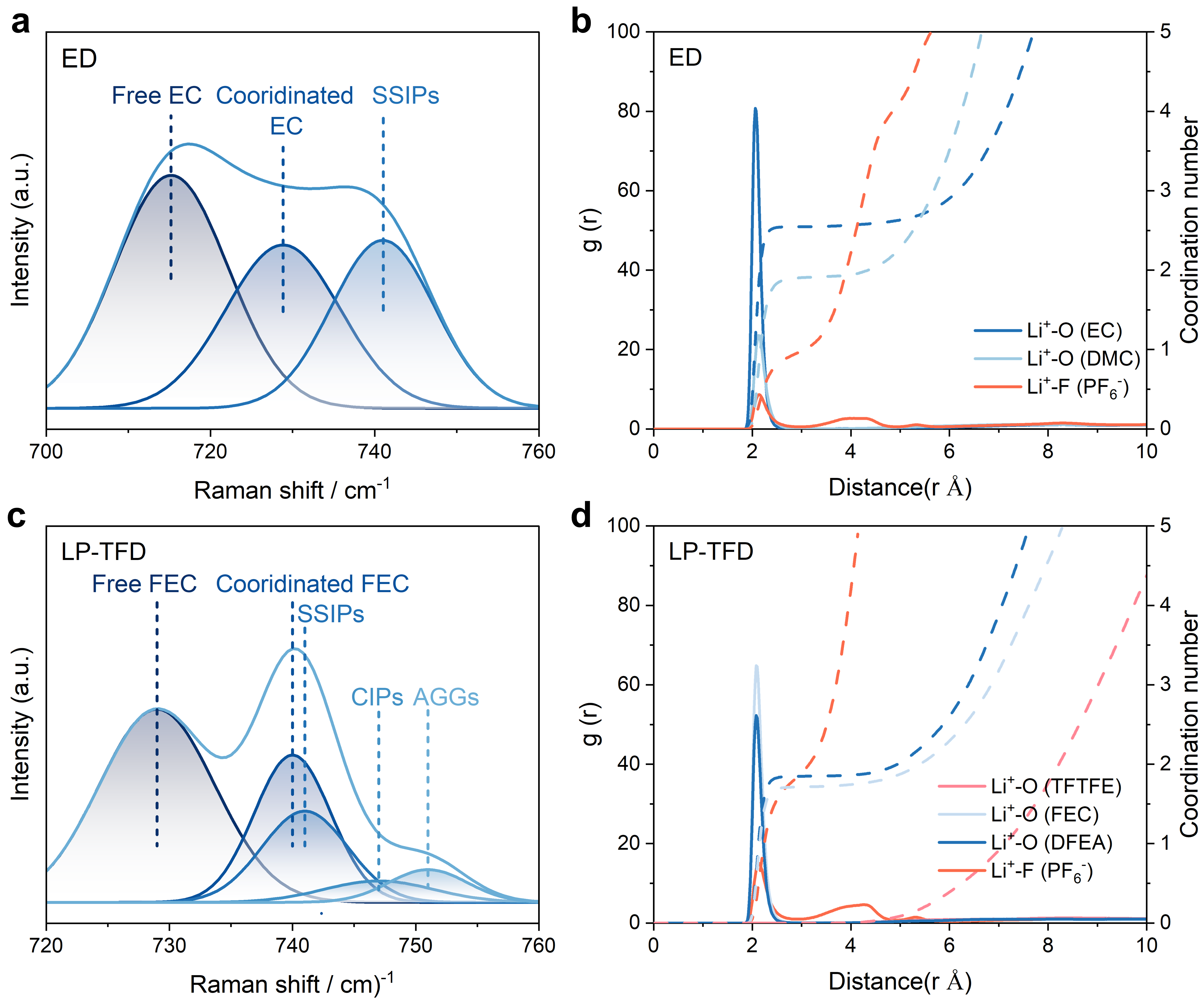


**Figure S7.** **a)** Raman spectra fitting result for ED electrolyte. **b)** Radial distribution functions and coordination numbers of Li^+^ from MD simulations of ED. **c)** Raman spectra fitting result for LP-TFD electrolyte. **d)** Radial distribution functions and coordination numbers of Li^+^ from MD simulations of LP-TFD.


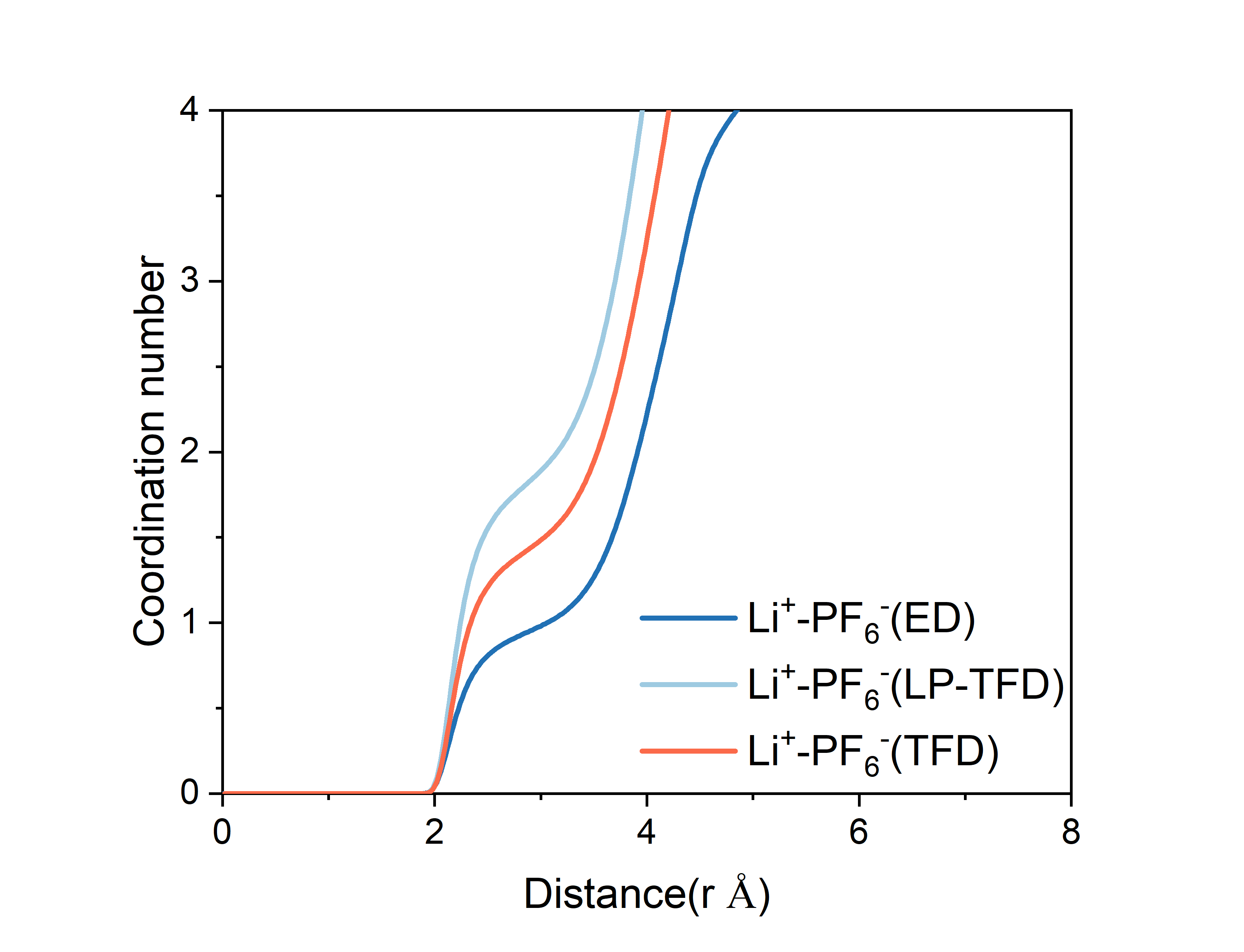


**Figure S8.** Comparison of coordination number of Li^+^-PF_6_^−^ pair in ED, LP-TFD and TFD.


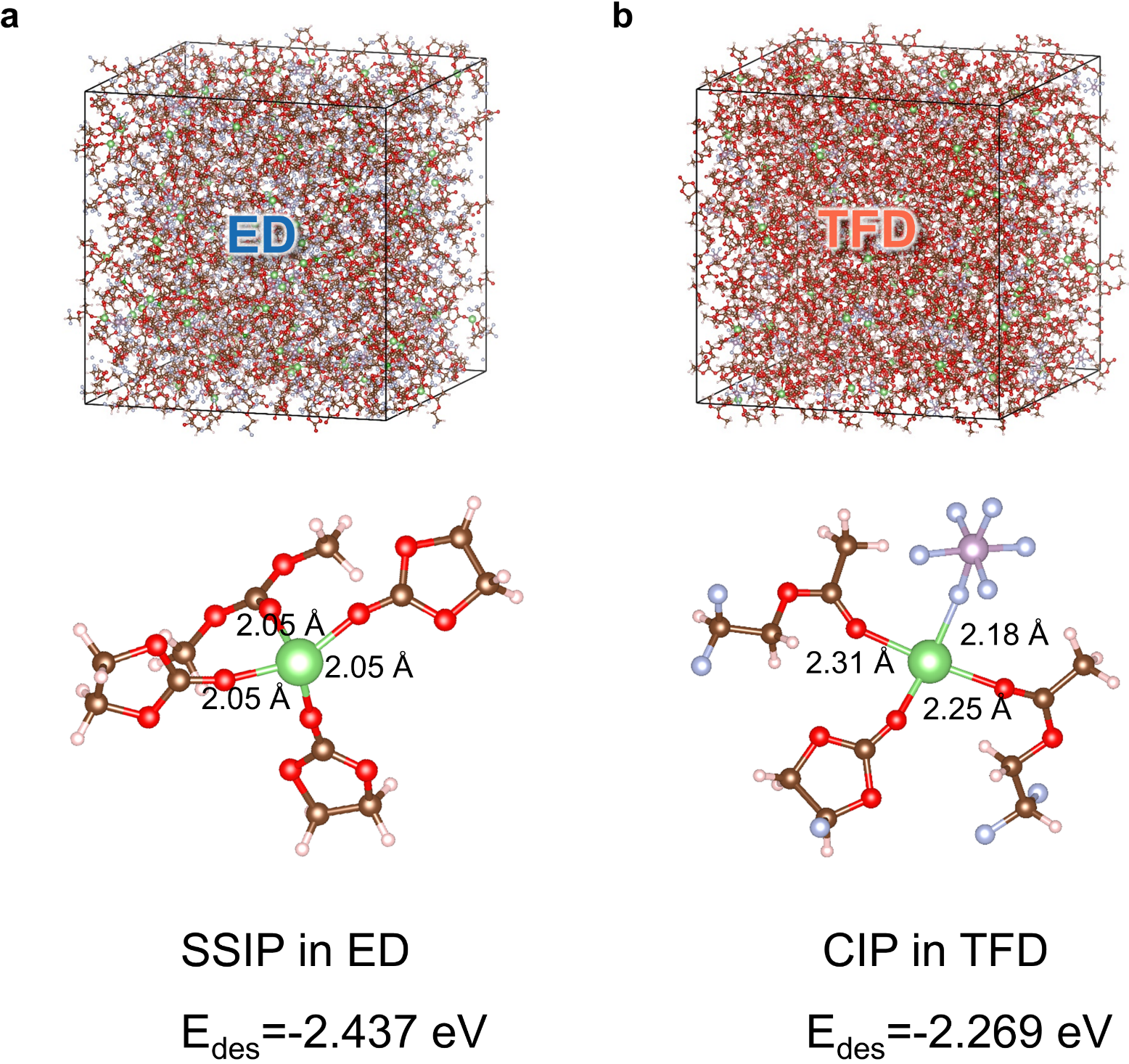


**Figure S9.** Snapshots obtained by MD simulation and enlarged view of solvated structure for **a** ED, **b** TFD.


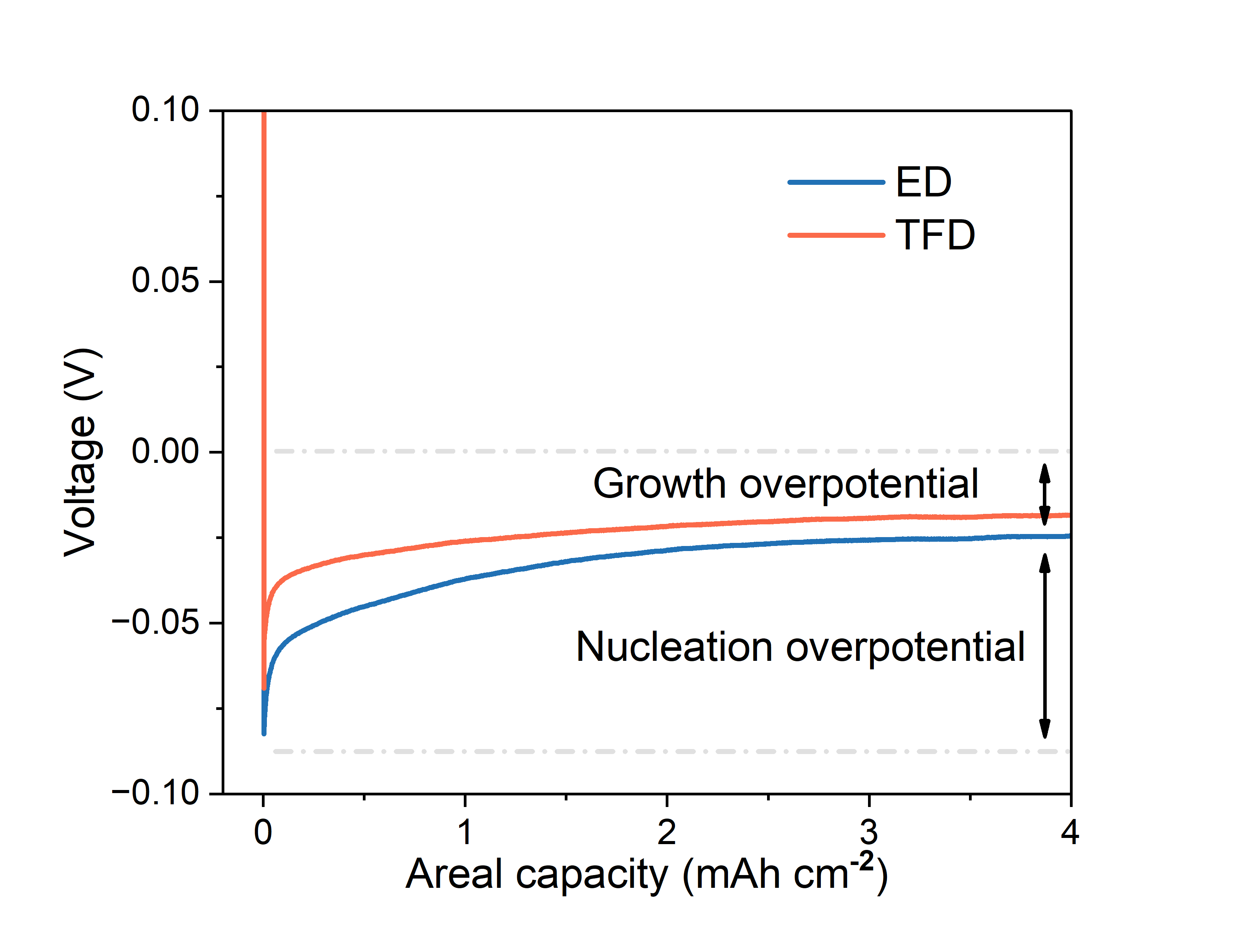


**Figure S10.** Voltage-capacity profiles for the Li plating process on Cu substrate with ED and TFD electrolytes in Li/Cu cell.


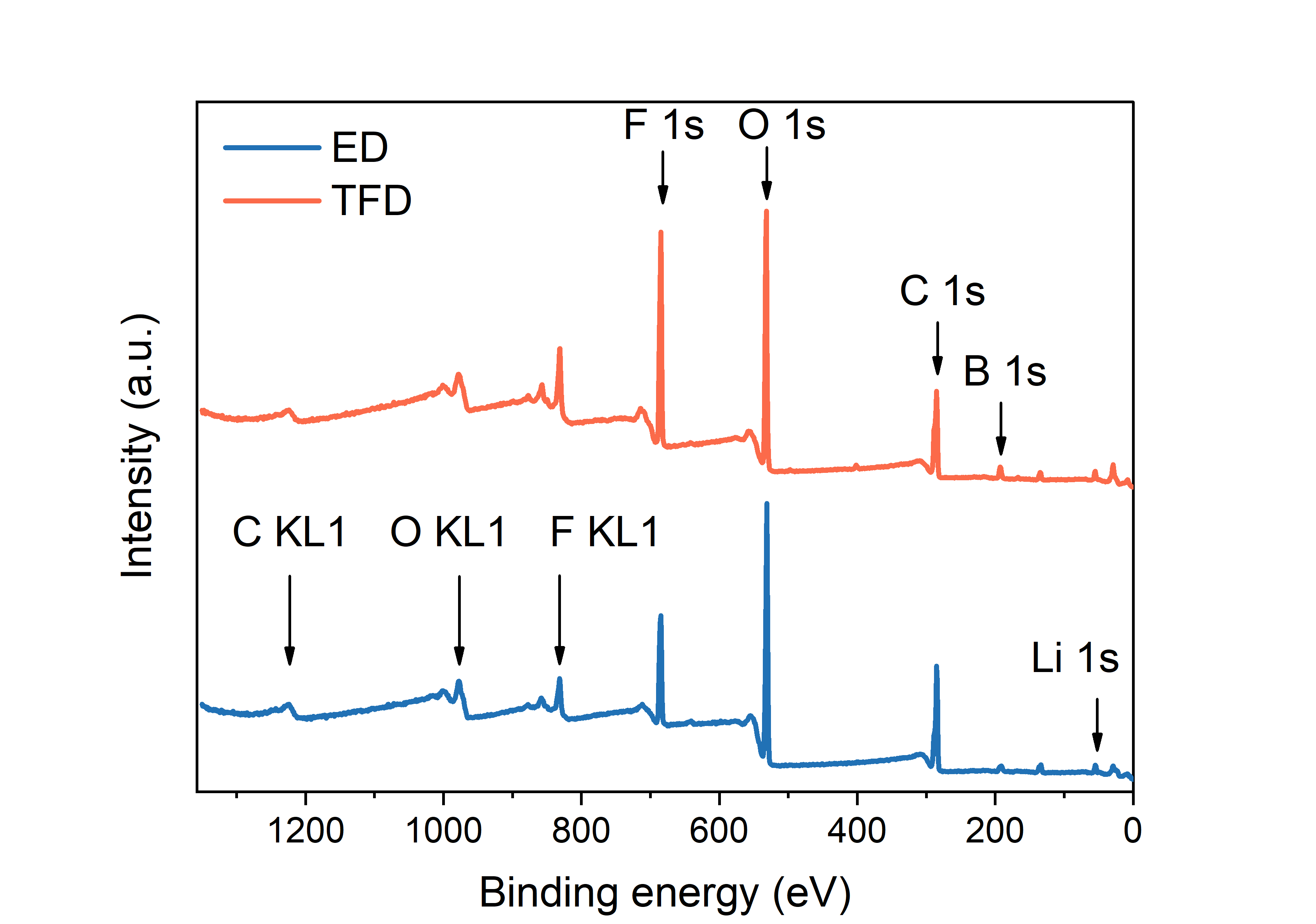


**Figure S11.** The XPS full spectra taken from cycled Li metal surface in ED and TFD.


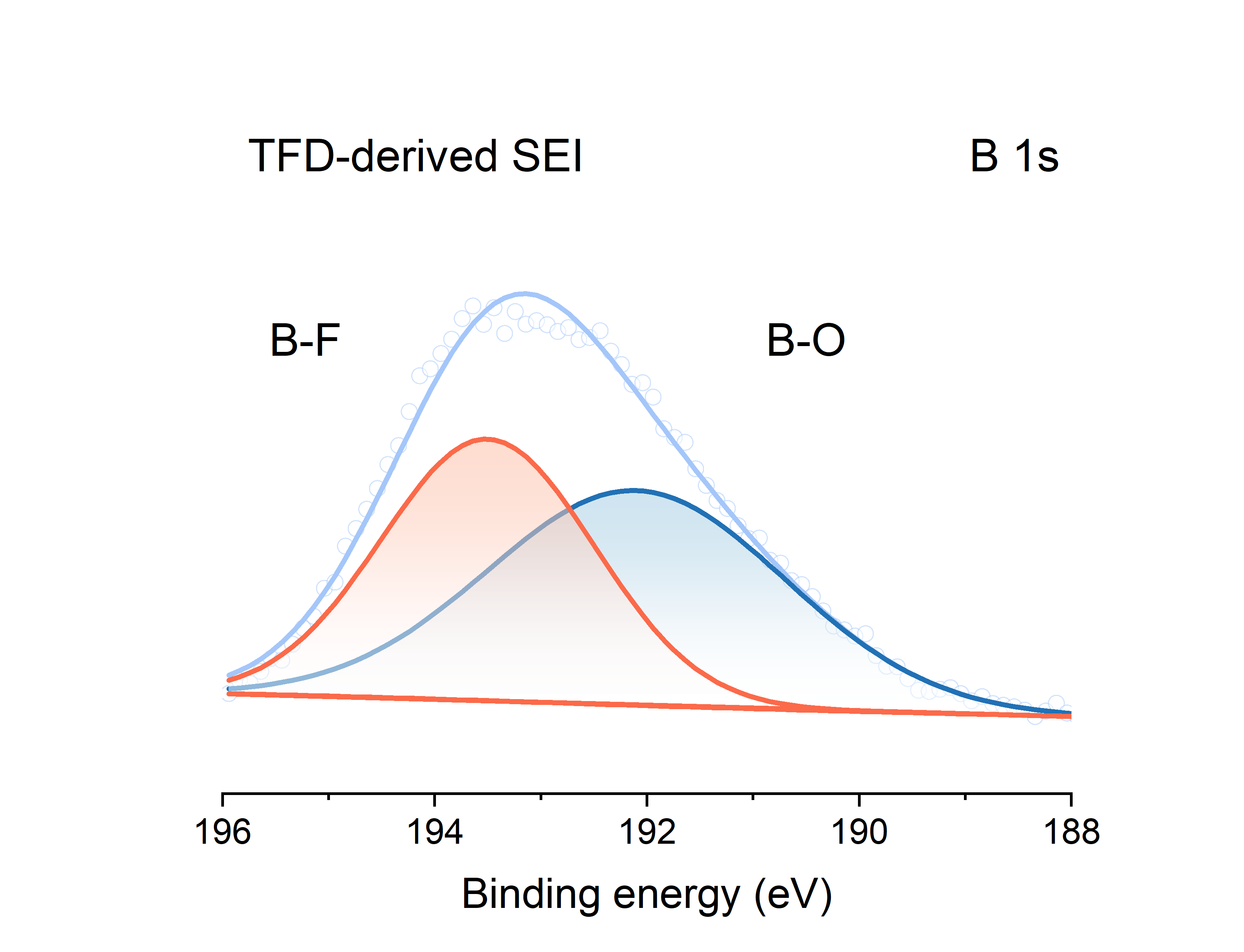


**Figure S12.** Detailed fitting XPS results of B 1s spectra taken from cycled Li metal surface using TFD as electrolyte.


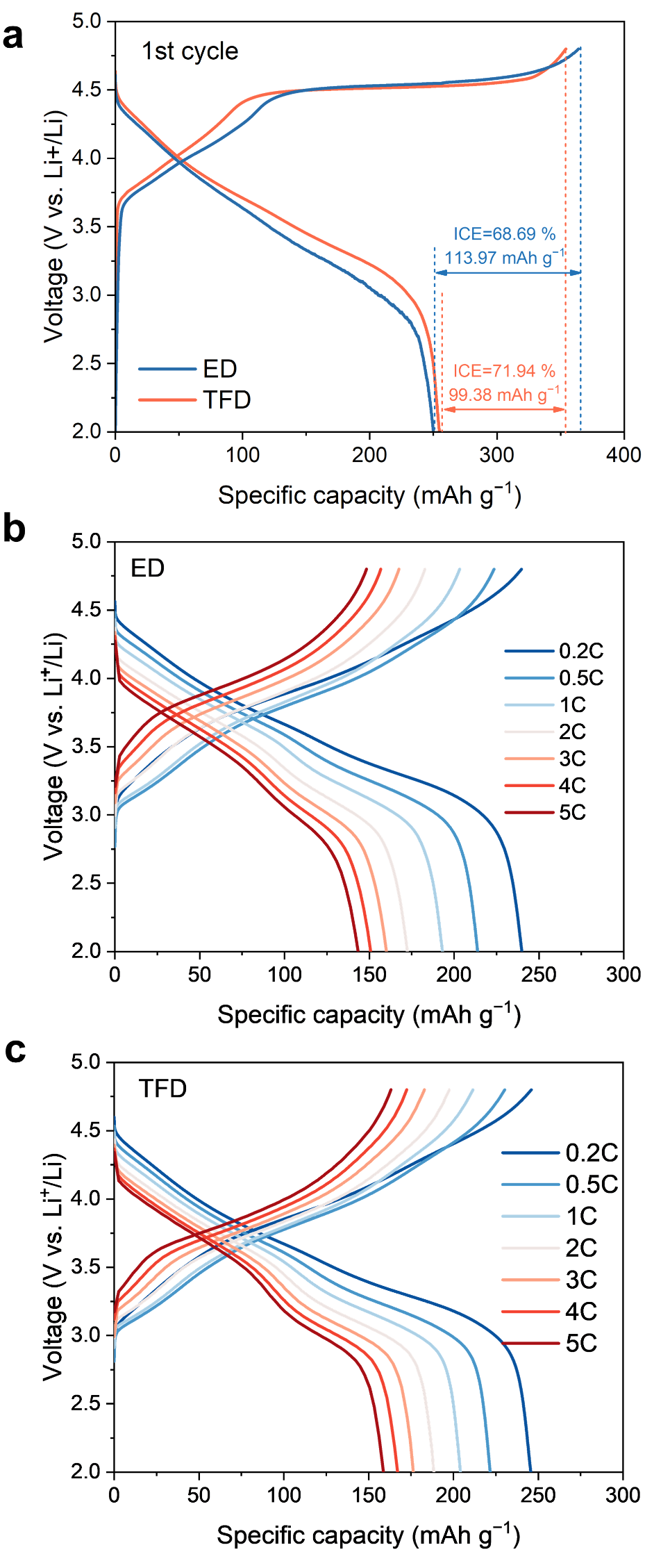


**Figure S13.** **a** Comparison of the initial galvanostatic charge-discharge (GCD) curves in LRMO||ED||Li cell and LRMO||TFD||Li cell. Corresponding GCD curves under different C rates in **b** LRMO||ED||Li cell, **c** LRMO||TFD||Li cell.


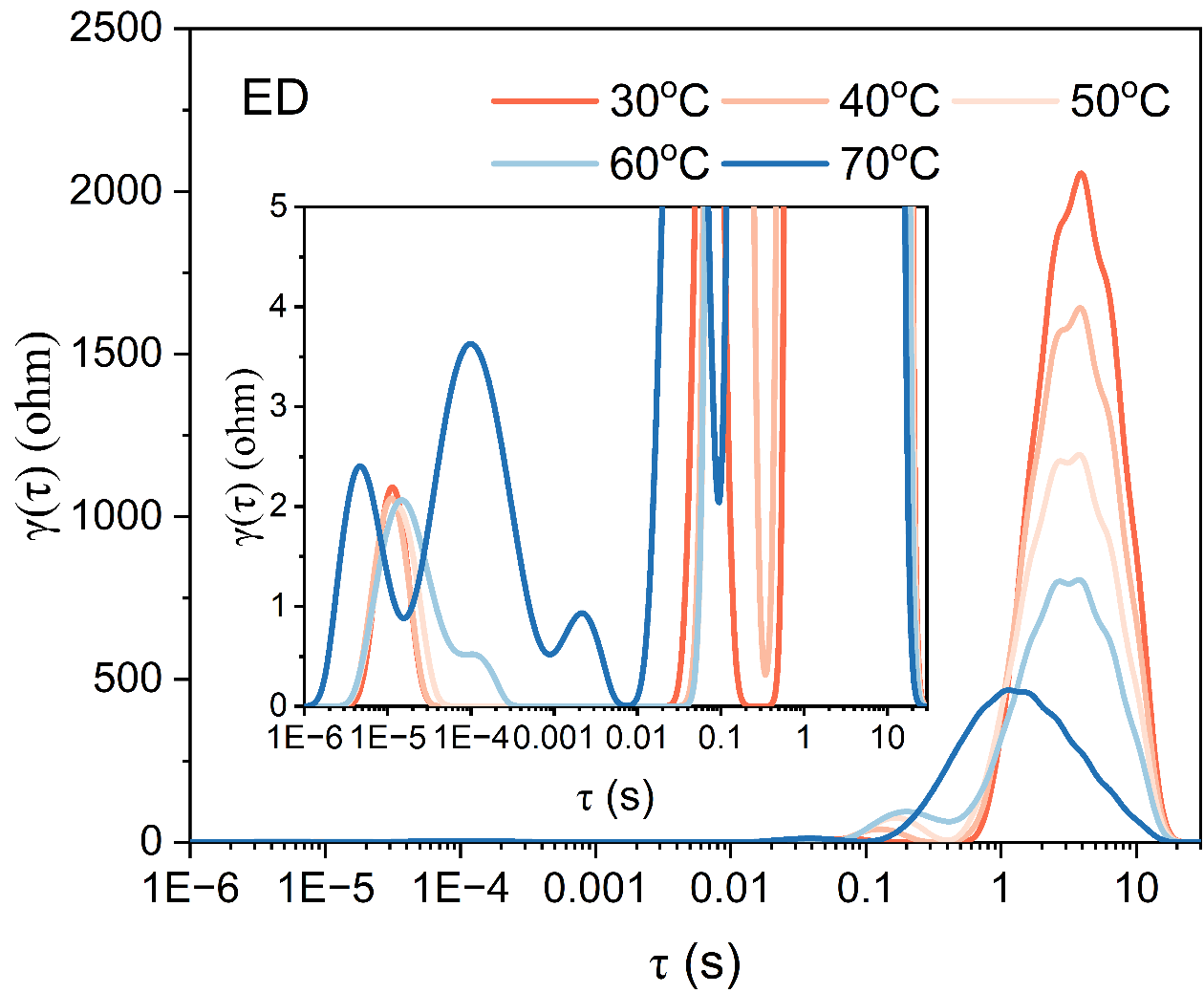


**Figure S14.** DRT results for LRMO||ED||LRMO symmetrical cell at various temperatures.


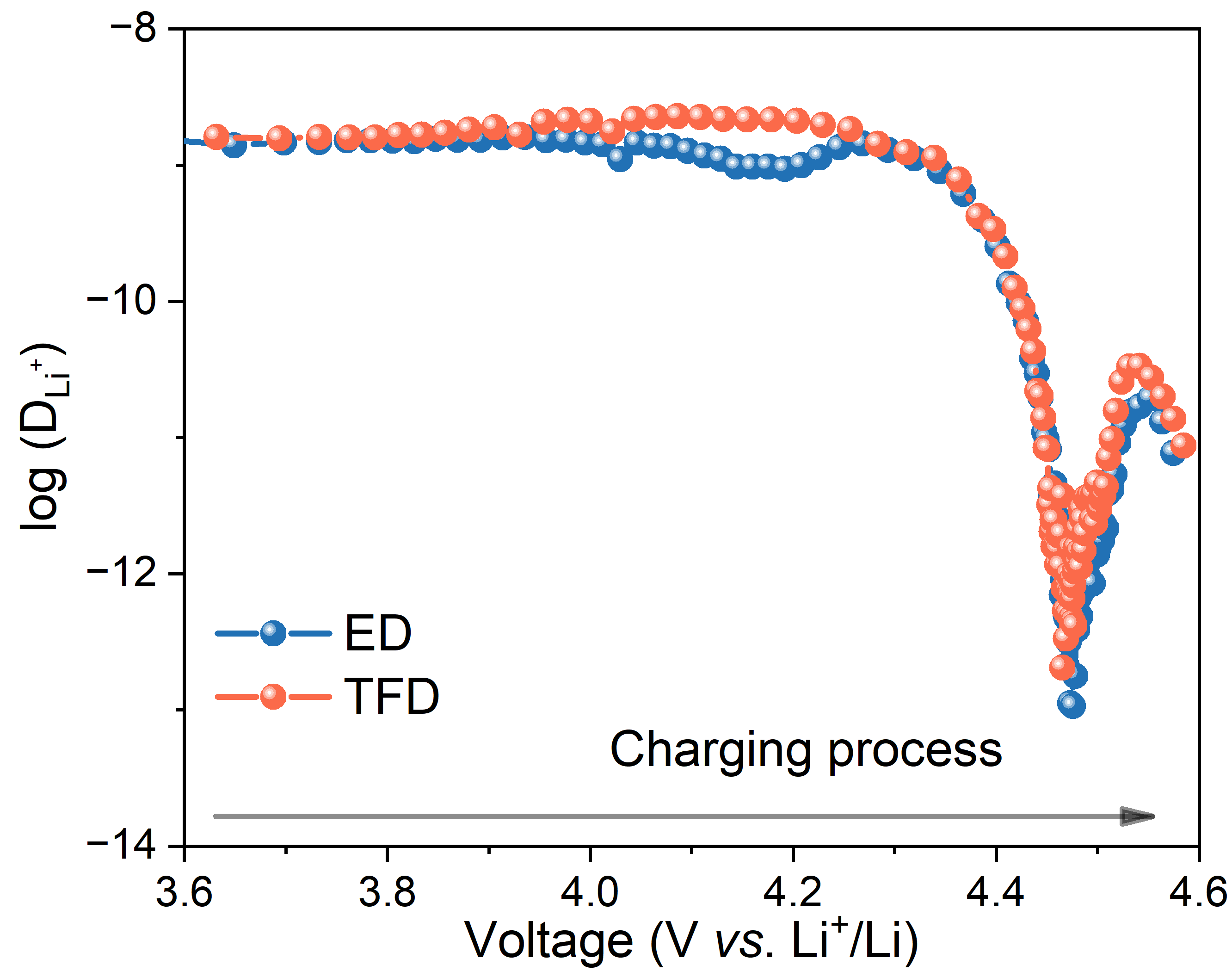


**Figure S15.** GITT results of LRMO||Li cells during charging process in ED and TFD electrolytes.


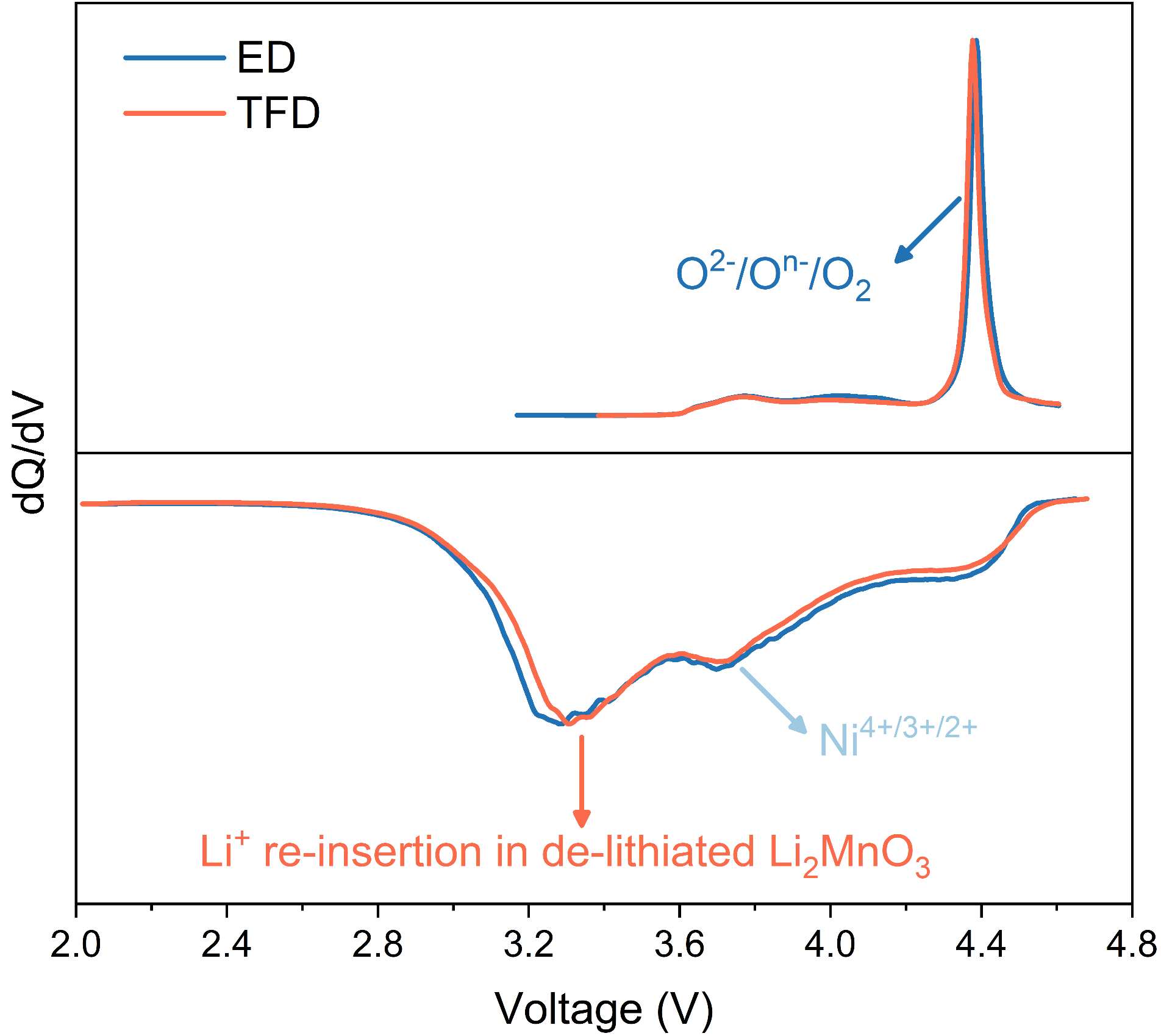


**Figure S16.** dQ/dV profiles of the initial GCD curves extracted from LRMO||Li cells cycled in ED and TFD


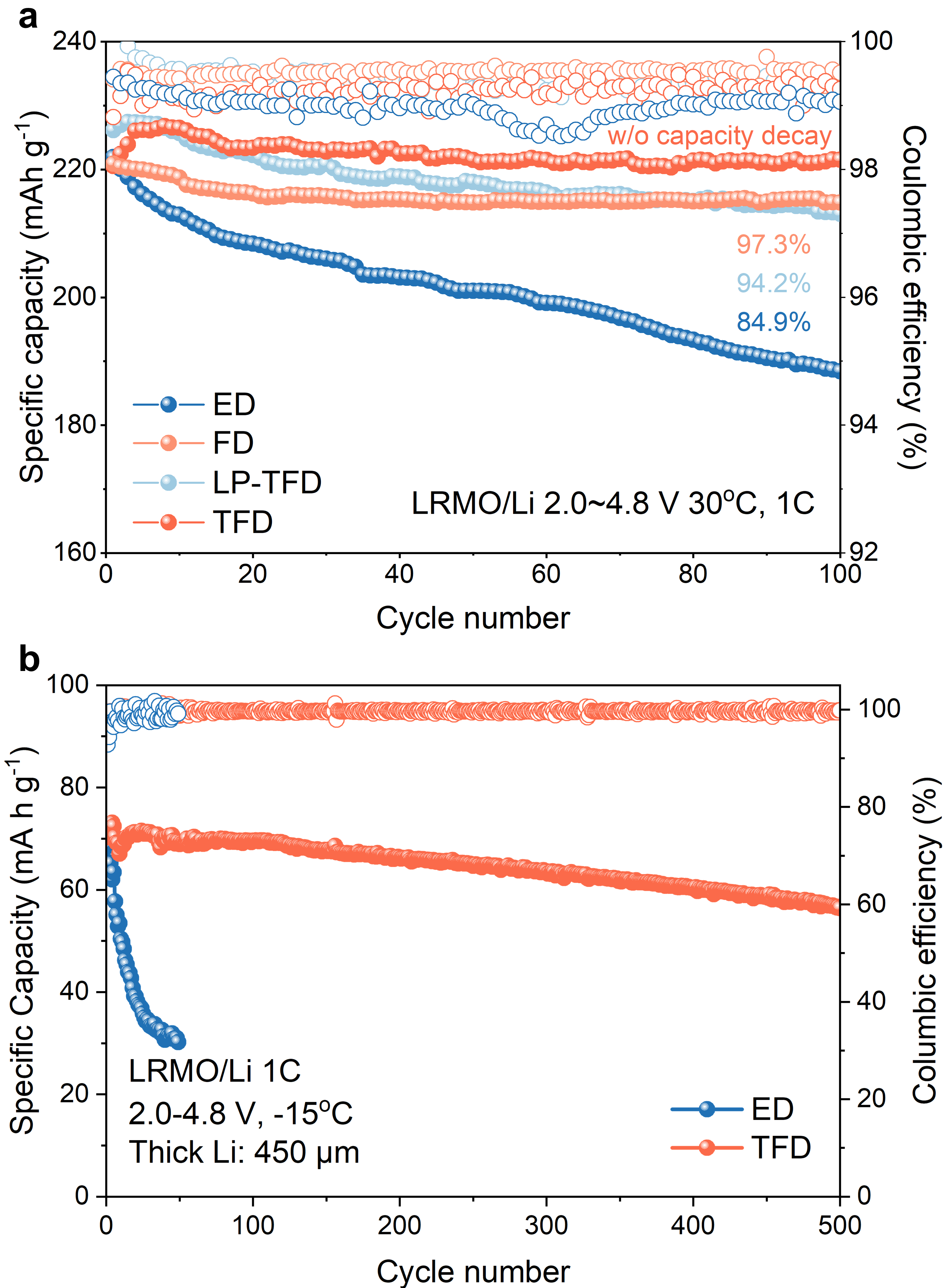


**Figure S17.** a) Cycling performance of LRMO||Li cells using different electrolytes at 1 C (1C=200 mA g^−1^). b) Cycling performance of LRMO||ED||Li and LRMO||TFD||Li cells under 1C at −15^o^C (1C=200 mA g^−1^).


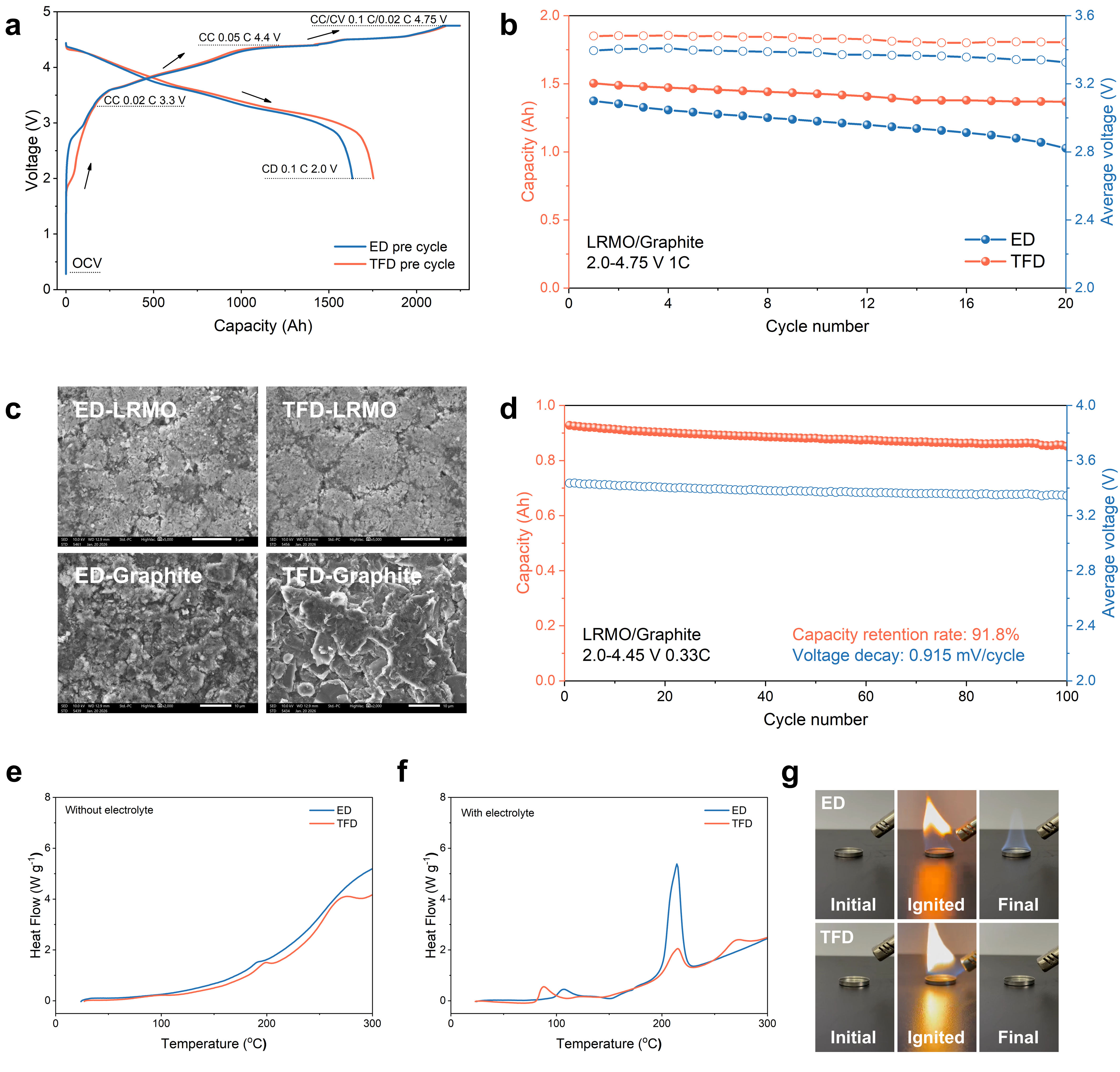


**Figure S18.** a) Comparison of the pre-cycle GCD curves for SEI formation in LRMO||ED||Graphite cell and LRMO||TFD||Graphite cell. b) Cycling performance for the LRMO||ED||Graphite and LRMO||TFD||Graphite pouch cell at 1 C and 2.0 – 4.75 V (equal to 4.80 V *vs*. Li^+^/Li). c) Post-mortem SEM analysis of cycled Graphite and LRMO in LRMO||Graphite pouch cell at 1 C and 2.0 – 4.75 V (equal to 4.80 V *vs*. Li^+^/Li). d) Cycling performance for the LRMO||TFD||Graphite pouch cell at 0.33 C and 2.0 – 4.45 V (equal to 4.50 V *vs*. Li^+^/Li). DSC analysis for the charged LRMO cathode (4.8 V) using ED and TFD electrolytes under the condition of e) without electrolyte, and f) with electrolyte. g) Flame retardancy testing of ED and TFD electrolytes.


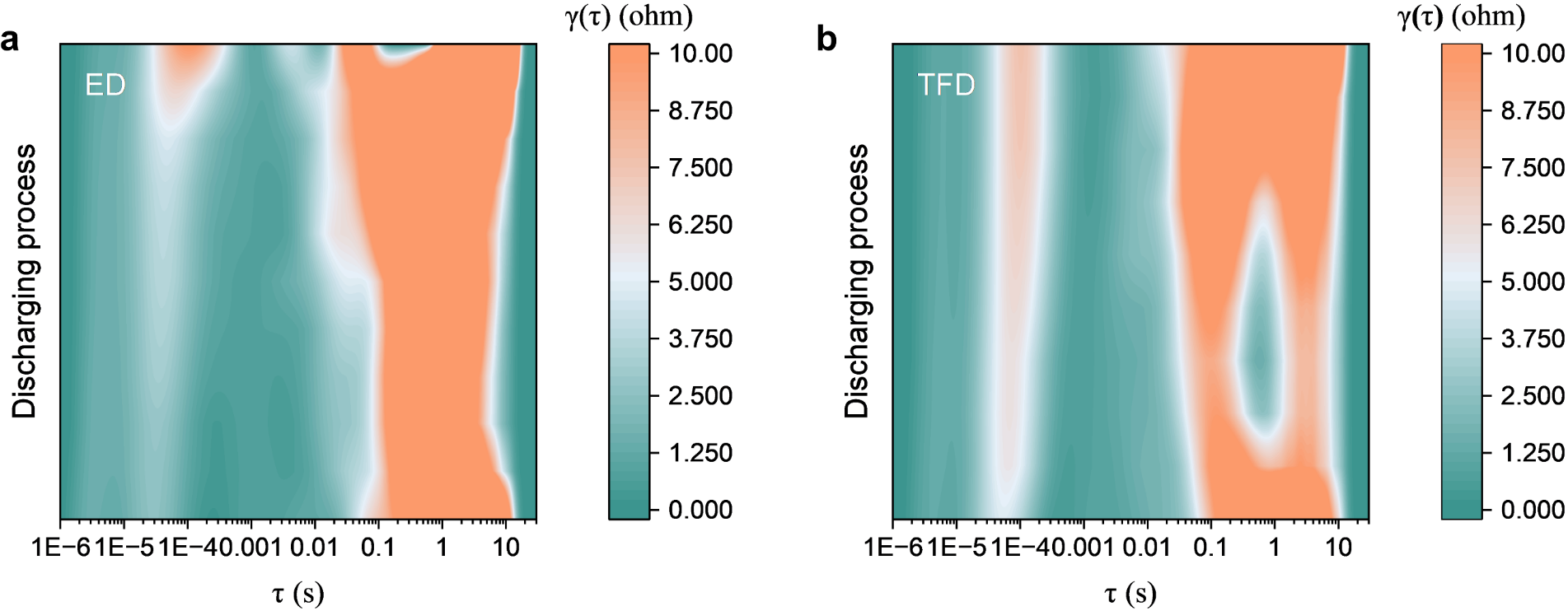
**Figure S19.** Corresponding DRT results for *In-situ* GEIS of the first discharging process.

c
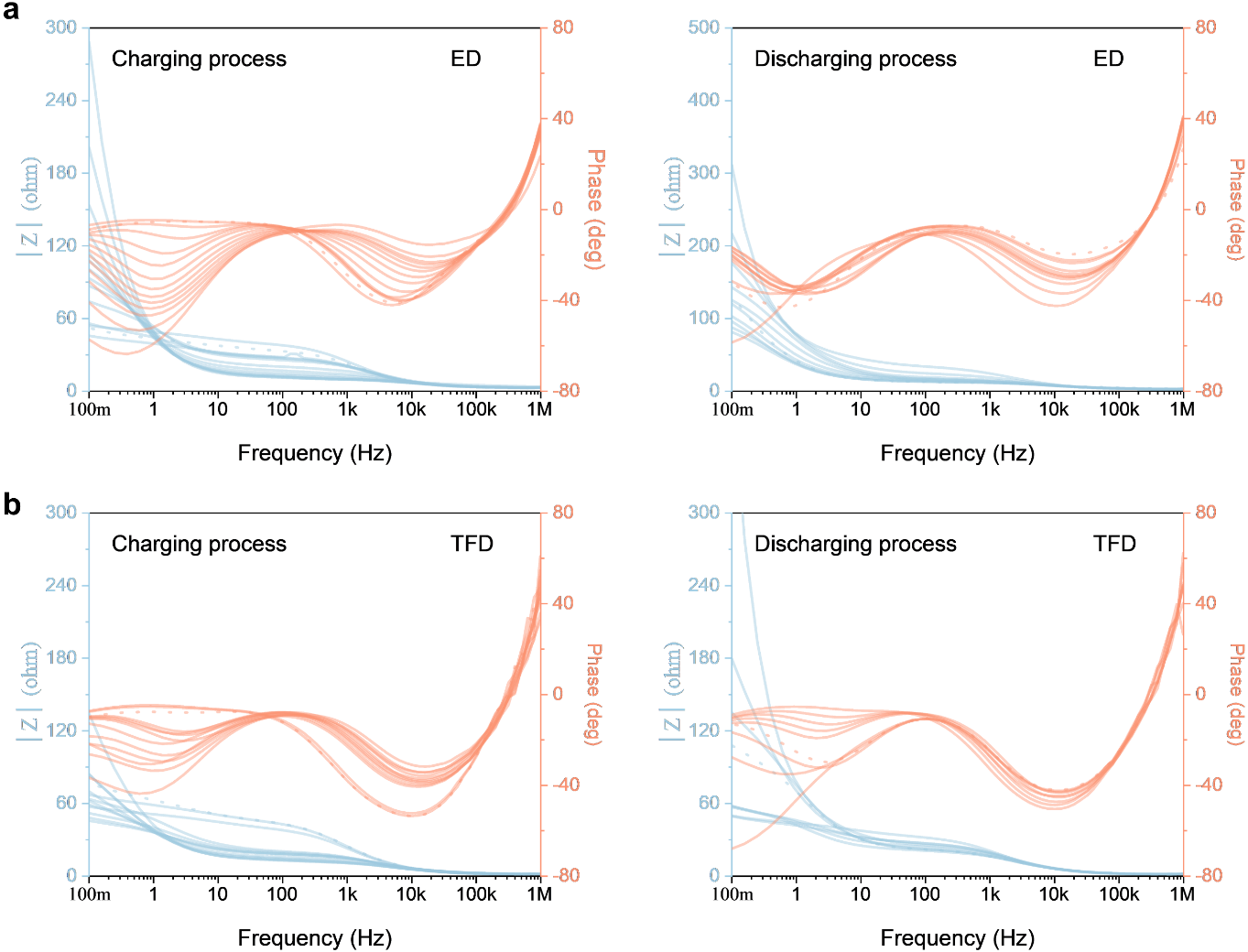


**Figure S20.** Corresponding bode plots of the IS-GEIS results during the first charging and discharging process in **a** LRMO||ED||Li cell, **b** LRMO||TFD||Li cell.


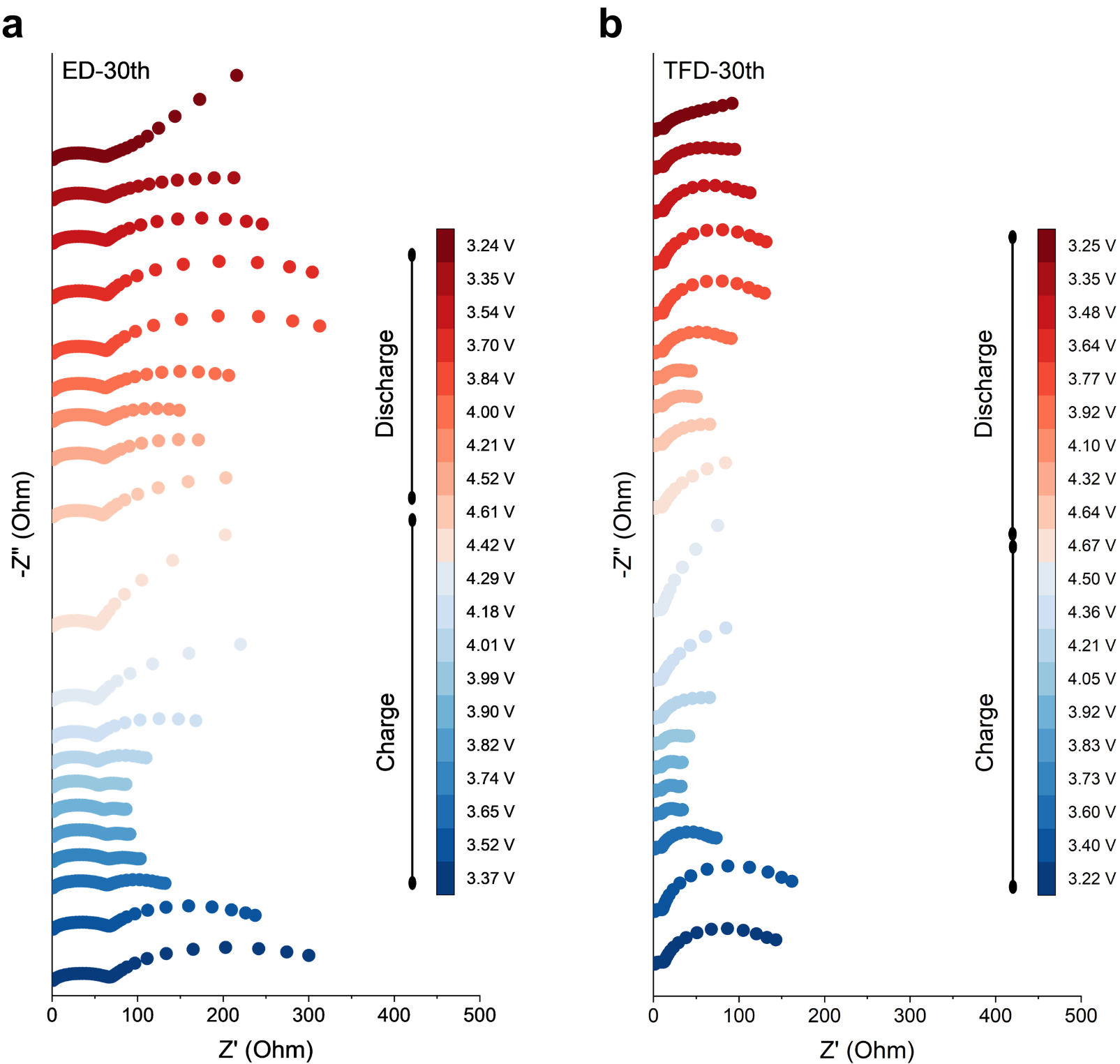


**Figure S21.** *In-situ* GEIS of LRMO||Li cells to reveal the dynamic evolution of CEI during the 30^th^ charge/discharge process cycled in **a** ED, and **b** TFD electrolytes.


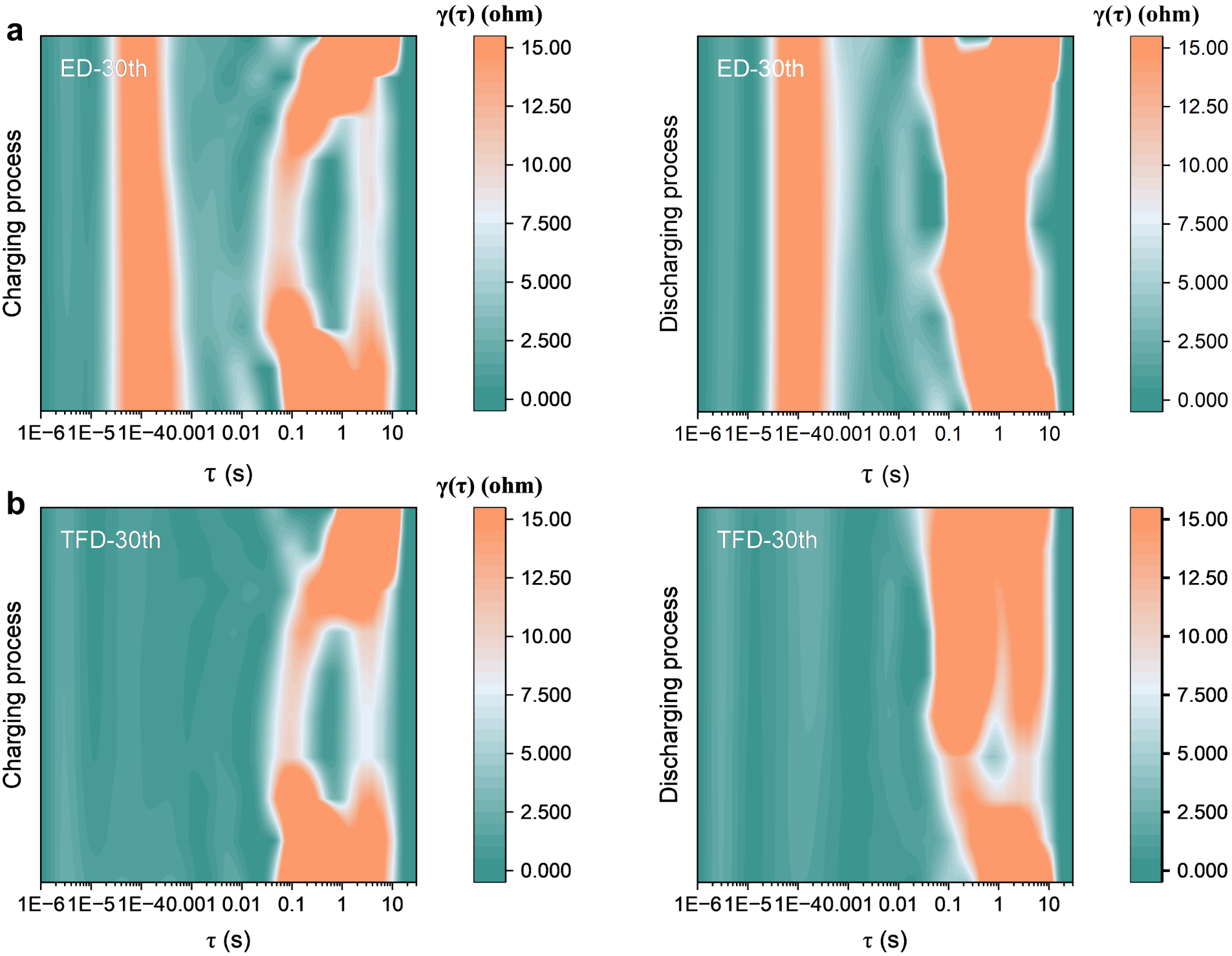
**Figure S22.** Corresponding DRT results for *In-situ* GEIS of LRMO||Li cells in the 30^th^ charging and discharging process employing **a** ED, and **b** TFD electrolytes.


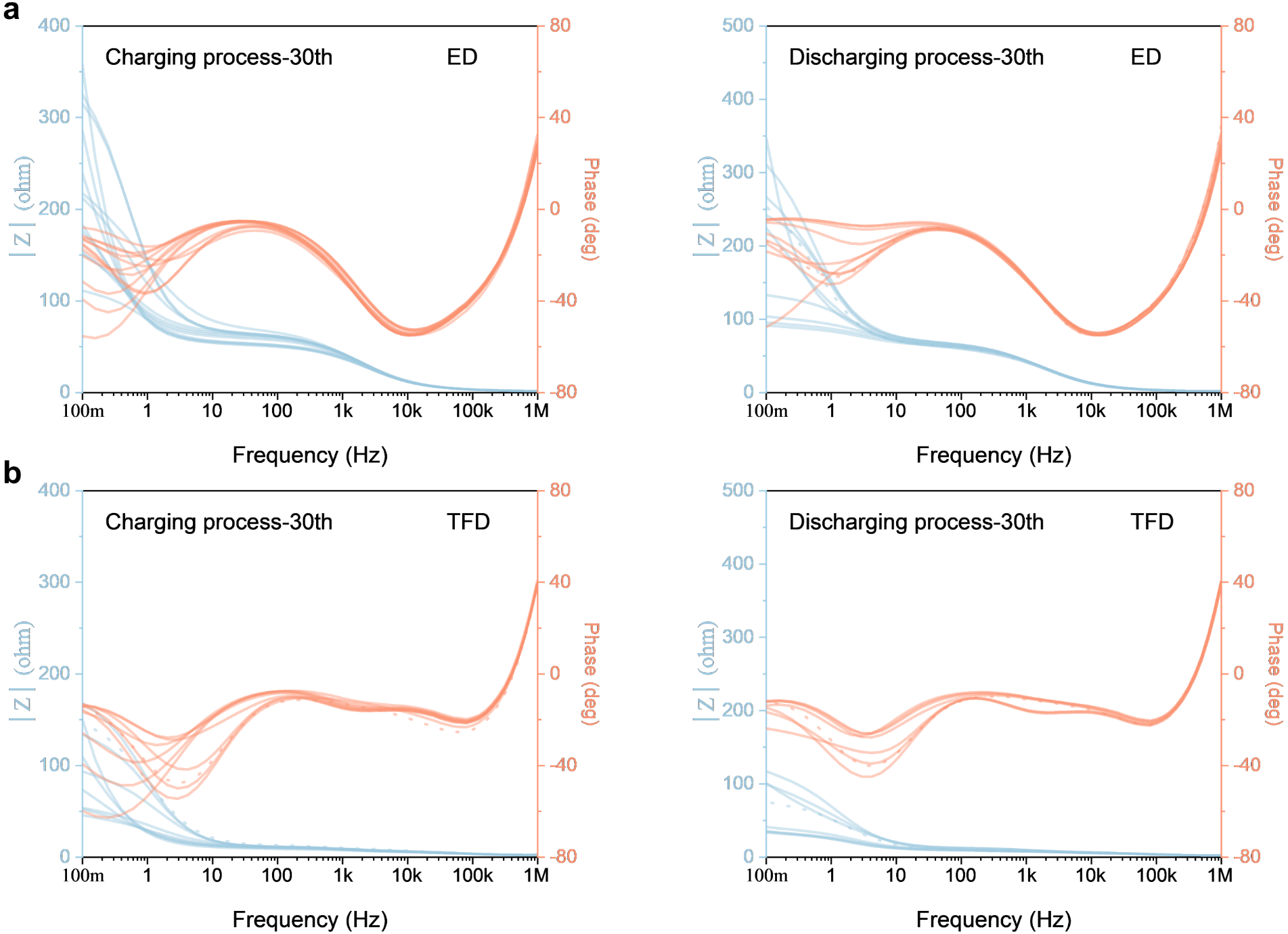
**Figure S23.** Corresponding bode plots of the IS-GEIS results during the 30^th^ charging and discharging process in **a** LRMO||ED||Li cell, **b** LRMO||TFD||Li cell.


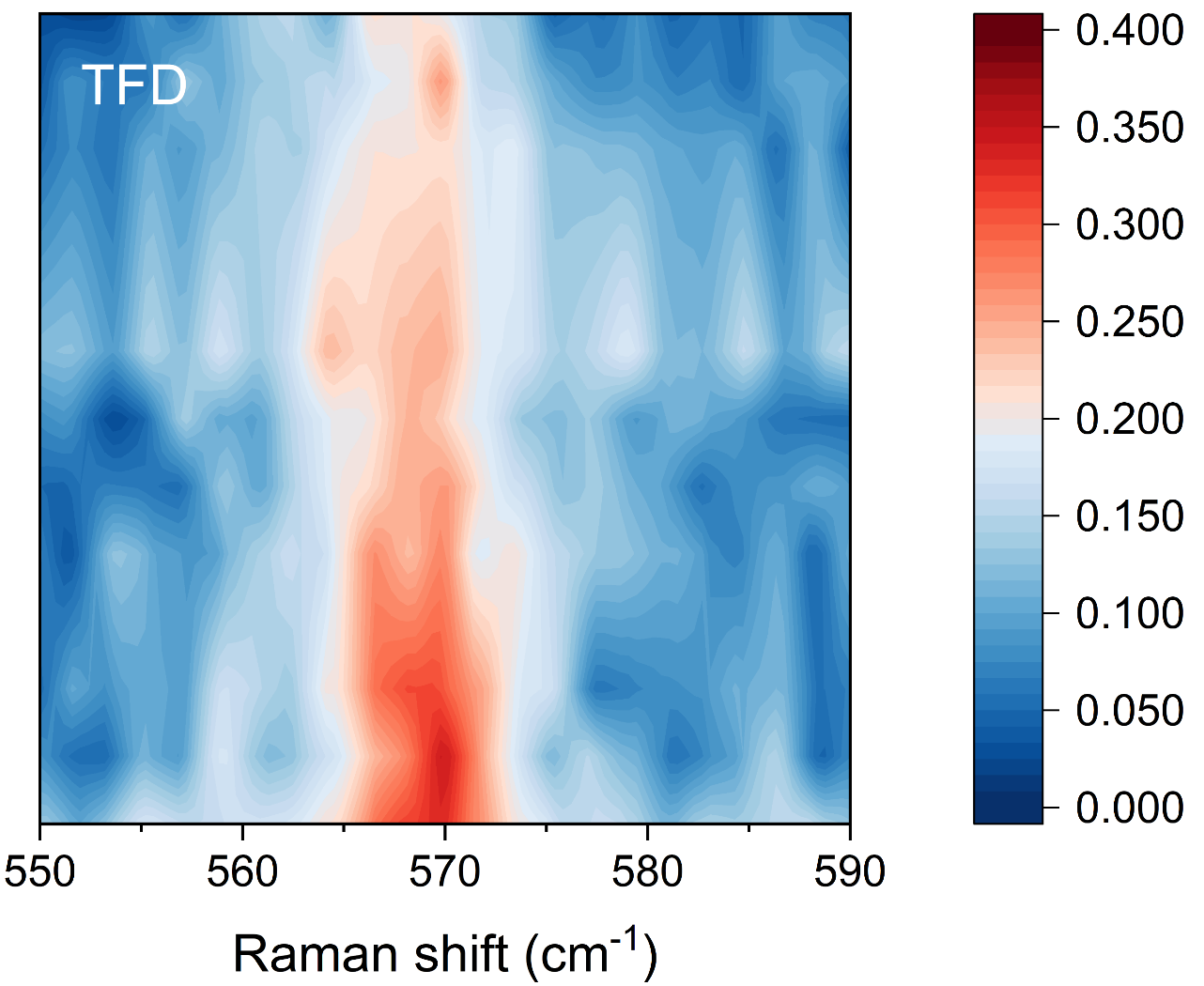


**Figure S24.** *In-situ* Raman spectra of LRMO||TFD||Li cell to reveal the dynamic evolution of solvated structure.


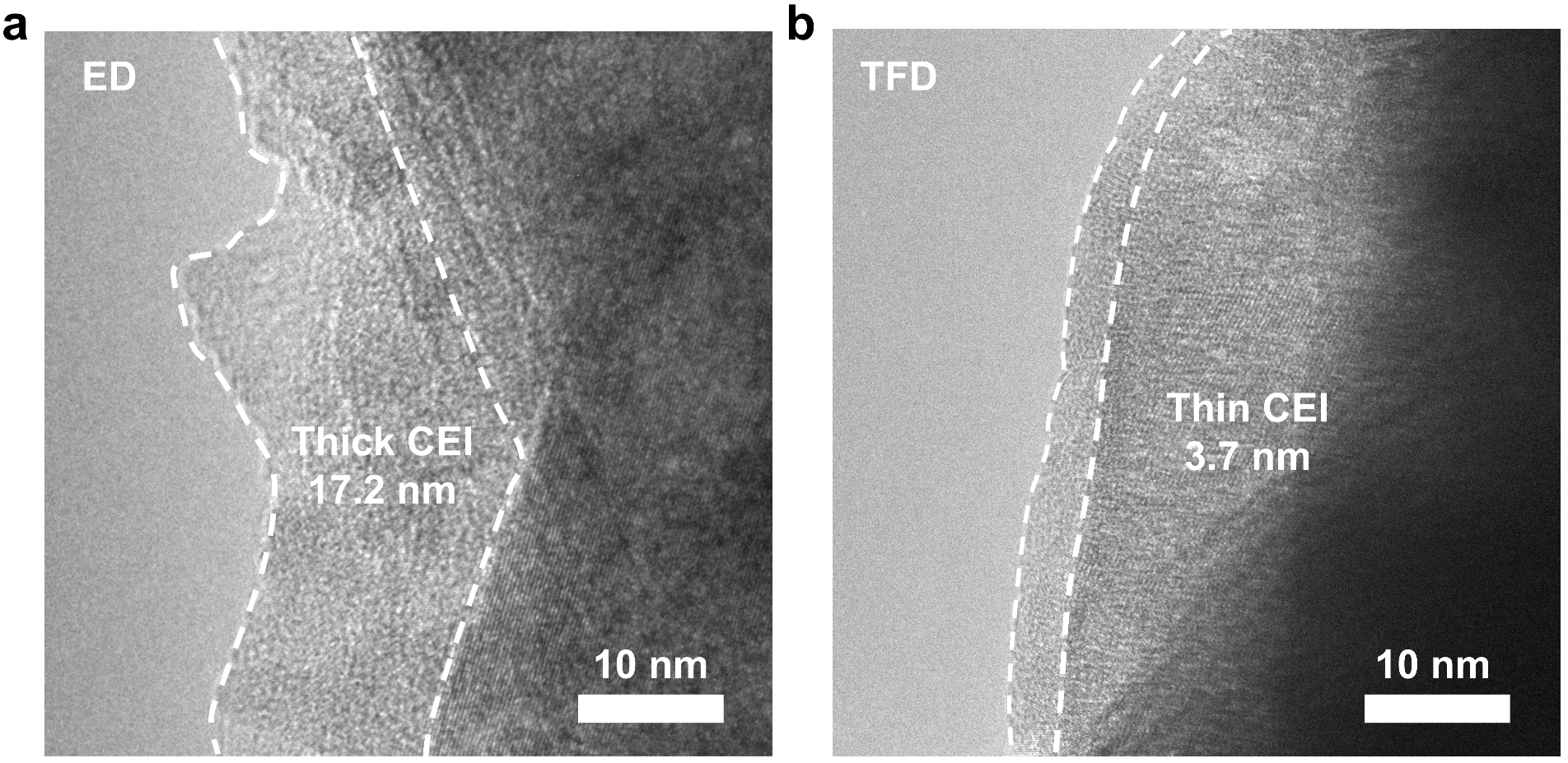


**Figure S25.** TEM images and Corresponding EDS mapping results of LRMO cathodes obtained from a LRMO||ED||Li and LRMO||TFD||Li cells after 30 cycles at 1 C.


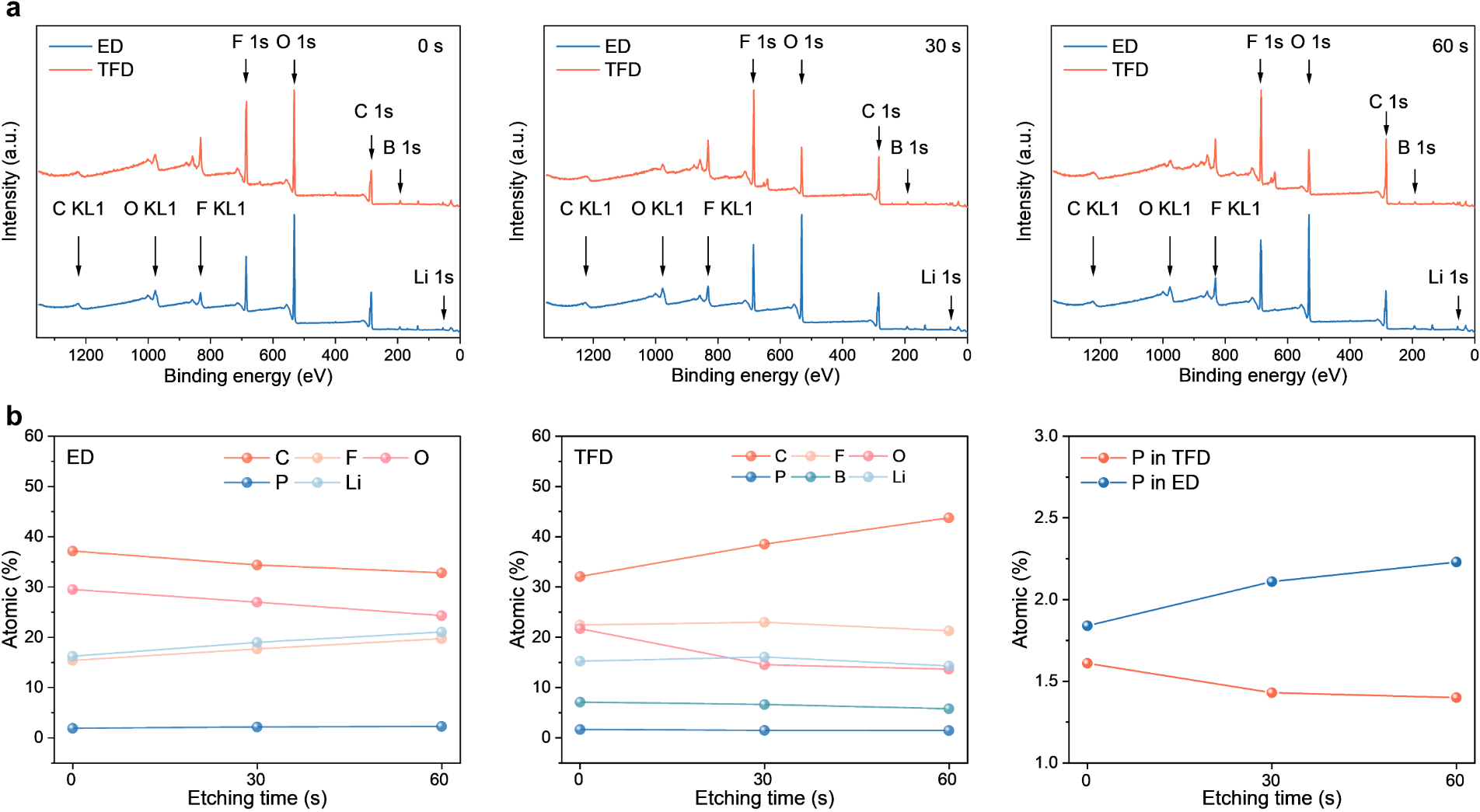
**Figure S26.** **a** The XPS full spectra taken from cycled LRMO cathode in ED and TFD at different etching time. **b** Corresponding atomic concentration of various elements for XPS depth results in **a**.


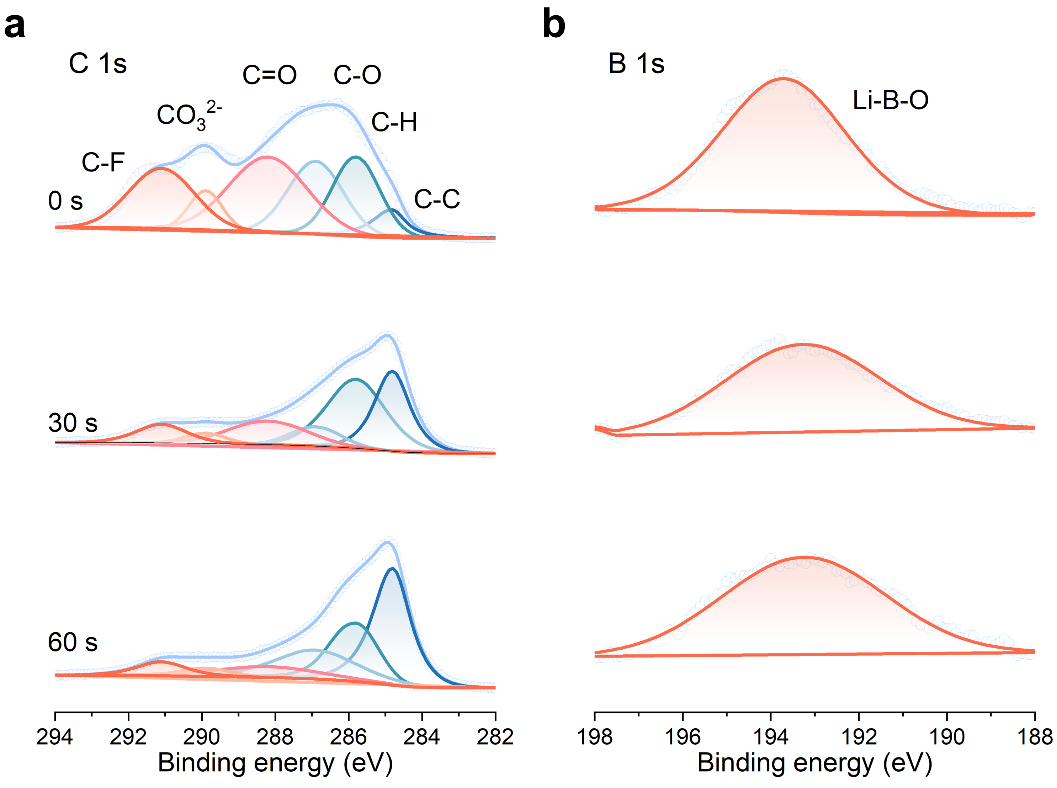


**Figure S27.** Detailed fitting XPS depth profiles of **a** C 1s, **b** B 1s in the LRMO cathode after 30 cycles in TFD.


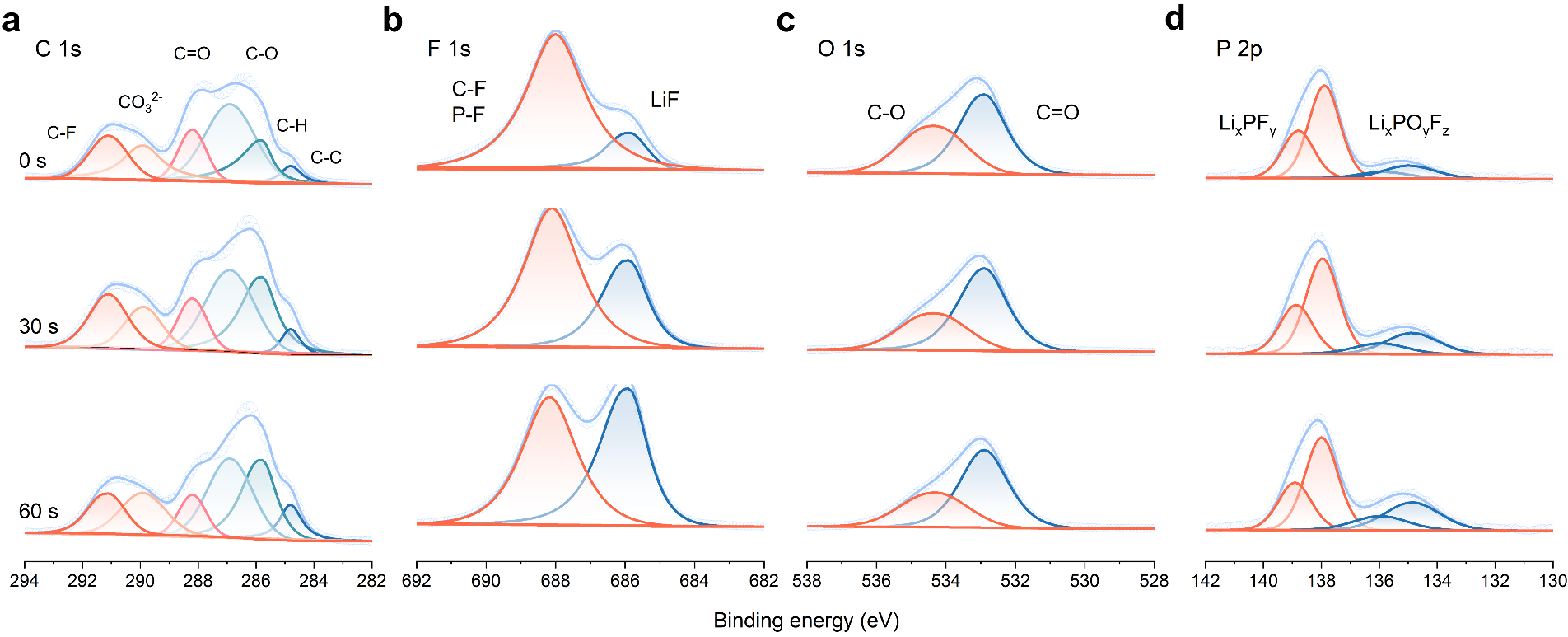


**Figure S28.** XPS depth profiles of the LRMO cathode after 30 cycles in ED and detailed fitting results of **a** C 1s, **b** F 1s, **c** O 1s, and **d** P 2p.


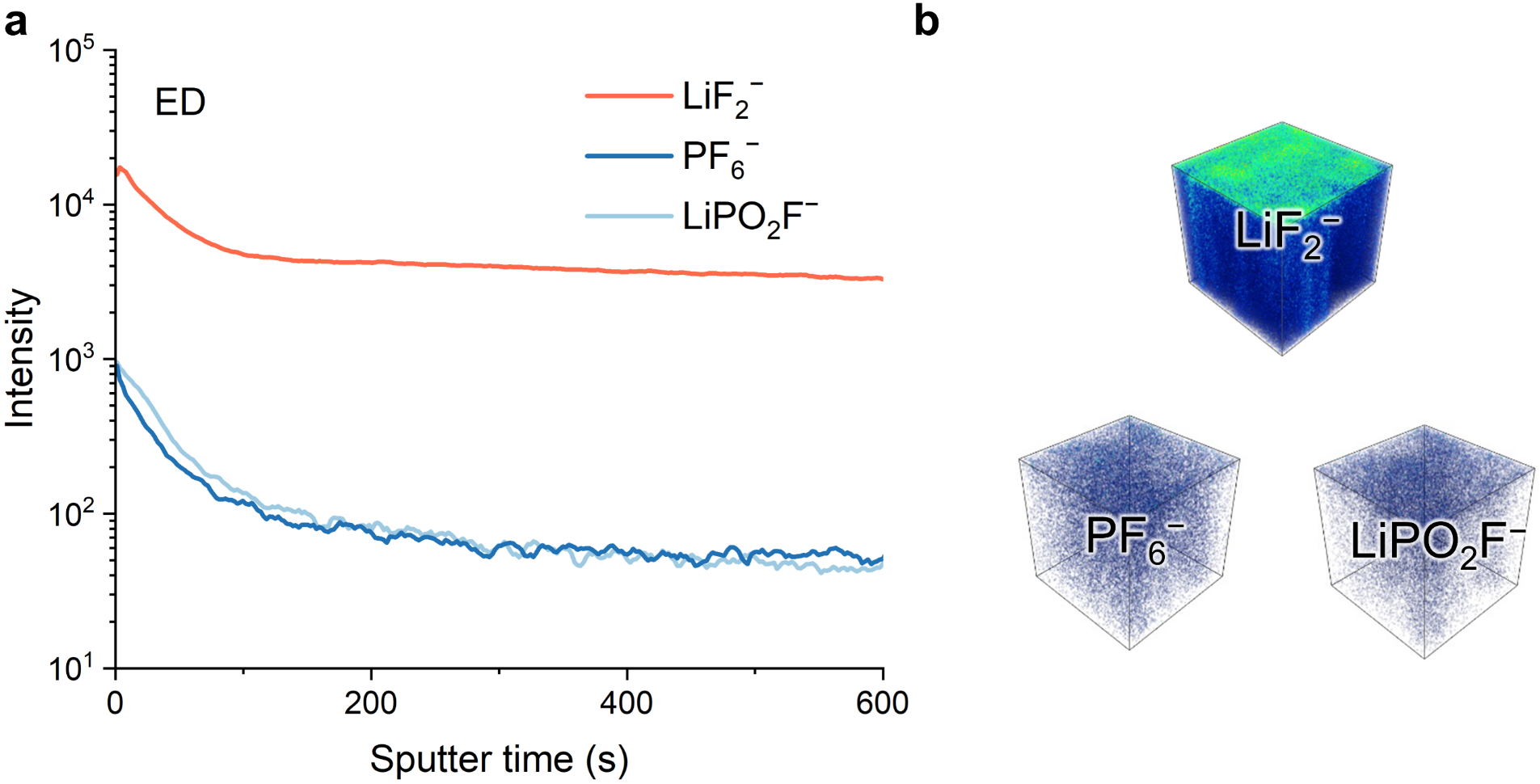


**Figure S29.** Sputter depth profiles of various secondary ions obtained by TOF-SIMS and corresponding 3D reconstruction images taken from cycled LRMO cathode in ED.


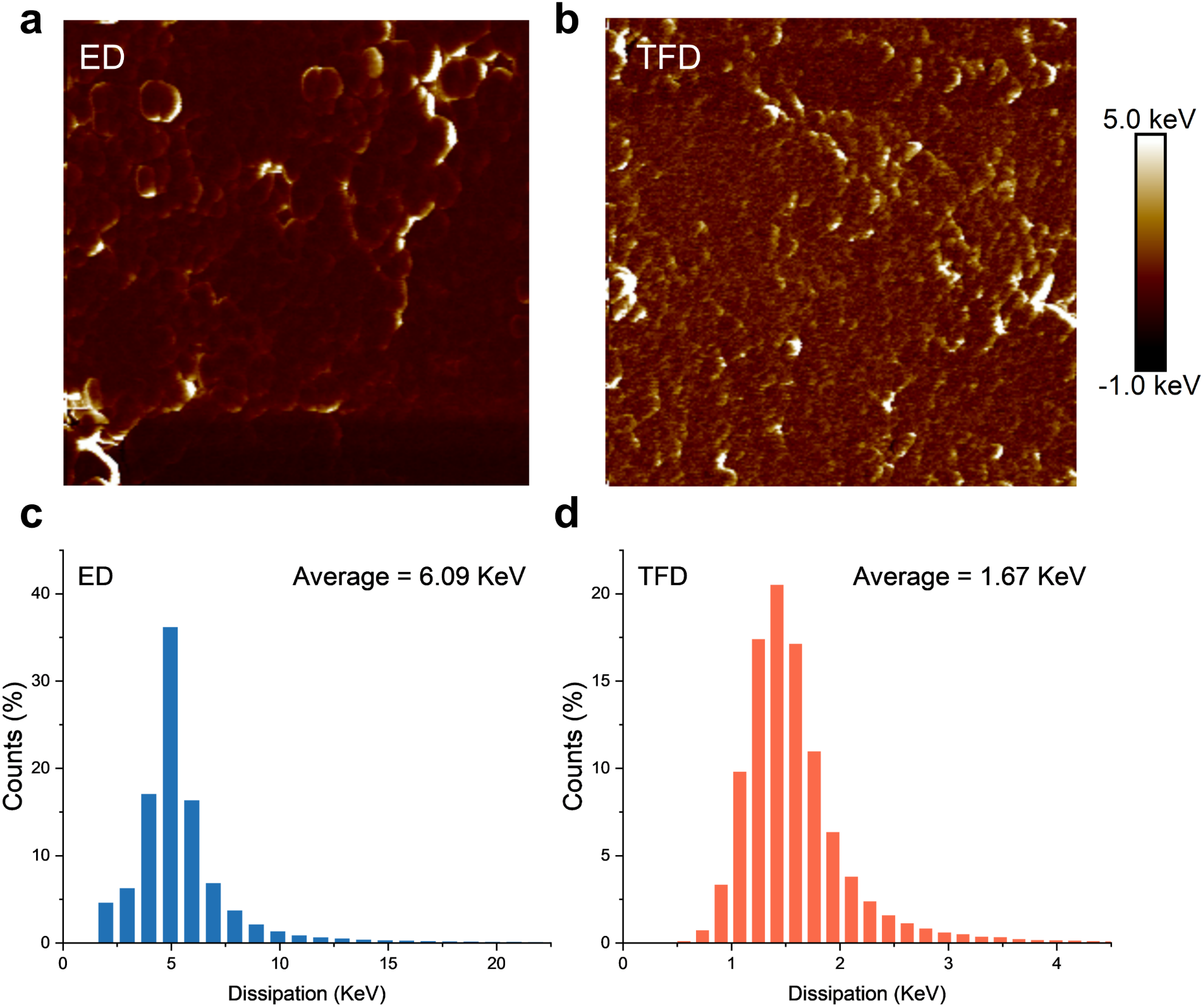


**Figure S30.** **a** The dissipation images of LRMO cathodes cycled in ED and TFD electrolyte. **b** Corresponding dissipation distribution of **a**.


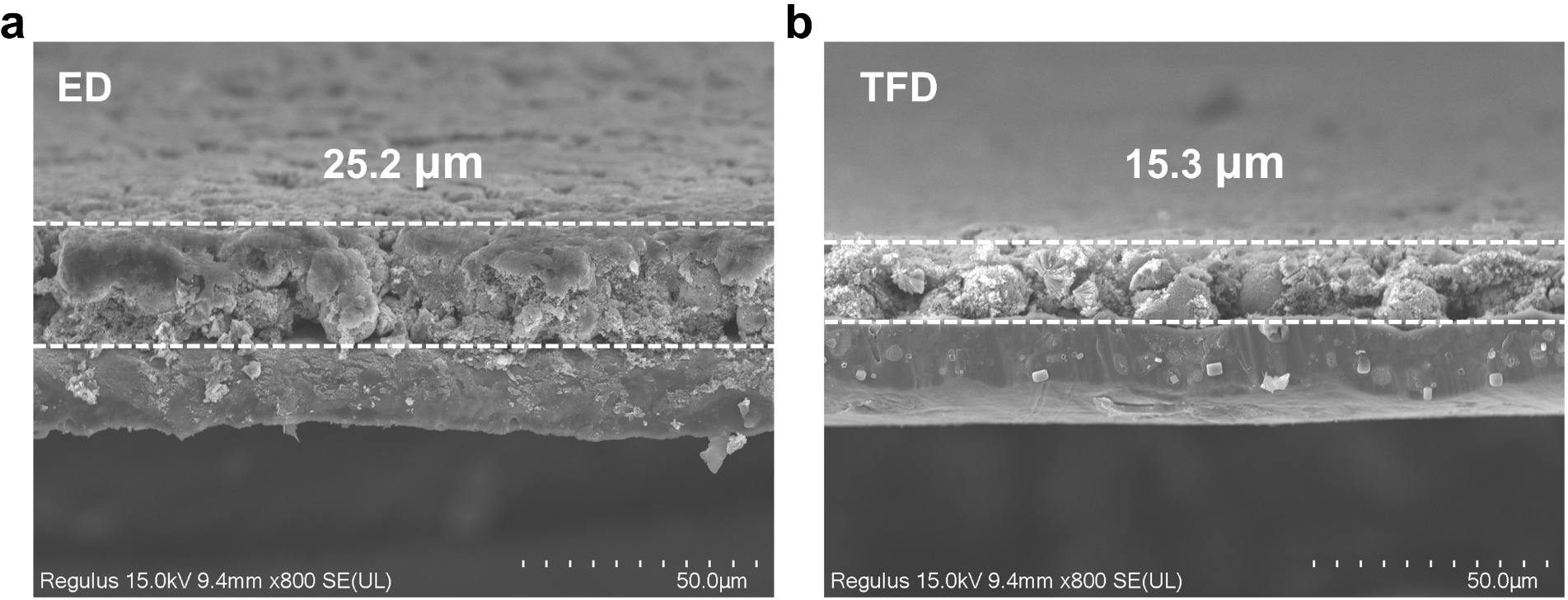


**Figure S31.** Cross-sectional-view SEM images of LRMO cathodes after 30th cycles in **a** ED, **b** TFD.


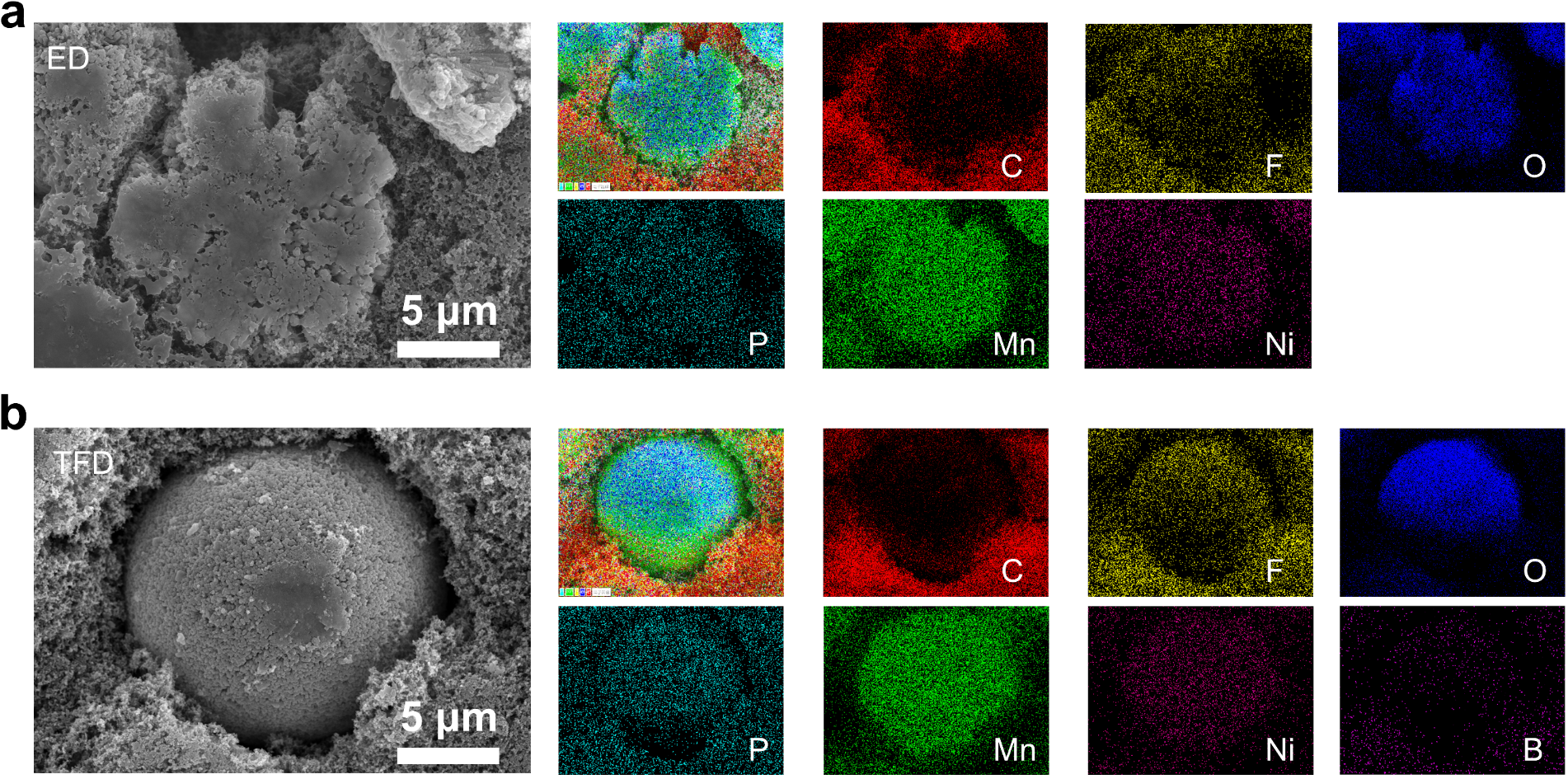


**Figure S32** Top-view SEM images and Corresponding EDS mapping results of LRMO cathodes after 30th cycles in **a** ED, **b** TFD.


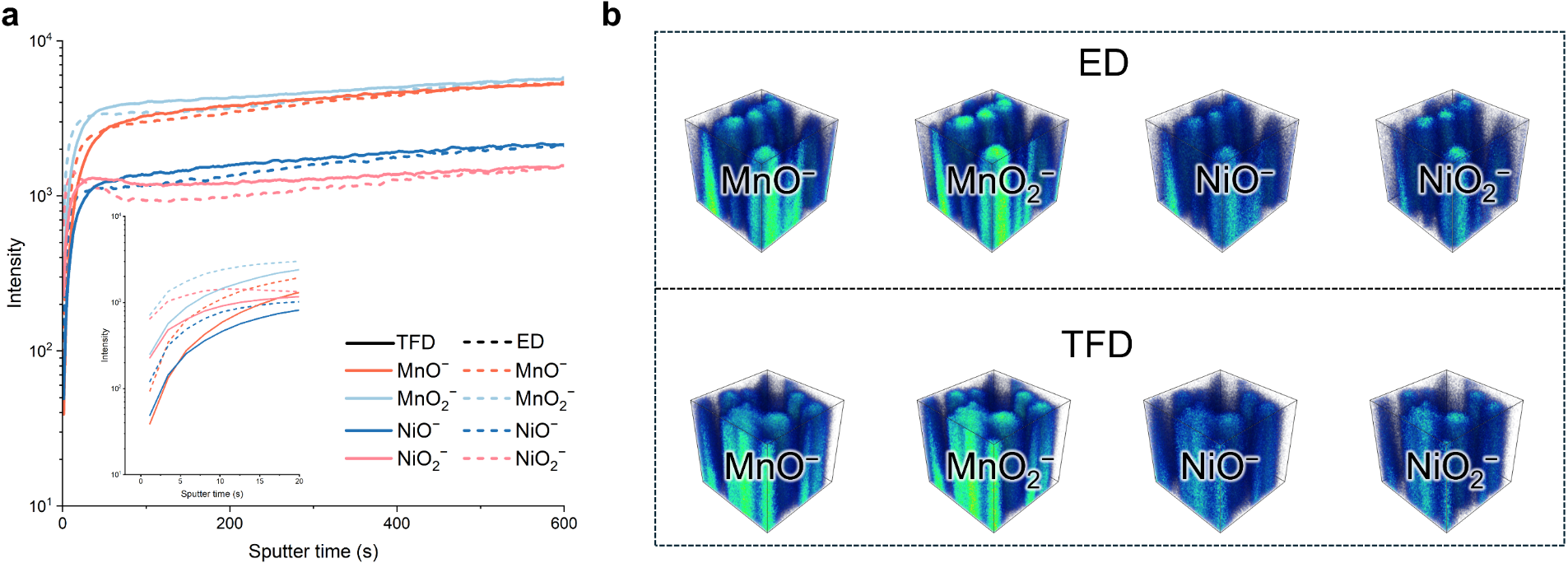


**Figure S33.** **a** Sputter depth profiles of various secondary ions obtained by TOF-SIMS. **b** Corresponding 3D reconstruction images taken from cycled LRMO cathode in ED and TFD. As shown in the inset of **a**, higher signals of Mn/Ni-related species were detected on the ED surface, indicating severe Mn/Ni dissolution. With increasing etching time, a further decrease in these signals was observed, corresponding to pronounced Mn/Ni loss from the bulk structure. In contrast, this phenomenon was effectively mitigated in the LRMO cathode cycled with TFD electrolyte.


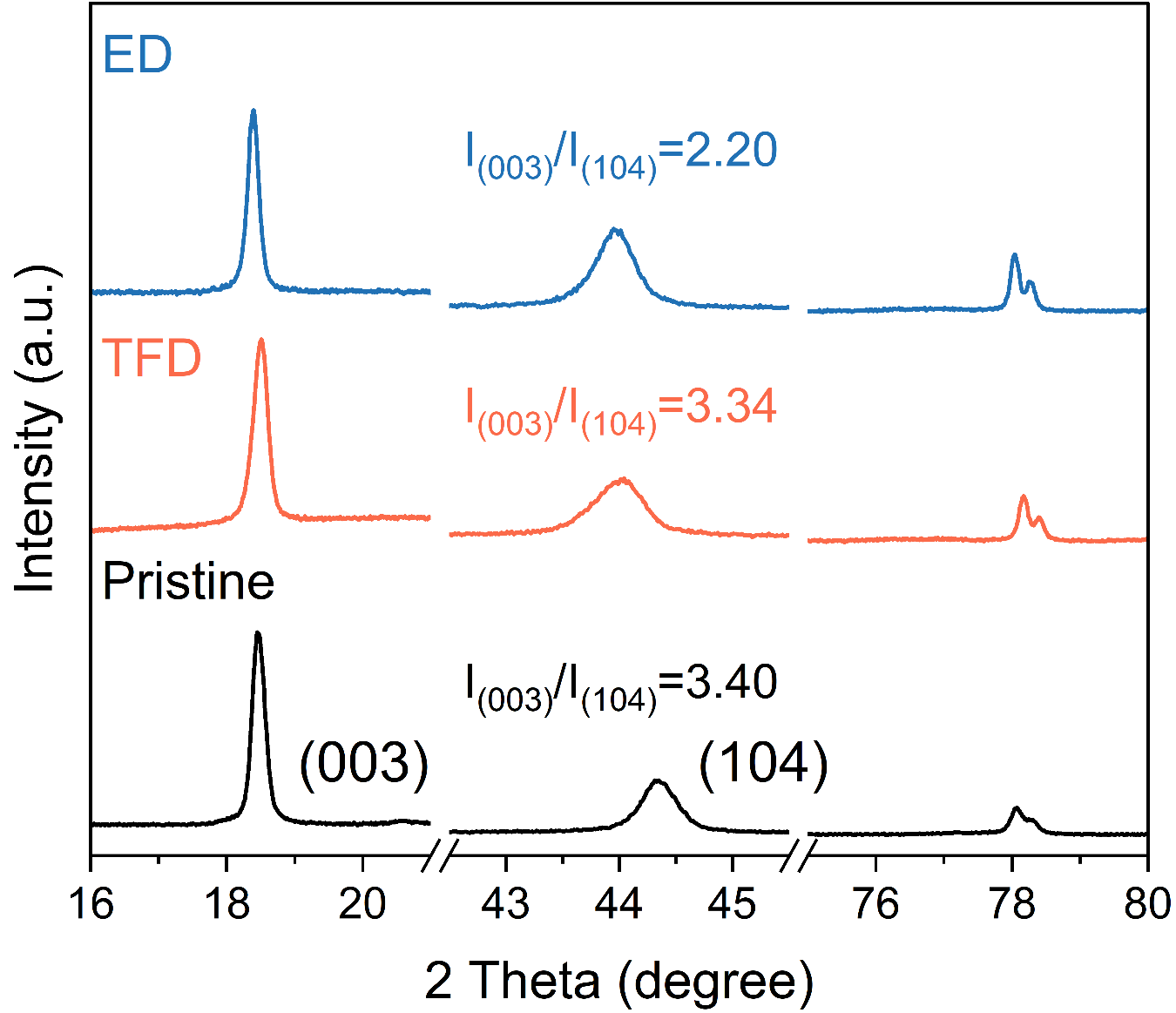


**Figure S34.** Ex situ XRD measurements of the LRMO cathode before and after 30 cycles in ED and TFD electrolytes.


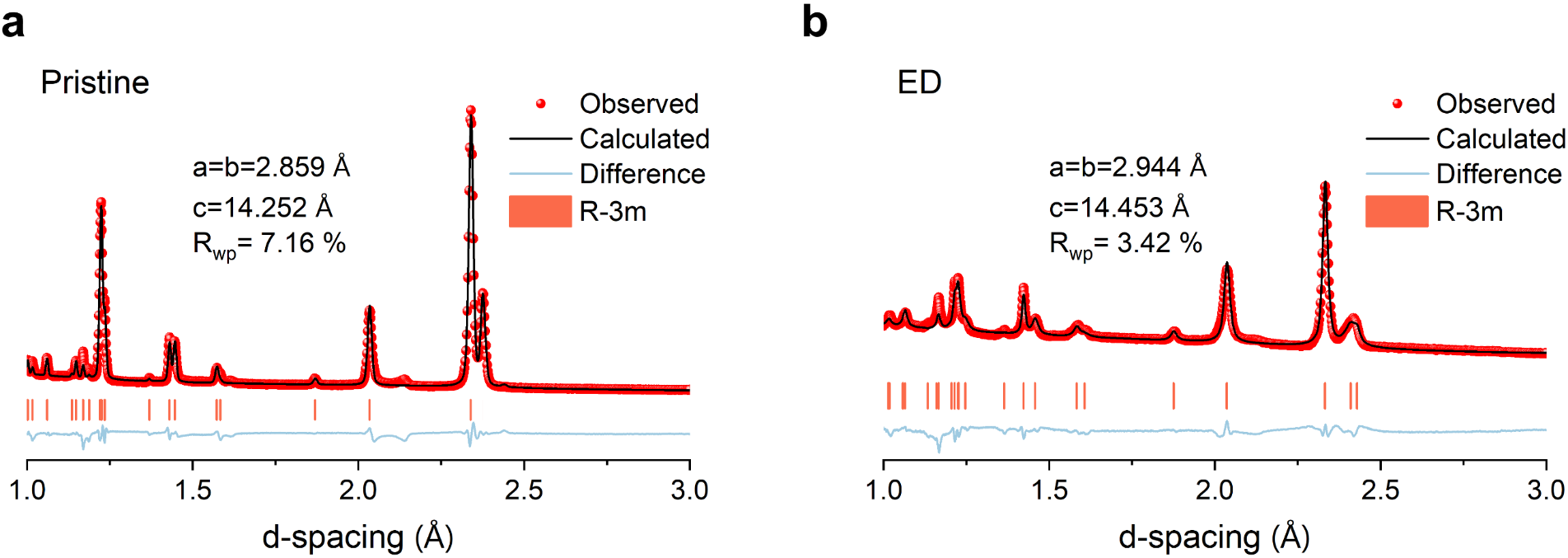


**Figure S35.** NPD Rietveld refinement of **a** Pristine LRMO cathode, and **b** LRMO cathode cycled in ED.


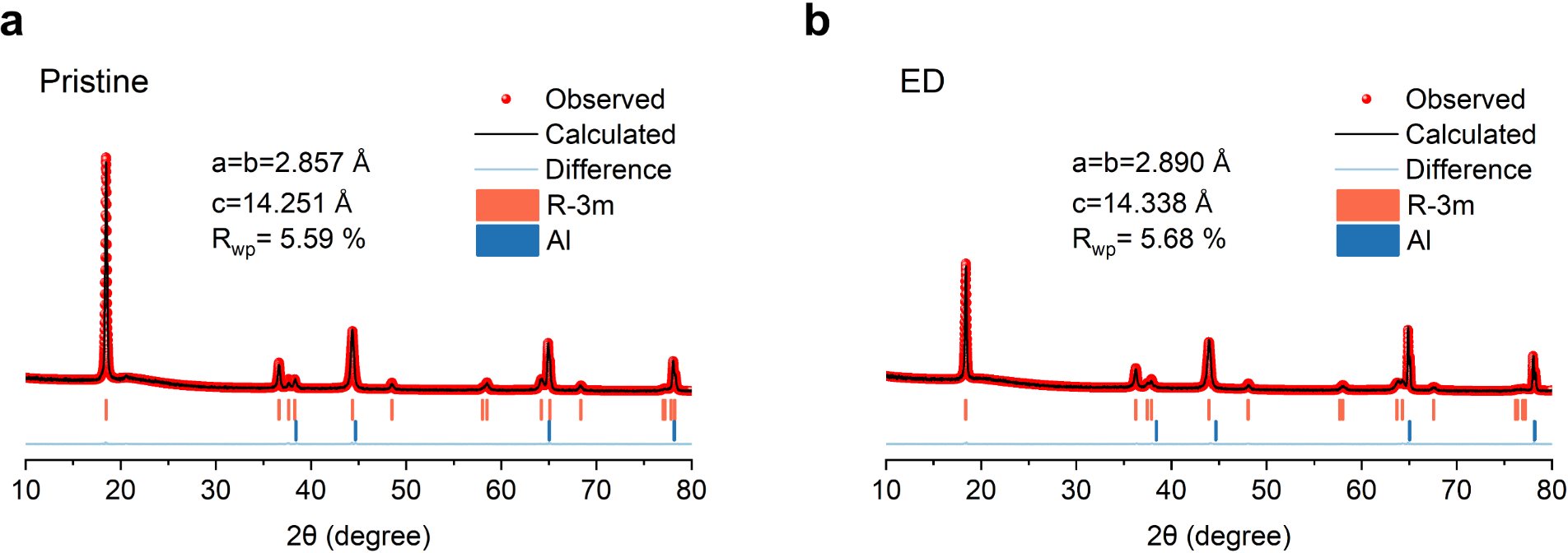


**Figure S36.** XRD Rietveld refinement of **a** Pristine LRMO cathode, and **b** LRMO cathode cycled in ED.


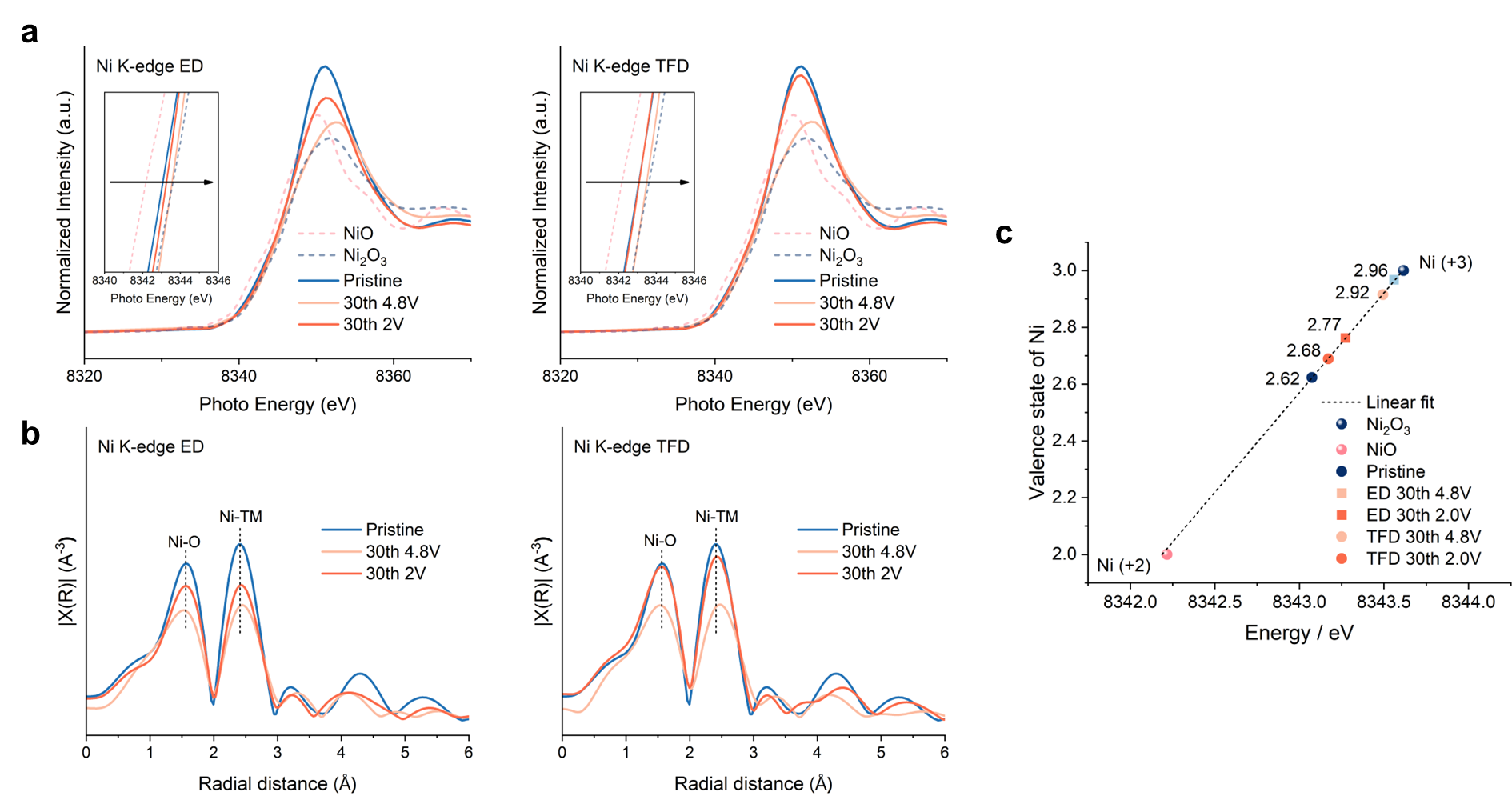


**Figure S37.** **a** Ni K-edge XANES of LRMO cathode cycled with ED and TFD under various operating conditions. **b** Fourier transformed EXAFS spectra of Ni K-edge XANES. **c** Calculated valence states of Ni from Ni K-edge XANES data.


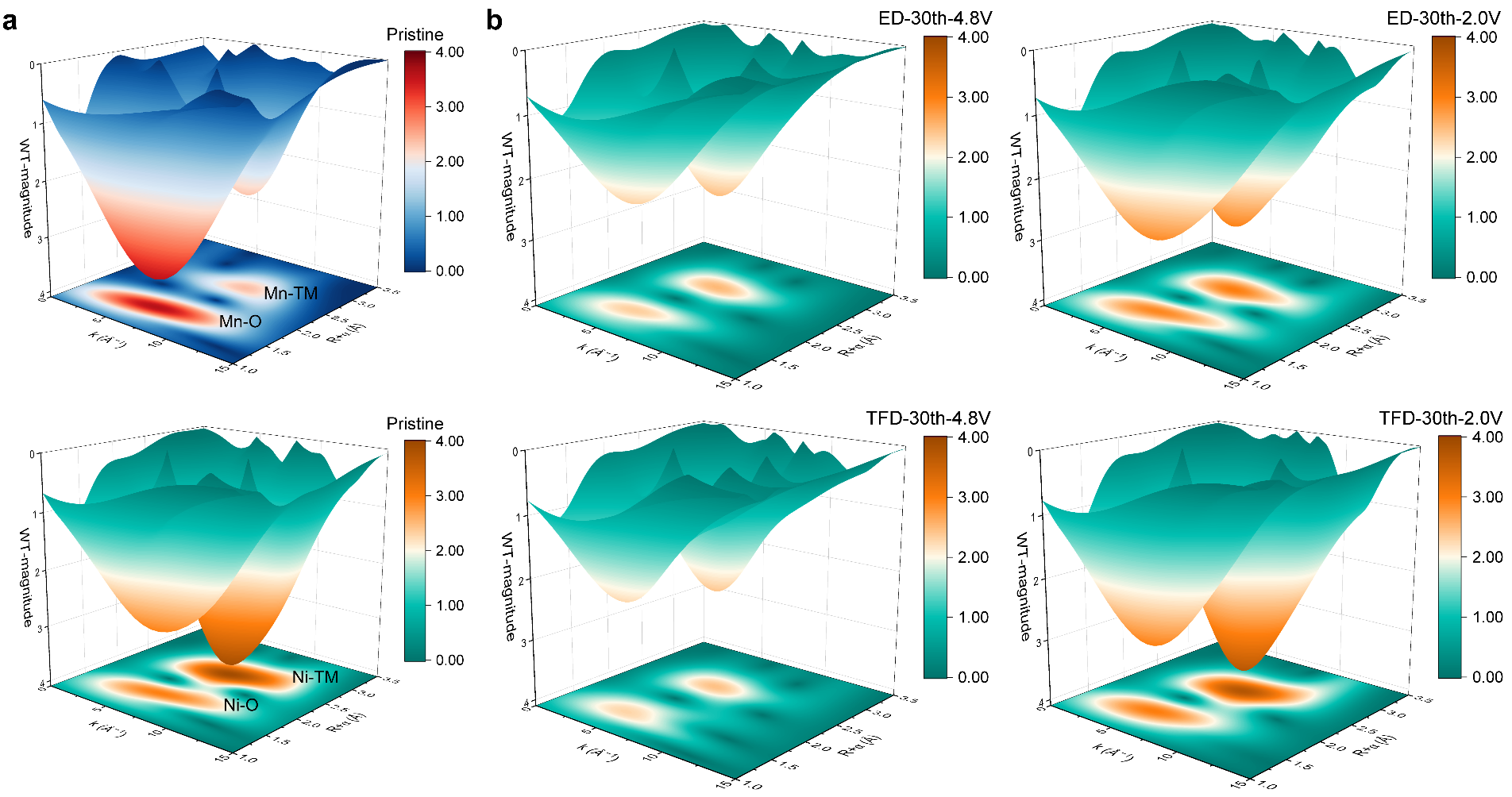
**Figure S38.** **a** WT fitting results of Mn K-edge and Ni K-edge XANES of Pristine LRMO cathode. **b** WT fitting results of Ni K-edge EXAFS spectra at different conditions of LRMO cathode cycled with ED and TFD.


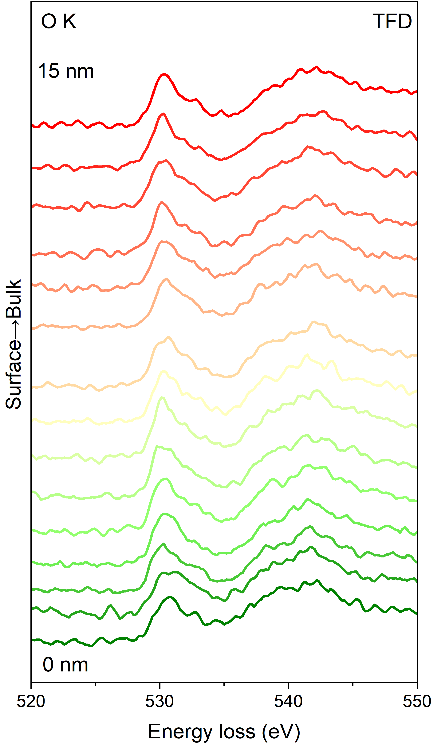

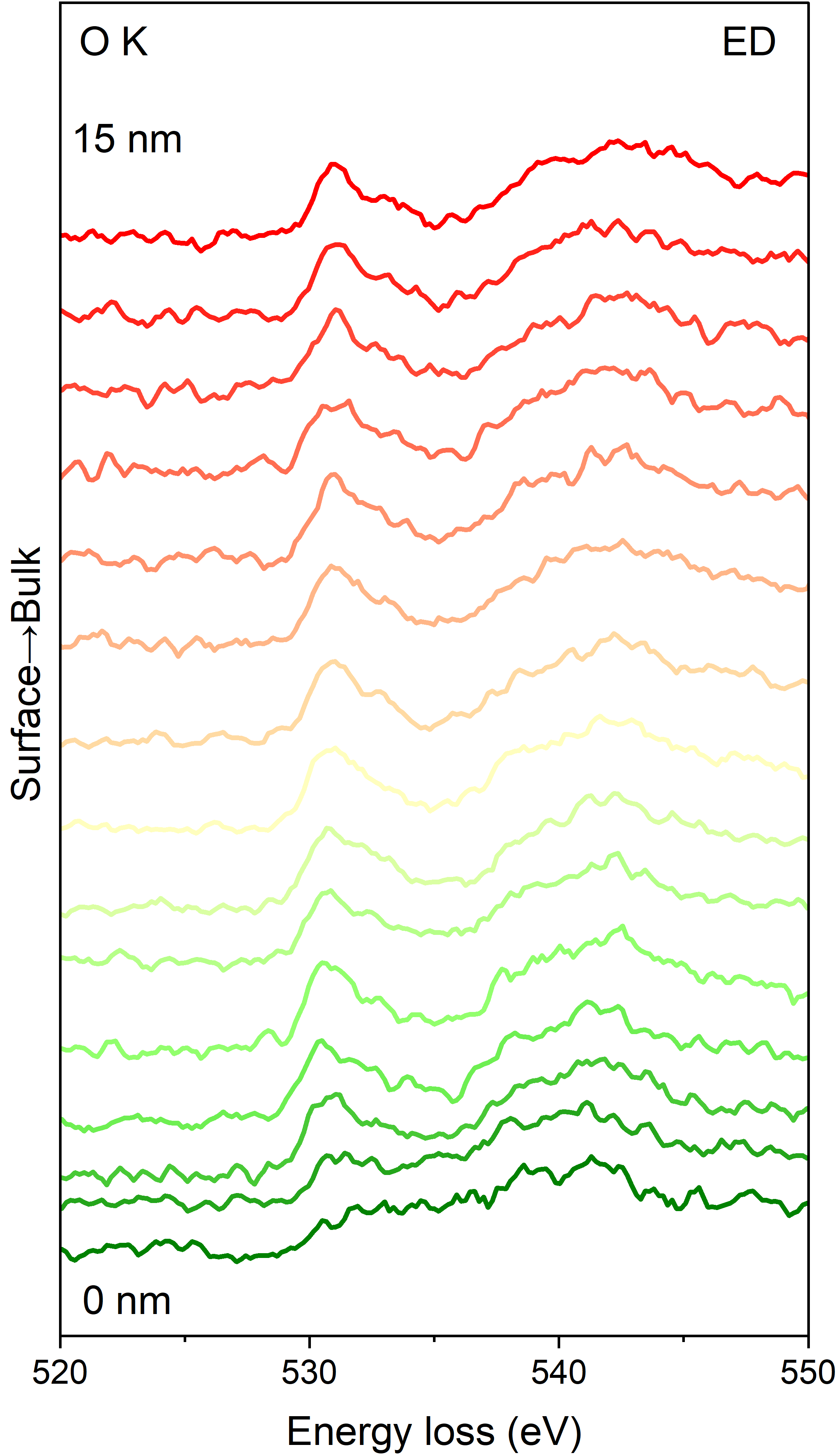


**b**

**a**

**d**

**c**


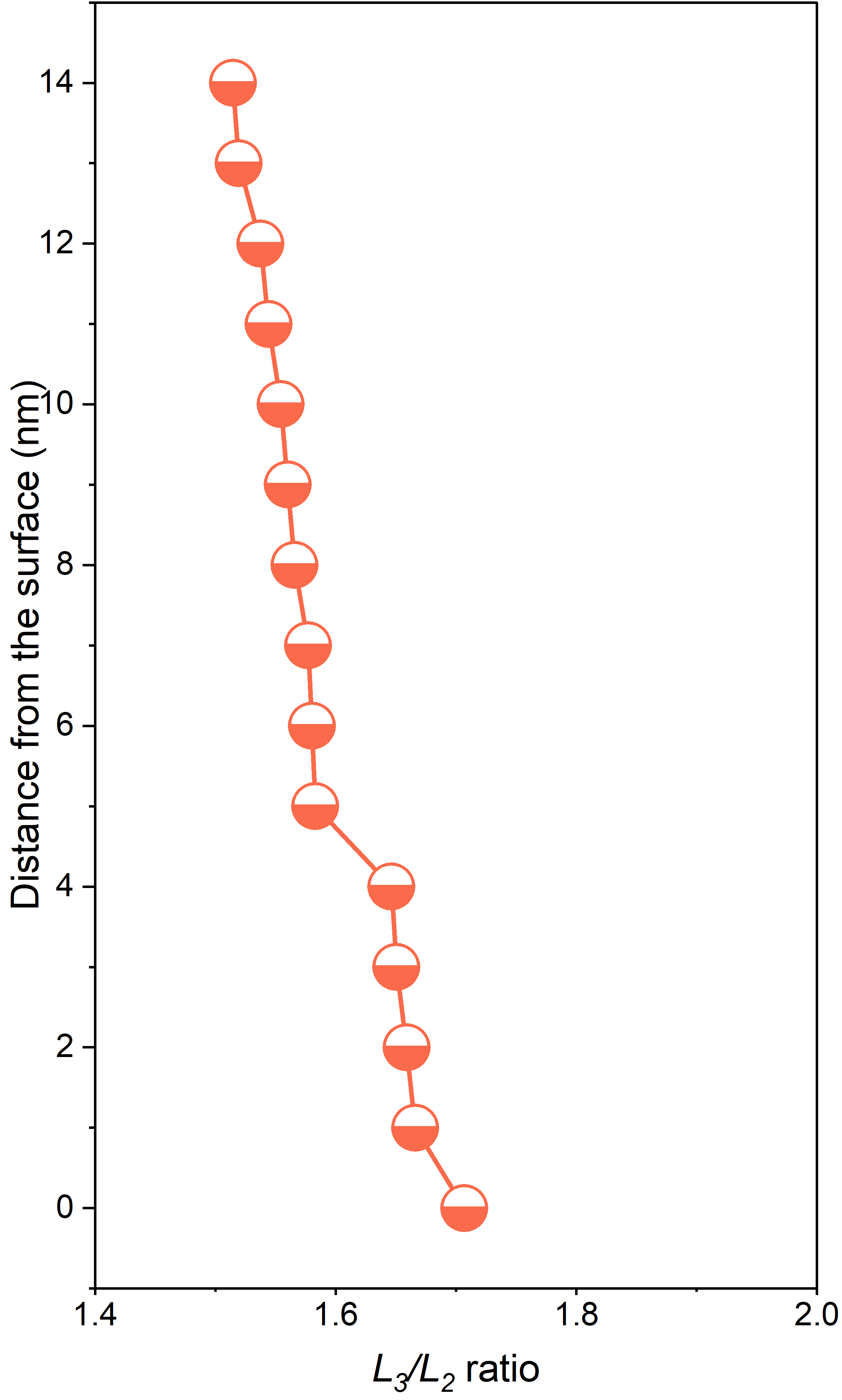

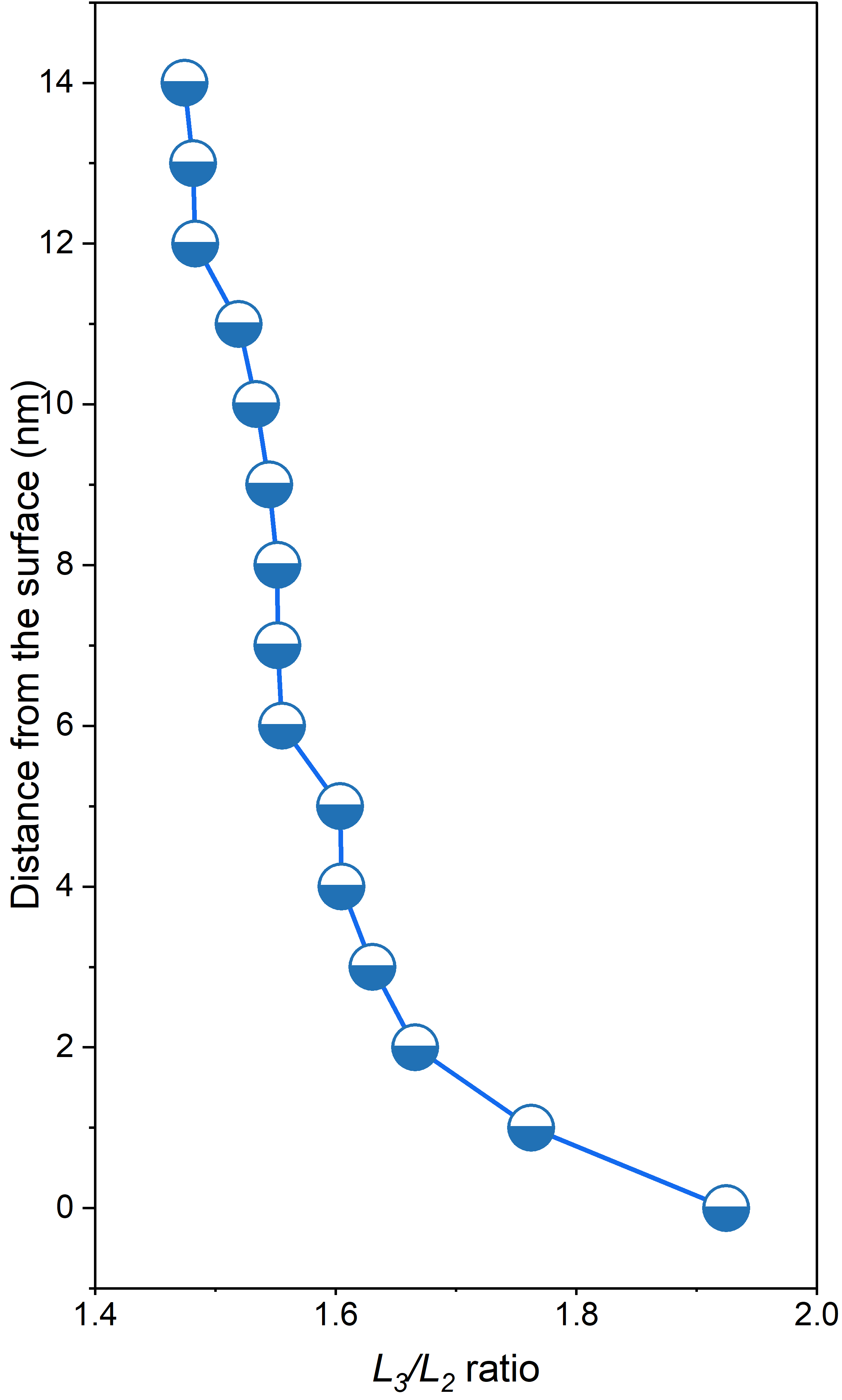


**Figure S39.** EELS spectra of O K-edge from the surface to the inner bulk for LRMO cathode cycled in **a** ED, and **b** TFD. The *L_3_/L_2_* ratio extracted from EELS spectra of Mn L-edge from the surface to the inner bulk for LRMO cathode cycled in **h** ED, and **i** TFD.


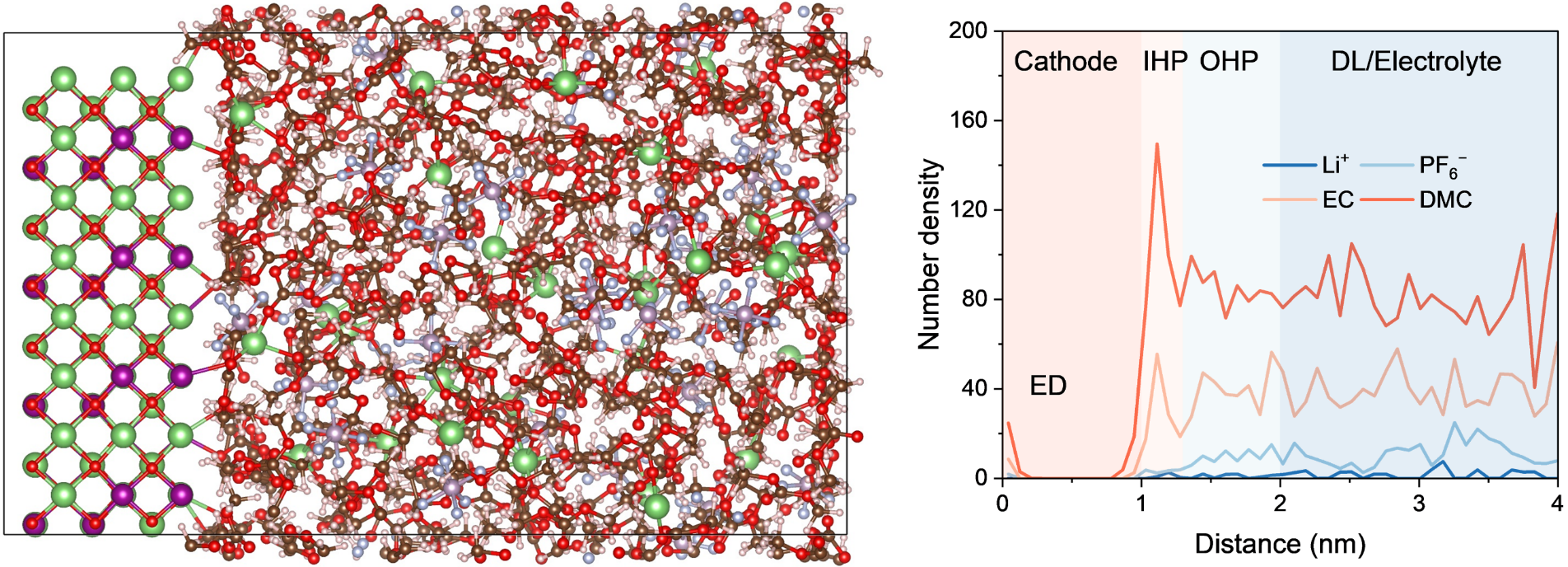


**Figure S40.** Snapshot and corresponding EDL distribution at the Li_2_MnO_3_/ED interface extracted from MD simulation.


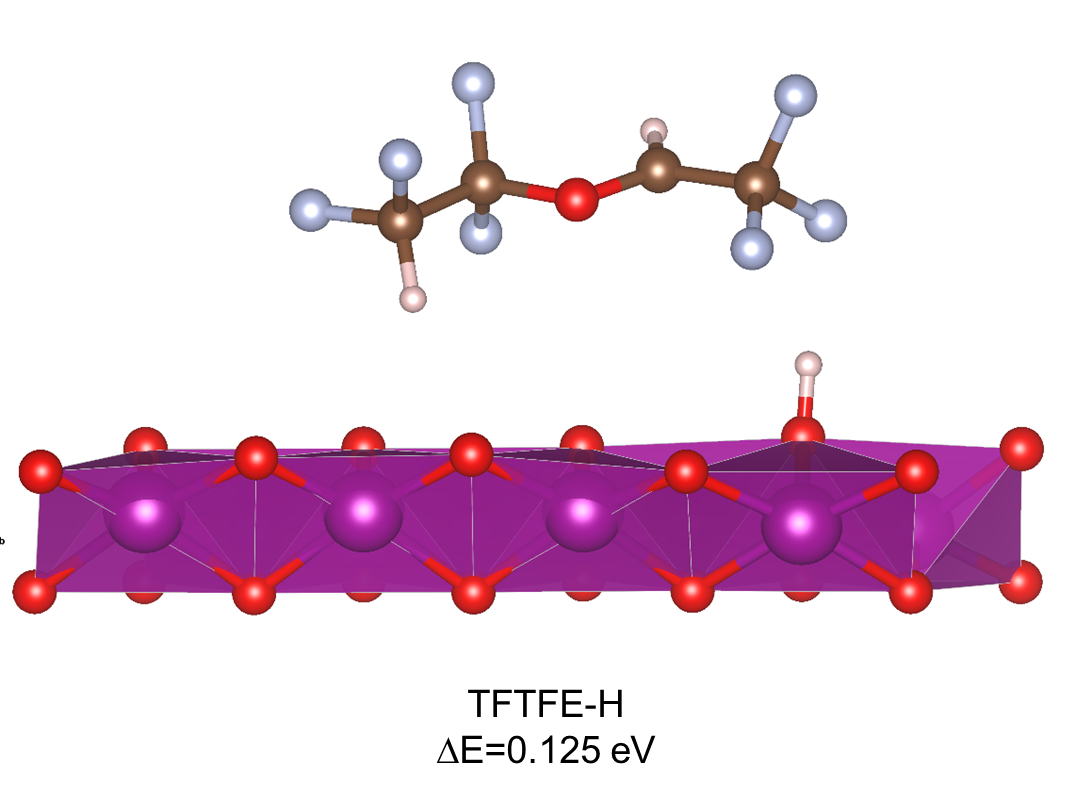


**Figure S41.** Computational results of H-transfer energies of DMC, DFEA, EC and FEC on the surface of highly delithiated LRMO surface.

**References**

[1] M. J. Abraham, T. Murtola, R. Schulz, S. Páll, J. C. Smith, B. Hess, E. Lindahl, *SoftwareX* **2015**, 1-2, 19.

[2] L. Martínez, R. Andrade, E. G. Birgin, J. M. Martínez, *J. Comput. Chem.* **2009**, 30, 2157.

[3] T. Darden, D. York, L. Pedersen, *J. Chem. Phy.* **1993**, 98, 10089.

[4] W. Brand, B. Oosterhuis, P. Krajcsi, D. Barron, F. Dionisi, P. J. van Bladeren, I. M. C. M. Rietjens, G. Williamson, *Biopharm. Drug Dispos.* **2011**, 32, 530.

[5] H. J. C. Berendsen, J. P. M. Postma, W. F. v. Gunsteren, A. DiNola, J. R. Haak, *J. Chem. Phys.* **1984**, 81, 3684.

[6] M. J. Frisch, G. W. Trucks, H. B. Schlegel, G. E. Scuseria, M. A. Robb, J. R. Cheeseman, G. Scalmani, V. Barone, G. A. Petersson, H. Nakatsuji, et al., Gaussian 16, Revision A.03, Gaussian, Inc., Wallingford, CT 2016.

[7] A. D. Becke, *J. Chem. Phys.* **1993**, 98, 5648.

[8] L. Xing, W. Li, C. Wang, F. Gu, M. Xu, C. Tan, J. Yi, *J. Phys. Chem. B* **2009**, 113, 16596.

[9] T. Lu, F. Chen, *J. Comput. Chem.* **2012**, 33, 580.

[10] W. Humphrey, A. Dalke, K. Schulten, *Journal of Molecular Graphics* **1996**, 14, 33.

[11] G. Kresse, J. Furthmüller, *Phys. Rev. B* **1996**, 54, 11169.

[12] P. E. Blöchl, *Phys. Rev. B* **1994**, 50, 17953.

[13] J. P. Perdew, K. Burke, M. Ernzerhof, *Phys. Rev. Lett.* **1996**, 77, 3865.
